# The evolution of climate tolerance in conifer‐feeding aphids in relation to their host's climatic niche

**DOI:** 10.1002/ece3.5652

**Published:** 2019-10-02

**Authors:** Pierre Arnal, Armelle Coeur d'acier, Colin Favret, Martin Godefroid, Ge‐Xia Qiao, Emmanuelle Jousselin, Andrea Sanchez Meseguer

**Affiliations:** ^1^ CBGP INRA CIRAD IRD Montpellier SupAgro Univ Montpellier Montpellier France; ^2^ Institut Systématique Evolution Biodiversité (ISYEB) Muséum national d'Histoire naturelle CNRS EPHE Sorbonne Université Paris France; ^3^ Department of Biological Sciences Biodiversity Centre University of Montreal Montreal QC Canada; ^4^ Key Laboratory of Zoological Systematics and Evolution Institute of Zoology Chinese Academy of Sciences Beijing China; ^5^ CNRS UMR 5554 Institut des Sciences de l'Evolution (ISEM) Univ Montpellier Montpellier France

**Keywords:** climate, insect–plant interactions, niche equivalency tests, niche evolution, phylogeny, phytophagous insects

## Abstract

Climate adaptation has major consequences in the evolution and ecology of all living organisms. Though phytophagous insects are an important component of Earth's biodiversity, there are few studies investigating the evolution of their climatic preferences. This lack of research is probably because their evolutionary ecology is thought to be primarily driven by their interactions with their host plants. Here, we use a robust phylogenetic framework and species‐level distribution data for the conifer‐feeding aphid genus *Cinara* to investigate the role of climatic adaptation in the diversity and distribution patterns of these host‐specialized insects. Insect climate niches were reconstructed at a macroevolutionary scale, highlighting that climate niche tolerance is evolutionarily labile, with closely related species exhibiting strong climatic disparities. This result may suggest repeated climate niche differentiation during the evolutionary diversification of *Cinara*. Alternatively, it may merely reflect the use of host plants that occur in disparate climatic zones, and thus, in reality the aphid species' fundamental climate niches may actually be similar but broad. Comparisons of the aphids' current climate niches with those of their hosts show that most *Cinara* species occupy the full range of the climatic tolerance exhibited by their set of host plants, corroborating the hypothesis that the observed disparity in *Cinara* species' climate niches can simply mirror that of their hosts. However, 29% of the studied species only occupy a subset of their hosts' climatic zone, suggesting that some aphid species do indeed have their own climatic limitations. Our results suggest that in host‐specialized phytophagous insects, host associations cannot always adequately describe insect niches and abiotic factors must be taken into account.

## Graphical abstract

In this study, we use a robust phylogenetic framework and a species‐level distribution data for the conifer‐feeding aphid genus *Cinara* to investigate the role of climatic adaptation in the diversity and distribution patterns of these host‐specialised insects. Our results suggest that in host‐specialized phytophagous insects, host associations cannot always adequately describe insect niches and abiotic factors must be taken into account.

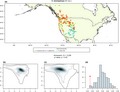

## 1 INTRODUCTION

The niche theory is a central concept in ecology and evolution (Duran & Pie, 2015; Pearman, Guisan, Broennimann, & Randin, 2008; Pearman et al., 2014; Petitpierre et al., 2012; Wiens et al., 2010), with the set of ecological conditions where species occur (Hutchinson, 1957) considered one of the key factors that drive diversification and biogeographic patterns on Earth. With more than 500,000 known species, phytophagous insects represent nearly a quarter of the whole terrestrial macroscopic biodiversity (Daly, Doyen, & Purcell, 1998; Southwood & Norton, 1973; Strong, Lawton, & Southwood, 1984). However, the factors that shape the geographic distribution of phytophagous insects are still poorly understood, limiting our ability to model the effects of climate change on their future distributions; there is also an additional complexity of combining the insects' and host plants' climate niches (Barredo et al., 2015).

Most phytophagous insect species are host‐specific, feeding on one or a few plant species (Jaenike, 1990), and thus, their ecology and life history are strongly dependent on their interaction with their host plants. Consequently, the study of phytophagous insect niches is often restricted to the traits characterizing their association with their host plants: the range of plant species they feed on (Kaplan & Denno, 2007), the plant organs on which they develop (Condon & Steck, 1997; Cook, Rokas, Pagel, & Stone, 2002), or the range of natural enemies encountered on these plants (Singer & Stireman, 2005). This traditional host‐centered conception of the ecological niche of phytophagous insects implies that their distribution is primarily constrained by the geographic range of their hosts, whereas other environmental abiotic factors, such as geographic or climatic barriers, are rarely taken into account (Futuyma & Moreno, 1988; Jaenike, 1990). Similarly, insect diversification and biogeographic history are also often interpreted as the consequence of host shifts (von Dohlen, Kurosu, & Aoki, 2002; Janz, Nylin, & Wahlberg, 2006; Nyman, Linder, Peña, Malm, & Wahlberg, 2012).

In the last decades, the study of the environmental conditions relevant to large‐scale ecological and geographic properties of species has been conducted in numerous organisms such as amphibians (Bonetti & Wiens, 2014; Kozak & Wiens, 2010), squamates (Knouft, Losos, Glor, & Kolbe, 2006; Pyron & Burbrink, 2009), plants (Evans, Smith, Flynn, & Donoghue, 2008; Meseguer, Lobo, Ree, Beerling, & Sanmartín, 2014), mammals (Dormann, Gruber, Winter, & Herrmann, 2009), and birds (Pearman et al., 2014). These reveal an important role for climate adaptation in the evolution of these organisms. The climate niche reflects the physiological tolerance to climatic conditions (Grinnell, 1917) and may strongly influence the distribution of species over space and time (Soberón, 2007). Unfortunately, climatic niche evolution has been scarcely investigated in phytophagous insects (but see Kellermann et al., 2012; Pitteloud et al., 2017) and we still have a limited understanding of the distribution in climate space of related insect species. This relative neglect may be due to the difficulty of gathering climatic distribution data for an entire clade of phytophagous insects, but it may also stem from the assumption that their distributions simply reflect host plant distributions and are therefore uninteresting in their own right.

Phytophagous insect development, survival, and capacity to exploit plants are known to be sensitive to climate conditions, and insects do exhibit many climate dependent traits (Bale et al., 2002; Gallego, Verdú, Carrascal, & Lobo, 2016; Howe, 1967; Régnière, Powell, Bentz, & Nealis, 2012). For example, Duyck, David, and Quilici (2006) showed that *Ceratitis* fruit fly pupae exhibit different climatic niches, coexisting on the French island of La Réunion in the southwest Indian Ocean under different humidity conditions. Another telling example is the hemlock woolly adelgid (*Adelges tsugae*), an invasive insect introduced to eastern North America that is decimating populations of eastern and Carolina hemlock (*Tsuga canadensis* and *T. caroliniana*) from Georgia to Connecticut. The success of its invasion is limited by its low cold tolerance: several *T. canadensis* and *T. caroliniana* populations distributed in colder regions have not been impacted by the pest (Paradis, Elkinton, Hayhoe, & Buonaccorsi, 2008). It is also well known that several insect species are tracking cooler habitats or hatching earlier than they used to in response to climate warming (Visser & Holleman, 2001). Finally, studies have shown that some phytophagous insects occupy only a part of the range of their host plants (Battisti et al., 2005; Carroll, Taylor, Régnière, & Safranyik, 2003; Godefroid et al., 2016; Hill & Hodkinson, 1992), suggesting that climate variables, such as temperature and precipitation, may play a significant role in defining the insects' habitat and distribution. Altogether, these studies provide evidence that phytophagous insects can display adaptations to specific climate conditions.

Here, we investigated the role of climate adaptation in the long‐term evolution of a clade of aphids (Hemiptera; Aphididae) of the genus *Cinara*, in relation to their host plants. We sought to answer the following general questions: (a) Does this phytophagous insect lineage exhibit long‐term climatic specialization? (b) Do aphids occur in the full range of climate conditions occupied by their host plants, or do they only occupy a subset of the host's climatic niche? Concordance between the insects' and hosts' climatic niches would suggest that observed insect climatic niches are limited by the hosts' presence rather than their own set of physiological constraints. Aphids are particularly well‐suited to address these questions as they are generally host‐specific, they spend their whole life cycle on their hosts, and their range of host preference and their geographic distribution are generally well‐known due to the pest status of numerous species. Using a georeferenced occurrence dataset and a comprehensive phylogeny, we studied the evolution of climate tolerance of the conifer‐feeding aphid genus *Cinara* Curtis, 1835 (Aphididae: Hemiptera) in relation to their *Pinus* spp. hosts.

## 2 MATERIAL AND METHODS

### 2.1 Study system, distribution and bioclimatic data

The genus *Cinara* (Aphididae: Lachninae) includes 253 described species, including those of the recently subordinated subgenus *Schizolachnus* (Chen, Favret, Jiang, Wang, & Qiao, 2016; Meseguer, Coeur d'acier, Genson, & Jousselin, 2015). *Cinara* species have their native range exclusively within the Northern Hemisphere, and they are generally host‐specific, feeding on one or a few plant species of the conifer families Pinaceae and Cupressaceae (Blackman & Eastop, 2000; Favret & Voegtlin, 2004). Associations with host plants are well known for most *Cinara* species as they have been the subject of many taxonomic investigations (Eastop, 1972; Favret & Voegtlin, 2004; Voegtlin, 1976), and these association have been reviewed and compiled in the reference book by Blackman and Eastop (1994) which is regularly updated (Blackman & Eastop, 2019). We compiled a total of 6,135 *Cinara* occurrence records for 106 species. These include data from online databases (GBIF with 2,359 records and IBOL with 1,334 records) and aphid taxonomic databases from the entomological collection of the University of Montreal (1,705 records), the Institute of Zoology of the Chinese Academy of Sciences (221 records), and the INRA collection (516 records). All occurrences were reviewed and ambiguous accessions (e.g., occurrences for which the location names did not correspond with the given GPS coordinates or were assigned to county centroids) and duplicate records were removed. Occurrence data for *Pinus* species hosting *Cinara* were retrieved from the *Conifers of the World* database (Farjon & Filer, 2013).

In order to estimate the aphid climatic niches and those of their associated *Pinus* hosts, we selected a subset of the bioclimatic variables developed by Hijmans, Cameron, Parra, Jones, and Jarvis (2005), at the finest grid resolution (30s), that likely influence aphid biology: the maximum (BIO 5) and minimum (BIO 6) temperatures of the warmest and coldest months of the year; the mean temperature of the warmest and coldest quarters of the year (BIO 10 and BIO 11 respectively). Temperature variation affects insect development and reproduction (Ratte, 1984), including those of *Cinara* species (Durak & Borowiak‐Sobkowiak, 2013). The aphid life cycle is also likely affected if temperatures exceed threshold values, corresponding to heat stress or extreme cold (Campbell, Frazer, Gilbert, Gutierrez, & Mackauer, 1974). Because drought episodes can also have a significant effect on aphid colony development (Rouault et al., 2006), we also selected the precipitation of the driest month (BIO 17).

### 2.2 Taxonomic validation and association of occurrences with phylogenetic species

The identification of aphids based on morphological criteria can be ambiguous because aphids show considerable overlap in their morphological characteristics (Coeur d'acier et al., 2014). Specimen misidentification or other taxonomic ambiguity can lead to an erroneous geographic occurrence record of a species. We therefore sought to cure the occurrence dataset of misidentifications: We downloaded 743 *Cinara* barcode sequences (i.e., cytochrome oxidase I DNA sequences available in IBOL [http://www.boldsystems.org/] and GenBank [https://www.ncbi.nlm.nih.gov/genbank/]) representing 105 species and corresponding to 638 records in our occurrence dataset. Cross‐referencing the datasets allowed us to assess whether some of the species included in this study were not monophyletic and could thus be subject to taxonomic ambiguities. To this end, we reconstructed a maximum‐likelihood tree using a Kimura‐2‐parameter model of base substitution (Kimura, 1980) and 1,000 bootstrap replications as implemented in RaxML 8.2.10 (Stamatakis, 2014). The COI tree revealed some polyphyletic and paraphyletic species. In most cases, we defined monophyletic species groups of closely related species that seem to be regularly mixed. We then grouped occurrence data accordingly and assigned them to their respective species group. In cases where sequences assigned to a species were found in distant phylogenetic lineages, the species was either entirely removed from our occurrence dataset and the final phylogenetic tree, or just the evidently problematic occurrences were deleted. Details on the curation of the occurrence dataset and clustering of specimens into species groups are given in Appendices S1 and S2.

### 2.3 Phylogenetic methods and molecular dating

In order to obtain the most comprehensive *Cinara* phylogeny, we complemented the nucleotide dataset published by Meseguer et al. (2015) with sequences available on GenBank. We used one marker from the aphid nuclear genome (elongation factor 1‐α, EF) and two from its mitochondrion (COI; cytochrome b, CytB). Then, we considered two markers from the aphid's primary symbiont, *Buchnera aphidicola*: a chaperonin molecule involved in protein folding, GroEL, and “His” including the ATP phosphoribosultransferase, HisG, and the histidinol dehydrogenase, HisD. The final dataset included 82 *Cinara* species, 15 more than the Meseguer et al. (2015) study. Following Meseguer et al. (2015), 12 species belonging to seven genera of the Lachninae subfamily (*Lachnus*, *Longistigma*, *Maculolachnus*, *Pterochloroides*, *Stomaphis*, *Trama*, and *Tuberolachnus*) and two species belonging to two other aphid subfamilies (*Mindarus* sp.—Mindarinae and *Pemphigus populicaulis*—Eriosomatinae) were used as outgroups. Sequences were aligned using MUSCLE 3.8.31 (Edgar, 2004) followed by manual verification with BioEdit 7.2.5 (Hall, 1999).

Phylogenetic analyses were performed with the single concatenated DNA matrix with MrBayes 3.2.3 (Ronquist et al., 2012). Following MrModeltest (Nylander, 2004) results, Meseguer et al. (2015) had applied a GTR+INV+GAMMA model of substitution to each partition. We followed Meseguer et al. (2015), partitioning the nucleotide alignment by gene and applying the same GTR+INV+GAMMA substitution model to each partition. Two independent runs of four Metropolis‐coupled Monte Carlo Markov chains (MCMC) were run for 30 million generations, sampling trees and parameter values every 1,000 generations, with the first 25% of generations as burn‐in. Convergence was verified with the potential scale reduction factor (PSRF), calculated by MrBayes software, and ensuring the effective sample size (ESS) values was superior to 200 with Tracer 1.6 (Rambaut, Suchard, Xie, & Drummond, 2014). Results were summarized in a 50% majority‐rule consensus tree.

We used BEAST V.1.8.2 (Drummond & Rambaut, 2007) to estimate divergence times in the phylogeny using the topology inferred by MrBayes as a fixed tree. Following Meseguer et al. (2015), we used a Yule tree prior and a relaxed lognormal molecular clock. We first applied the GTR+G+I substitution model to all gene partitions but, detecting over‐parametrization for the GroEL and EF genes in Tracer (ESS < 200), we used the HKY+G+I model for these two markers. We ran two MCMC chains of 80 million generations each, sampled every 1,000 generations. We discarded 25% as burn‐in and combined the two independent runs with LogCombiner 1.8.3. Three calibration points were used to calculate absolute ages of divergence in *Cinara*, following Meseguer et al. (2015) (Table 1). Additionally, we performed the dating analysis with a random starting tree in order to obtain a posterior sample of trees while accounting for topological uncertainties.

**Table 1:** Calibrations use to estimate absolute age of divergence in *Cinara* phylogeny

1. *Fossil*: *Stomaphis eupetes* (Wegierek & Mamontova 1993)	2. *Fossil*: *Longistigma caryae* (Heie & Walter 1971)	3. *Aphididae common ancestor*
Age: Middle Miocene	Age: Late Miocene/early Pliocene	Dohlen and Moran (2000) estimated that the common ancestor of Aphididae was living 84–164 Ma years ago. We thus used a uniform 84–164 Ma prior to the root of the tree
Location: Caucasus	Location: Iceland	
Node: *Stomaphis* genus	Node: Longistigma genus	
Prior: lognormal with an offset of 11.6 Ma and a 1.5 Ma standart deviation	Prior: lognormal with and offset of 3.6 Ma and a 1.3 Ma standart deviation	

### 2.4 Climatic niche evolution in *Cinara*

For the following analyses, we pruned from the tree the species for which we had gathered fewer than five georeferenced occurrence records. The phylogenetic signal of each selected climate variable was estimated with the *λ* of Pagel (1999) on the time‐calibrated tree. *λ* varies between 0 (no phylogenetic signal, i.e., the character evolved independently of the phylogeny) and 1 (there is phylogenetic signal, i.e., the phylogeny fully represents the trait covariance among species). From the occurrence data, we calculated the median, minimum, and maximum values of each bioclimatic variable for each species or species group included in the tree and optimized the value of *λ* using the *phylosig* function of the *phytools* package in R (R Core Team, 2013; Revell, 2012). To test whether *λ* was significantly different from zero, we compared the observed likelihood value with the obtained likelihood value with a fixed *λ* of zero using a likelihood ratio test.

In order to describe the evolution of climate tolerance throughout the *Cinara* species' evolutionary history, we used the disparity through time (DTT) method (Harmon, Schulte, Larson, & Losos, 2003) that depicts how trait variation is distributed and when trait values diverged along the phylogeny. This method starts at the root of the tree and calculates the disparity among all existent clades up to the most recent node. The disparity at each node is the mean of the Euclidian distances for *n* dimensions (*n* being the number of variables) calculated for each pair of species of the clade. These disparity values are plotted against node ages. To compute disparities values, we extracted the median values for each bioclimatic variable for each species and used the *dtt* function in the *geiger* package (Harmon, Weir, Brock, Glor, & Challenger, 2007). Each variable was standardized so that each would have the same weight in the disparity calculation. To consider phylogenetic uncertainty, DTT values were estimated for 5,000 sampled trees from the BEAST posterior distribution inferred with a random starting tree. Low values of disparity suggest that species in the considered clades exhibit similar climatic niches, whereas high values mean that the species are adapted to different climates. Observed disparity values were compared with the values obtained under a pure Brownian process by simulating the evolution of climate variables 1,000 times across our tree using the covariance matrix with the *dtt* function. Loess regression curves were used to represent the observed and simulated disparities.

### 2.5 *Cinara* and *Pinus* niche equivalency tests

The clades B_2_, B_3_, and B_5_ of the *Cinara* tree (see below) encompass species feeding exclusively on pines (44 species in our dataset). We restricted our analyses of niche equivalency to these species because *Pinus*–*Cinara* associations are particularly well documented (Blackman & Eastop, 1994). In addition, occurrences available in public databases often specify the *Pinus* species whereas the identification of other *Cinara* hosts frequently does not reach the species level. We retrieved the association of each *Cinara* species with species of *Pinus* from Blackman and Eastop (2019) and from our own dataset. When *Cinara* species were clustered into species groups, the host niche represented the combination of the occurrences of all the hosts of all aphid species included in the group.

In order to test whether the realized climatic niches of *Cinara* species and their hosts were similar, we performed the niche equivalency tests described in Broennimann et al. (2012). For each *Cinara* species and for each host species range, we considered the environmental space as a rectangle with four sides defined as the minimum and maximum latitude and longitude where the species was found. To stay conservative in our analyses and try to encompass the whole environmental space, we broadened the rectangle by 10° in all directions. We considered the five bioclimatic variables described above and performed a principal component analysis (PCA) based on the climatic values extracted for each pixel of the environmental grid. Due to computational limitations, we extracted the climatic values for *Cinara* species and their respective host species and used a 5‐min grid resolution. We then created a grid with occurrence densities along the two PCA axes with the function *ecospat.grid.clim.dyn* of the *ecospat* package (Di Cola et al., 2017) at a resolution *R* = 100. This function uses a kernel density function to determine the density of occurrences in each cell: It ensures an unbiased estimation of niche overlap by dividing the number of times a species occurs in an environment by the frequency of the locations in the defined area that exhibits these environmental conditions. Broennimann et al. (2012) showed that this correction prevents underestimating niche overlap. Niches were then compared with Schoener's (1968) *D* statistic. The *ecospat.niche.equivalency.test* function pools all of the *Cinara* and host species occurrence data and randomly splits them into two datasets of the same size as the original datasets with which the niche overlap *D* is then calculated. This process is repeated 100 times and a histogram of the simulated values is constructed which allows a *p*‐value calculation: If the observed *D* falls into the density of 95% of the simulated values, the null hypothesis (i.e., the two niches are equivalent) cannot be rejected.

## 3 RESULTS

### 3.1 Occurrences and taxonomic validation

After filtering for duplicates and dubious locations, 1,756 of the 6,135 occurrences were retained. The COI tree revealed a few polyphyletic and paraphyletic species, suggesting these nominal species are regularly misidentified or harbor cryptic taxonomic entities (Appendix S1). Indeed, they represent well‐known cases of ambiguously defined species and represent taxonomic challenges in need of work (Appendix S2). This kind of phenomenon represents a possible source of error when using georeferenced data from unverified databases (e.g., GBIF) to plot on the tips of a phylogenetic tree. We defined 13 species groups that each encompassed closely related species that were regularly mixed in the COI tree and grouped occurrence data accordingly (Table 2). Each of these species groups gathered nominal taxa that have been the subject of taxonomic discussion and are often recognized as a single species when using species delimitation methods (Jousselin et al., 2013). In addition, we deleted all 31 occurrences that were assigned to *C. pinea* in China. Specimens identified as *C. pinea* in China were split into two distant genetic lineages (they either clustered with *C. pinea* from the rest of the world or appeared as the sister species of *C. atrotibialis*). This might be due to the use of alternative taxonomic keys for the eastern aphid fauna and implies that occurrences identified as *C. pinea* in China, with no COI data associated, cannot be attributed with certainty to the lineage identified as *C. pinea* throughout the rest of the Plaearctic (Appendix S1).

**Table 2:** Species groups

Group name	Concerned species	Group name	Concerned species
*Anelia*	*Cinara anelia*	*Nigra*	*Cinara canatra*
	*Cinara apini*		*Cinara gracilis*
	*Cinara hattori*		*Cinara nigra*
	*Cinara hirsuta*		
	*Cinara moketa*		
*Atlantica*	*Cinara atlantica*	*Obscura*	*Cinara obscura*
	*Cinara banksiana*		*Cinara pallidipes*
			*Cinara vandykei*
*Cembrae*	*Cinara cembrae*	*Pseudotsugae*	*Cinara pseudotsugae*
	*Cinara mongolica*		*Cinara splendens*
	*Cinara shinjii*		
*Confinis*	*Cinara confinis*	*Schwarzii*	*Cinara schwarzii*
	*Cinara grande*		*Cinara thatcheri*
	*Cinara longipennis*		
*Contortae*	*Cinara contortae*	*Mariana*	*Cinara braggii*
	*Cinara medispinosa*		*Cinara mariana*
	*Cinara murrayanae*		
*Cuneomaculata*	*Cinara cuneomaculata*	*Laricifoliae*	*Cinara laricifoliae*
	*Cinara laricicola*		*Cinara lyalli*
*Juniperi*	*Cinara juniperi*		
	*Cinara petersoni*		

### 3.2 Phylogeny and dating analyses

We obtained a 2,904 bp alignment (with 22% missing data). The phylogenies inferred with MrBayes and BEAST were well‐supported overall and consistent with the one estimated by Meseguer et al. (2015). Two main clades emerged (Appendix S3). Clade A is composed of species feeding on *Picea*, *Abies*, and Cupressaceae, whereas Clade B encompassed the bulk of the *Cinara* diversity. The species of Clade B_1_ are present on a wide range of hosts (i.e., *Abies*, *Pseudotsuga*, and *Larix*). The species of almost all the remaining Clades (B_2–5_) feed on *Pinus* with the exception of Clade B_4_, in which *C. wahluca* lives on Cupressaceae and other species feed on *Picea*. The positions of the B_1_ and B_2_ Clades differed in comparison with the tree obtained by Meseguer et al. (2015): Whereas they were recovered as sister clades by Meseguer et al. (2015), we estimated that B_2_ is more closely related to the clades B_3–5_. Support for these deep nodes is too low to draw a conclusion on the phylogenetic position of these clades.

The most recent common ancestor of all extant *Cinara* species was estimated to be 43.7 Ma old (33.58–66.61 Ma; Appendix S4), while the origin of Clade A is estimated at 30.41 Ma and that of Clade B at 30.85. These estimates are slightly younger than those of previous studies (Chen et al., 2016; Meseguer et al., 2015).

### 3.3 *Cinara* niche characterization and evolution

**Figure 1:**
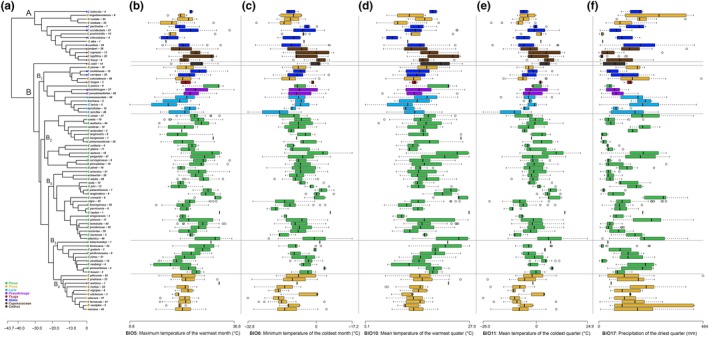
Present climatic niche of *Cinara* aphids represented in boxplots. (a) Phylogeny of the *Cinara* aphids inferred with BEAST 1.8.2. The scale is in millions of years. Tips are colored according to the host plants genus, and number of occurrences number are indicated in brackets. (b) Maximum temperature of the warmest month (BIO 5). (c) Minimum temperature of the coldest month (BIO 6). (d) Mean temperature of the warmest quarter (BIO 10). (e) Mean temperature of the coldest quarter (BIO 11). (f) Precipitation of the driest quarter (BIO 17)

After pruning the tree of species represented by fewer than five occurrences, the dataset included 67 species, with 5–93 occurrences each (mean = 26.9, *SD* = 20.2). Mapping the bioclimatic scores on the tips of the phylogenetic tree showed that none of the clades is restricted to specific climatic conditions (Figure 1). There is no phylogenetic clustering of climatic tolerance observed among the tips, with each species occupying a different subset of the climatic range of *Cinara* genus as a whole. The phylogenetic signal was low for four out of the five selected bioclimatic variables (Table 3). The exception was BIO 6 (minimum temperature of the coldest month) with a *λ* of 0.57, showing significant phylogenetic signal (*p* = .022). Similar results were obtained for the analysis based on the minimum or maximum values for each climate variable (Appendix S5).

**Table 3:** Pagel's lambda measured for the five bioclimatic variables with the phylosig function of the phytools package

Bioclimatic variable	*λ*	*p*‐value
BIO 5	0.26	.30
BIO 6	0.57	.022
BIO 10	6.9 × 10^–5^	1
BIO 11	0.45	.10
BIO 17	6.9 × 10^–5^	1

Note

The *p*‐value indicates if the lambda is significantly different from 0.

**Figure 2:**
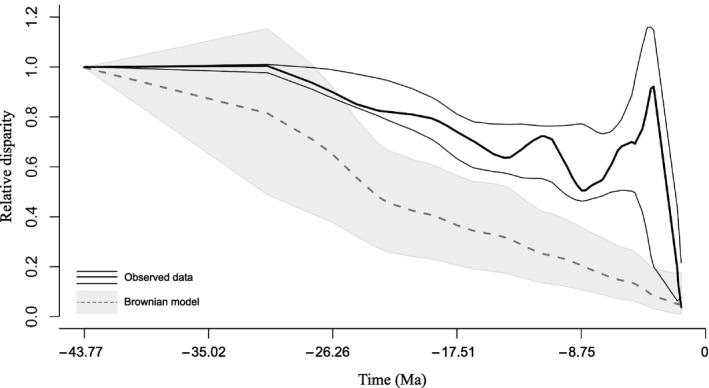
Disparity through time (DTT) plot for *Cinara* compared with expected disparity based on phylogenetic simulation. The solid black line represents the actual disparity calculated for *Cinara* over the maximum a posterior tree inferred with MrBayes and considering only the species represented by more than five occurrences. Thin black lines represent 2.5% and 97.5% disparity quantile of the 5,000 sampled BEAST trees. The dashed line represents the median of the simulated data under a Brownian model with the grey shadow indicating 2.5% and 97% quantiles

Many pairs of closely related *Cinara* species are adapted to different climatic niches. For example, *C. watsoni* occurs in warm regimes while its sister species, *C. pergandei*, occurs in colder conditions; *C. costata* diverged toward warmer conditions than *C. nimbata*; *C. harmonia* lives in much cooler conditions than *C. atlantica*. The pair of sister species *C. solitaria* and *C. glabra* occurs under drier environmental conditions than the closely related pair of species *C. watsoni* and *C. pergandei* (Figure 1). In agreement with these results, the DTT plot showed that the observed climatic niche disparity is greater than expected for a set of variables evolving under a Brownian motion throughout the phylogeny (Figure 2). The climatic niche diversity within the genus *Cinara* is thus represented in young clades and no clade seems specialized toward particular temperatures or precipitation conditions.

### 3.4 Niche equivalency tests

**Figure 3:**
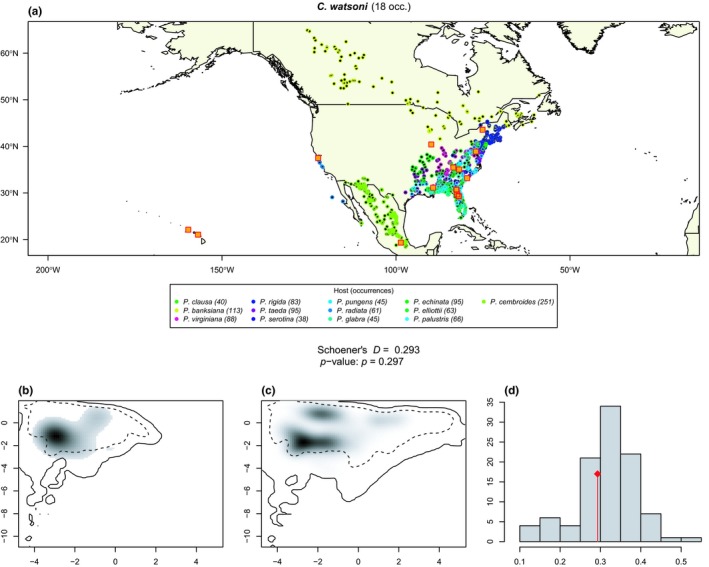
Climatic niche equivalency test for *Cinara watsoni* and its hosts (a) *C. watsoni* and *Pinus* occurrence distribution. *Cinara watsoni* 18 occurrences are represented by orange square. Host plants are indicated below the map and their corresponding occurrences are represented by colored circles. (b, c) Kernel grid based on the environmental PCA based on the available climatic conditions of the *Cinara* (b) and *Pinus* (c) occurrences. Gray shading shows the density of the occurrences of the species by cell. The solid and dashed contour lines illustrate, respectively, 100% and 50% of the available (background) environment. (d) Histogram of the Shoener's *D* values measured from the 100 randomizations. Observed *D* value in indicated by the red vertical line. The observed *D* value and the corresponding *p*‐value are indicated in the central square

**Figure 4:**
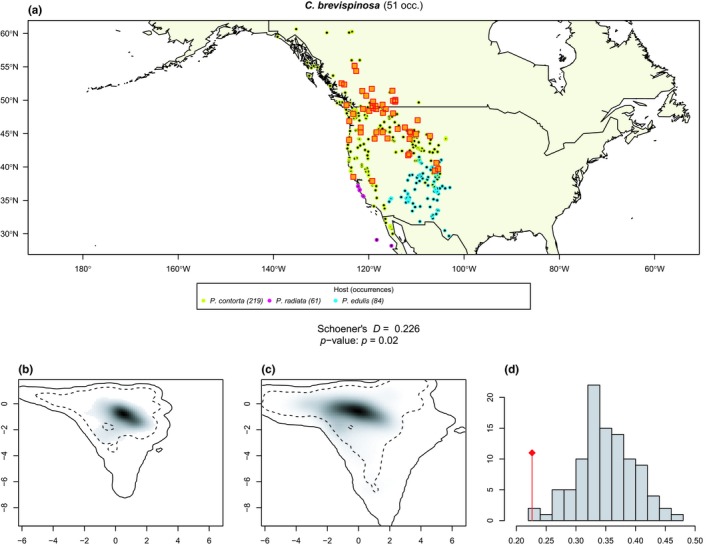
Climatic niche equivalency test for *Cinara brevispinosa* and its hosts (a) *C. brevispinosa* and *Pinus* occurrence distribution. *Cinara brevispinosa* 51 occurrences are represented by orange square. Host plants are indicated below the map, and their corresponding occurrences are represented by colored circles. (b, c) Kernel grid based on the environmental PCA based on the available climatic conditions of the *Cinara* (b) and *Pinus* (c) occurrences. Gray shading shows the density of the occurrences of the species by cell. The solid and dashed contour lines illustrate, respectively, 100% and 50% of the available (background) environment. (d) Histogram of the Shoener's *D* values measured from the 100 randomizations. Observed *D* value in indicated by the red vertical line. The observed *D* value and the corresponding *p*‐value are indicated in the central square

The geographic distribution of each aphid species and its range of host plants are presented as maps in Appendix S6. About two thirds (*n* = 24, Table 4) of our tests did not reject the null hypothesis of niche equivalency, suggesting that the *Cinara* species associated with *Pinus* occur under similar climatic conditions as their hosts. For instance, *C. watsoni*, one of the less‐specialized, oligophagous species of the genus, occurs under the same climatic conditions as its hosts (Figure 3). On the other hand, there are 10 *Cinara* species that exhibit a narrower climatic tolerance than their host plants (Table 4). This is the case, for example, of *C. brevispinosa* that is known to feed on *P. contorta*, *P. radiata*, and *P. edulis*: it occupies the colder part of the climatic niche of this range of hosts (Figure 4). These species that occupy only a portion of their host's climatic niche do not cluster together phylogenetically, suggesting that climate specialization is not a phylogenetically conserved trait. Specialist species (i.e., species feeding on 3 or fewer host plant species) tend to occur throughout the climatic niche of their hosts (only 3 of 15 specialist species rejected the null hypothesis of niche equivalency), whereas a consequent fraction the generalist species (feeding on 4 or more host plants) occupy only a restricted portion of their host plants climatic niche (7 of 19 generalist species rejected the null hypothesis of niche equivalency).

**Table 4:** Results of the climatic niche equivalency tests

	Schoener's *D* obs	Schoener's *D* *p*‐value	Occurrences number	Hosts number
Anelia	0.523	1	70	8
Atlantica	0.646	.762	47	13
*C. arizonica*	0.441	.059	21	3
*C. atrotibialis*	0.056	.01	16	4
*C. balachowskyi*	NA	NA	1	1
*C. brauni*	NA	NA	1	2
*C. brevispinosa*	0.135	.01	51	3
*C. bungeanae*	NA	NA	9	1
*C. cronartii*	0.347	1	6	5
*C. edulis*	0.216	.03	86	5
*C. formosana*	0.076	.02	23	7
*C. glabra*	0.250	.446	11	4
*C. gudaris*	NA	NA	2	1
*C. harmonia*	0.280	.475	5	1
*C. largirostris*	0.186	.822	6	3
*C. maghrebica*	0.057	.01	8	4
*C. neubergi*	NA	NA	4	1
*C. oregonensis*	NA	NA	3	2
*C. palaestinensis*	0.102	.069	7	2
*C. parvicornis*	0.062	.198	6	2
*C. pergandei*	0.179	.02	48	9
*C. pinea*	0.319	.733	42	4
*C. pini*	0.247	1	12	2
*C. piniarmandicola*	0.017	.01	25	1
*C. piniformosana*	0.016	.04	9	6
*C. pinimaritimae*	NA	NA	4	5
*C. pinivora*	0.133	.307	17	5
*C. ponderosae*	0.421	.02	52	8
*C. solitaria*	0.179	.436	5	1
*C. strobi*	0.596	.832	27	1
*C. taedae*	NA	NA	1	8
*C. terminalis*	0.249	.356	93	6
*C. wahtolca*	0.045	.089	44	5
*C. watanabei*	NA	NA	2	2
*C. watsoni*	0.386	.782	18	13
Cembrae	0.045	.05	10	3
Contortae	0.343	.228	36	1
Nigra	0.210	.089	43	3
Nuda	0.054	.485	10	2
*S. curvispinosus*	0.389	.525	9	1
*S. pineti*	0.045	.01	19	8
*S. piniradiatae*	0.186	.446	20	9
Schwartzii	0.297	.149	20	6

Note

The tests were performed only for pine‐feeding *Cinara* represented by at least five occurrences. Gray highlighted species are the ones for which the test is significant.

## 4 DISCUSSION

Climatic niches have been shown to be phylogenetically constrained in many organisms, including endotherms (Araújo et al., 2013; Ortega‐García et al., 2017), ectotherms (Grigg & Buckley, 2013; Hutter, Guayasamin, & Wiens, 2013; Pyron & Burbrink, 2009), and plants (Boucher et al., 2012; Grossenbacher, Veloz, & Sexton, 2014; Schnitzler, Graham, Dormann, Schiffers, & Peter Linder, 2012). Although they represent a substantial portion of terrestrial biodiversity, little is known about the phylogenetic patterns of climatic niche evolution in insects over long evolutionary time scales (but see Hidalgo‐Galiana, Sánchez‐Fernández, Bilton, Cieslak, & Ribera, 2014; Kellermann et al., 2012) and this is especially true for phytophagous insects. The relative lack of phytophagous insect studies is due in part to the limited knowledge of the insects' distribution and host association, but also because their host specialization confounds accurate niche estimation (see below). In order to understand the relative role of climatic niche and the evolution of host preference evolution on phytophagous insect history, a species‐level association matrix is needed. These matrices are particularly hard to compile for insects that do not feed on their hosts as adults. Aphids feed on their hosts during their entire life and are thus good models for studying plant‐insect associations.

Using an occurrence dataset and a robust phylogeny, we show that climatic niche tolerance is evolutionarily labile in the aphid genus *Cinara*, with the most of the analyzed temperature and precipitation climate variables displaying a weak phylogenetic signal (Table 3). These results suggest that none of the *Cinara* clades appeared specialized for any specific climate conditions. On the contrary, all clades encompassed most of the climatic niche variation described within the genus and the observed disparity values for closely related species are greater than simulated under neutral evolution (Figures 1 and 2). These results may indicate a rapid evolution of climate tolerance in the genus and suggest that climatic niche divergence, and adaptation may have played an important role in its diversification and distribution. Alternatively, the climatic niche plasticity observed in our study might simply be the result of the aphids' associations with hosts occurring in disparate climatic conditions. Under this latter hypothesis, the realized climatic niche of the specialized insect may actually be narrower than its fundamental niche (i.e., the set of climatically suitable conditions without the constraints of biotic components) and determined by the availability of suitable host plants rather than by the aphid's own physiological limits (Soberon & Peterson, 2005). Although correlative approaches based on distribution data cannot indicate whether host‐specialized insects have a wider niche than their hosts, they can reveal situations where insects occupy narrower niches.

Our niche equivalency tests found that a majority of the *Cinara* species included in this study exhibited a realized climatic niche equivalent to that of their hosts (Table 4), thereby corroborating the hypothesis that the main constraint underlying *Cinara* species' distribution is the identity of their hosts. This is in line with the results of Kanturski, Bugaj‐Nawrocka, and Wieczorek (2016) that modeled the climatic niche of three pine associated aphids (from the genus *Eulachnus*) and showed that the use of biotic layers, in the form of potentially suitable niche space for host plants of each species of aphid, did not produce significantly different models for aphids. Nonetheless, 10 *Cinara* species (29% of our sampled taxa) exhibited a different pattern: They displayed a climatic niche narrower than that of their hosts. Specialist species generally demonstrate niche equivalency with their hosts (3/15) whereas the ratio of climatically constrained species in generalists (7/19) is relatively high. This result suggests that diet breath does not necessarily correlate with climatic niche breadth and that physiological limits can constrain aphid distribution. Alternatively, the lower ratio of specialists rejecting the null hypothesis might stem from a generally lower number of occurrences for specialist species (mean for specialist 15.4, mean for generalist 34.2) which might limit the power of the niche equivalency test. Although the niche equivalency test we used is supposed to be robust to small sample sizes, it is mainly designed to limit type I error (i.e., incorrectly rejecting niche equivalency, Broennimann et al., 2012). This may also be explained by the specialists' trend to have a more limited available space. In our study, all the species with climate tolerance narrower than their host's occupied the colder range of their host's climatic niche and were absent from warmer regions. It has long been noted that aphids are negatively impacted by heat and drought stress (Hazell, Pedersen, Worland, Blackburn, & Bale, 2008). Heat stress has been shown to impact aphid's development and mortality (Ma, Hau, & Poehling, 2004; Montllor, Maxmen, & Purcell, 2002; Nguyen, Michaud, & Cloutier, 2009), and seasonal changes in photoperiod and temperature impact the induction of sexual reproduction and the production of frost‐resistant eggs (Trionnaire, Hardie, Jaubert‐Possamai, Simon, & Tagu, 2008). Studies have also shown that aphid population dynamics have been affected by recent global changes, possibly due to temperature increases (Hullé, Coeur d'Acier, Bankhead‐Dronnet, & Harrington, 2010), with global aphid diversity and abundance decreasing with increasing temperatures (Dixon, 1987). In addition, other indirect effects of temperature could explain this inverse latitudinal diversity gradient, for example, a lower efficiency of host plant location in warm environments with greater diversity but lower specific abundance of plants, or the presence of more competitors under warmer environments (Dixon, 1987). Alternatively, the narrow range of climatic tolerance in some *Cinara* species may be due to historical barriers limiting their expansion throughout the full geographic range of their hosts (Meseguer et al., 2015). This explanation does not seem to fit our dataset; however, as species exhibiting a narrower niche than their hosts do not show any obvious geographic clustering on the maps and are scattered throughout the geographic range of their hosts, occupying its coldest parts, for example mountain ranges (Figures 3 and 4 and Appendix S6).

We cannot ignore the possibility that the climatic differences detected in the niche equivalency tests are the result of an incomplete characterization of the aphids' geographic ranges. Although we gathered a relatively large occurrence dataset (see Boucher et al., 2012; Burbrink & Pyron, 2010; Nürk, Uribe‐Convers, Gehrke, Tank, & Blattner, 2015 for studies on vertebrates an plants with a similar number of occurrences per species), some *Cinara* species were represented by relatively few occurrences; for example, there were only five occurrences for *C. solitaria* and *C. harmonia*. These species, however, are only known from very restricted areas, suggesting that the small number of occurrences is not a sampling artifact but probably indicative of their true distribution. In any case, species that were represented by few occurrences (fewer than 10) always failed to reject the equivalency test. Hence, undersampling did not to generate type I errors (i.e., claiming that aphids live in a subset of their hosts niche because of an inaccurate sampling). We nevertheless noted several cases where sampling did seem incomplete: That is, cases where there was no overlapping distribution between *Cinara* species and at least one of their host plant species,for example, *P. banksiana*–*C. watsoni* (Figure 3, *C. watsoni*) failed to reject the null hypothesis, so again we do not think that undersampling led to a false positive in this case. Finally, the sampling of *Cinara* species is relatively uniform within their known geographic range with no obvious biases toward particular areas and a map superimposing all sampling points shows that a large geographic range has been covered for the entire group. We emphasize also that when *Cinara* species were sampled (at least by the authors of this study) the aphids were sampled broadly on many conifer hosts, with no focus on any particular species in any particular locations. Therefore, the absence of a particular species in a well‐covered area in our dataset, such as Southern California, is probably not a sampling artifact. The broad sampling reported here should largely obviate possible underestimation of the range of climate conditions under which these species occur.

As a perspective, we must keep in mind that our analysis focused on broad climatic conditions at macroevolutionary scales and did not take into account microhabitat conditions. Future studies could focus on other parameters that are important for aphids. For example, some species migrate from branches to tree roots during summer heat (Durak, 2014; Struble, Osgood, & Pepper, 1976). Hence, different species that occupy the same broad climatic regimes may also experience different microclimatic conditions permitting the coexistence of species on the same host (Favret & Voegtlin, 2004). The natural enemies and competitors encountered by aphids might also drive biogeographic patterns at regional scales. A combined characterization of aphid tolerance at the macro‐ and microclimatic scales could provide a more complete characterization of the interactions of the insects, their host plants, and the climate they experience.

## 5 CONCLUSIONS

The realized climatic niche of a phytophagous insect is necessarily associated with its host plant distribution. It is therefore likely that occurrence data for these insects represent incomplete characterizations of their potential ranges and climatic niches that can bias inferences of niche evolution at geological time scales (Saupe et al., 2017). Here, we show that most of the *Cinara* species investigated occupy a climatic niche equivalent to that of their hosts, suggesting that the main constraint underlying *Cinara* species' distributions is the physical presence of their hosts. However, we also detected several species that occupy a narrower climatic niche than their hosts, showing a preference for colder conditions. These results suggest that even in host‐specialized herbivorous insects, the niche cannot always be fully described in terms of an insect's host association and that adaptation to abiotic factors should be taken into account.

Our results also have bearing on discussing the future of biodiversity in the context of current climate change. We are witness to a dramatic increase in atmospheric CO_2_ concentration due to human activities and a concomitant increase in global mean temperature (Waters et al., 2016). The speed at which climate is changing today will substantially alter the global distribution of species over the course of this century (WWF, 2016). Different insect lineage have been already found to track cooler habitats in response to climate warming (Paradis et al., 2008; Parmesan, 2006). The fact that many aphid species cannot track their hosts throughout the warmer areas of their geographic distribution suggests they risk being affected by climate warming. This will most probably induce population isolation, especially in mountain regions where aphids are diverse (Huang, Lei, & Qiao, 2008; Palmer, 1952). The geographic distributions some of these aphid species will probably move northward, increasing the potential threat they can represent for their host trees in these regions.

## CONFLICT OF INTEREST

The authors declare that they have no conflict of interest. This article does not contain any studies with human participants or animals performed by any of the authors.

## AUTHOR CONTRIBUTIONS

PA, EJ, ASM, and ACA designed the study. PA and ASM performed the analyses with contributions from MG. ACA, EJ, CF, and Qiao G.X. provided the occurrence data. PA, EJ, and ASM wrote the paper with contributions from ACA, CF, and MG.

## Data Availability

Sampling locations, *Cinara*/*Pinus* association matrix, accession numbers and dated tree are available on Dryad: https://doi.org/10.5061/dryad.1r7j2r7.
