# Supplementary material for: The evolution of climate tolerance in conifer‐feeding aphids in relation to their host's climatic niche
Source: Ecol Evol. 2019 Oct 2;9(20):11657–71. doi: 10.1002/ece3.5652 (PMC6822038; doi:10.1002/ece3.5652)
Supplement: Supplementary file 1 [file ECE3-9-11657-s001.pdf]

Appendix 1 - NJ tree obtained from 743 COI barcodes from Cinara spp. The tree leaves indicate species names and genbank sequence number when the sequence was obtained from Bold or Genbank. Species groups are indicated by a bracket on the figure. On the right inside from right to left, are indicated: the origin of the sequence is indicated (Bold, CBGP, Genbank, GenB_Bold=in Bold but mined from Genbank), the geographic origin of the specimen when available (Province or State, followed by Country). "Occurrencetotal" (Yes or No) indicates whether the specimen was present in the occurence dataset, "Occurence Cleaned" (Yes or No) indicates whether the specimen can be found in the final occurence dataset once cured from redundant occurences or taxonomic amibiguities.

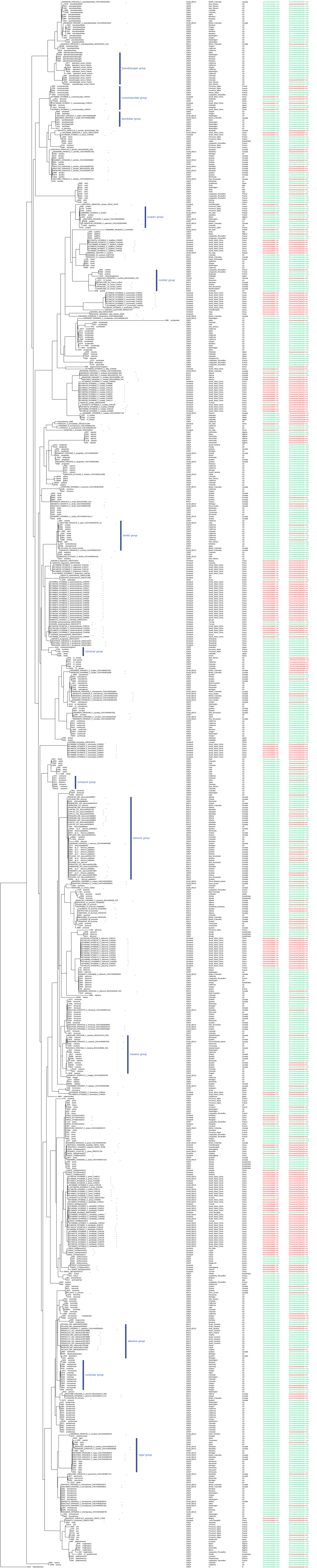

'pseudotsugae' group
'cuneomaculata' group
'laricifoliae' group
'juniperi' group
'confinis' group
'anelia' group
'cembrae' group
'schwarzii' group
'obscura' group
'mariana' group
'atlantica' group
'contortae' group
'nigra' group
