# Supplementary material for: The evolution of climate tolerance in conifer‐feeding aphids in relation to their host's climatic niche
Source: Ecol Evol. 2019 Oct 2;9(20):11657–71. doi: 10.1002/ece3.5652 (PMC6822038; doi:10.1002/ece3.5652)
Supplement: Supplementary file 2 [file ECE3-9-11657-s002.pdf]

Appendix 2 - Species group definiton

The group formed by *C. murrayanae*, *C.contortae* and *C. medispinosa*, despite intensive morphometric studies (Footit 1987), are still reported in 2019 as requiring further investigation (Blackman and Eastop 2019) ; *C. lyalii* has been reported in the literature as possibly being a form of *C. laricifoliae* (Eastop 1972); *C. laricicola* may be a synonym of *C. cuneomaculata* (Blackman and Eastop 2017); Remaudière and Remaudière (1997) placed *C. splendens* as a possible synonym of *C. pseudotsugae* and *C. thatcheri* as probable synonym of *C. schwarzii*; they also considered *C. braggii* and *C. mariana* as a synonym of a third species, *C. glehna*, that is absent in our COI dataset; according to Blackman and Eastop (1994), *C. pallidipes* could be a synonym of *C. obscura*; inside the anelia group, *C. kuchea* has been considered as a possible synonym of *C. hirsuta* (Blackman and Eastop 1994); frequent misidentification has been recognized for East Asian specimens of *C. mongolica* that are erroneously assigned to it sister species *C. cembrae* (Blackman and Eastop 2019); *C. petersoni* is also reported as very similar to *C. juniperi* with only slight difference in length of the fourth segment of antenna and color of living specimen (Bradley 1963) which could reflect phenotypic plasticity. Because they are synonyms (Voegtlin 1976), we assigned *C. taxifoliae* and *C. escherichi* occurrences with the *pseudotsugae* group and *nuda* group, respectively.

Blackman RL, Eastop VF (1994) Aphids on the world's trees: an identification and information guide. Cab International

Blackman RL, Eastop VF (2019) Aphids on the World's Plants. An online identification and information guide. www.aphidsonworldsplants.info. Accessed on Janvier 2019.

Bradley G (1963) Two new species of Cinara Curtis (Homoptera: Aphididae) from Juniperus horizontalis Moench. The Canadian Entomologist

Eastop VF (1972) A taxonomic review of the species of Cinara Curtis occurring in Britain (Hemiptera: Aphididae). Bulletin of the British Museum (Natural History), Entomology 27

Footit RG (1987) Morphometric analysis of character variation and taxonomic discrimination among a complex of species of the genus Cinara (Homoptera: Aphidoidea: Lachnidae), Theses (Dept. of Biological Sciences)/Simon Fraser University

Remaudière G, Remaudière M (1997) Catalogue des Aphididae du monde (Homoptera Aphidoidea): Catalogue of the world's Aphididae. Editions Quae

Voegtlin DJ (1976) A biosystematic study of Cinara spp.(Homoptera: Aphididae) of the conifers of the westside Sierra forests. University of California
