# Supplementary material for: The evolution of climate tolerance in conifer‐feeding aphids in relation to their host's climatic niche
Source: Ecol Evol. 2019 Oct 2;9(20):11657–71. doi: 10.1002/ece3.5652 (PMC6822038; doi:10.1002/ece3.5652)
Supplement: Supplementary file 4 [file ECE3-9-11657-s004.pdf]

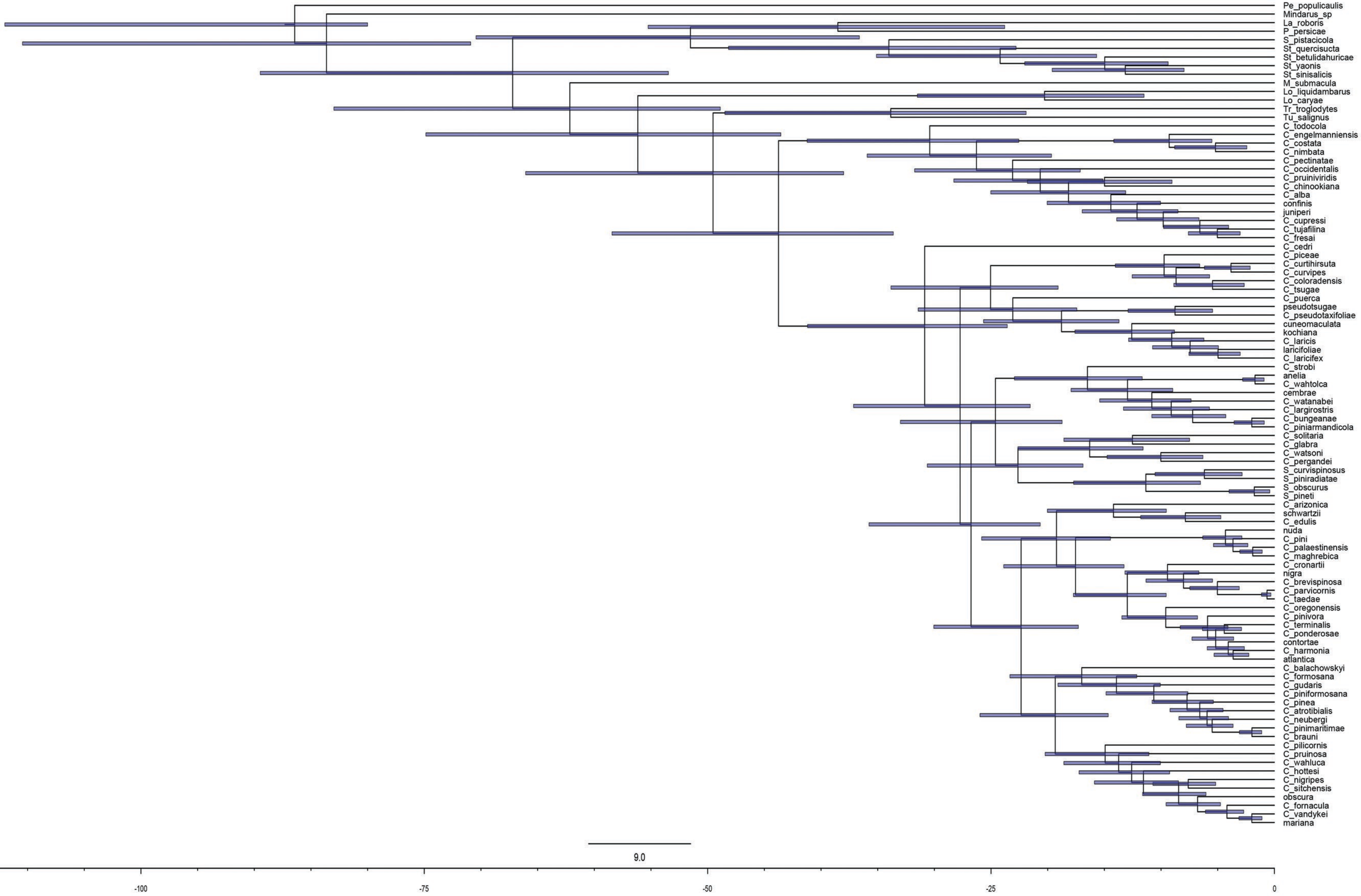

Appendix 4: Output of BEAST analyses with the MrBayes 3.2.3 topology fixed. Node bars indicate 95% highest posterior density intervals. The scale is in millions of years.
