# Supplementary material for: The evolution of climate tolerance in conifer‐feeding aphids in relation to their host's climatic niche
Source: Ecol Evol. 2019 Oct 2;9(20):11657–71. doi: 10.1002/ece3.5652 (PMC6822038; doi:10.1002/ece3.5652)
Supplement: Supplementary file 5 [file ECE3-9-11657-s005.pdf]

Appendix 5 *: Pagel's lambda measured for the five min and max bioclimatic variables with the phylosig function of the phytools package.The P value indicate if the lambda is significantly different from 0.*

Bioclimatic variable	lambda	P value
Minimum		
BIO 5	*0.30*	*0.10*
BIO 6	*0.64*	*0.024*
BIO 10	*0.35*	*0.10*
BIO 11	*0.50*	*0.12*
BIO 17	6.6×10^{-5}	*1*
Maximum		
BIO 5	6.7×10^{-5}	*1*
BIO 6	6.4×10^{-5}	*1*
BIO 10	*0.34*	*1*
BIO 11	*0.17*	*0.10*
BIO 17	6.6×10^{-5}	*1*
