# Supplementary material for: The evolution of climate tolerance in conifer‐feeding aphids in relation to their host's climatic niche
Source: Ecol Evol. 2019 Oct 2;9(20):11657–71. doi: 10.1002/ece3.5652 (PMC6822038; doi:10.1002/ece3.5652)
Supplement: Supplementary file 6 [file ECE3-9-11657-s006.pdf]

Appendix 6 - Climatic niche equivalency test for *Cinara* and its hosts (A) *Cinara* and *Pinus* occurrence distribution. *Cinara* occurrences are represented by orange square. Host plants are indicated below the map and their corresponding occurrences are represented by colored circles. (B and C) Kernel grid based on the environmental PCA based on the available climatic conditions of the *Cinara* (B) and *Pinus* (C) occurrences. Grey shading shows the density of the occurrences of the species by cell. The solid and dashed contour lines illustrate, respectively, 100% and 50% of the available (background) environment. (D) Histogram of the Shoener's D values measured from the 100 randomizations. Observed D value in indicated by the red vertical line. The observed D value and the corresponding p-value are indicated in the central square.

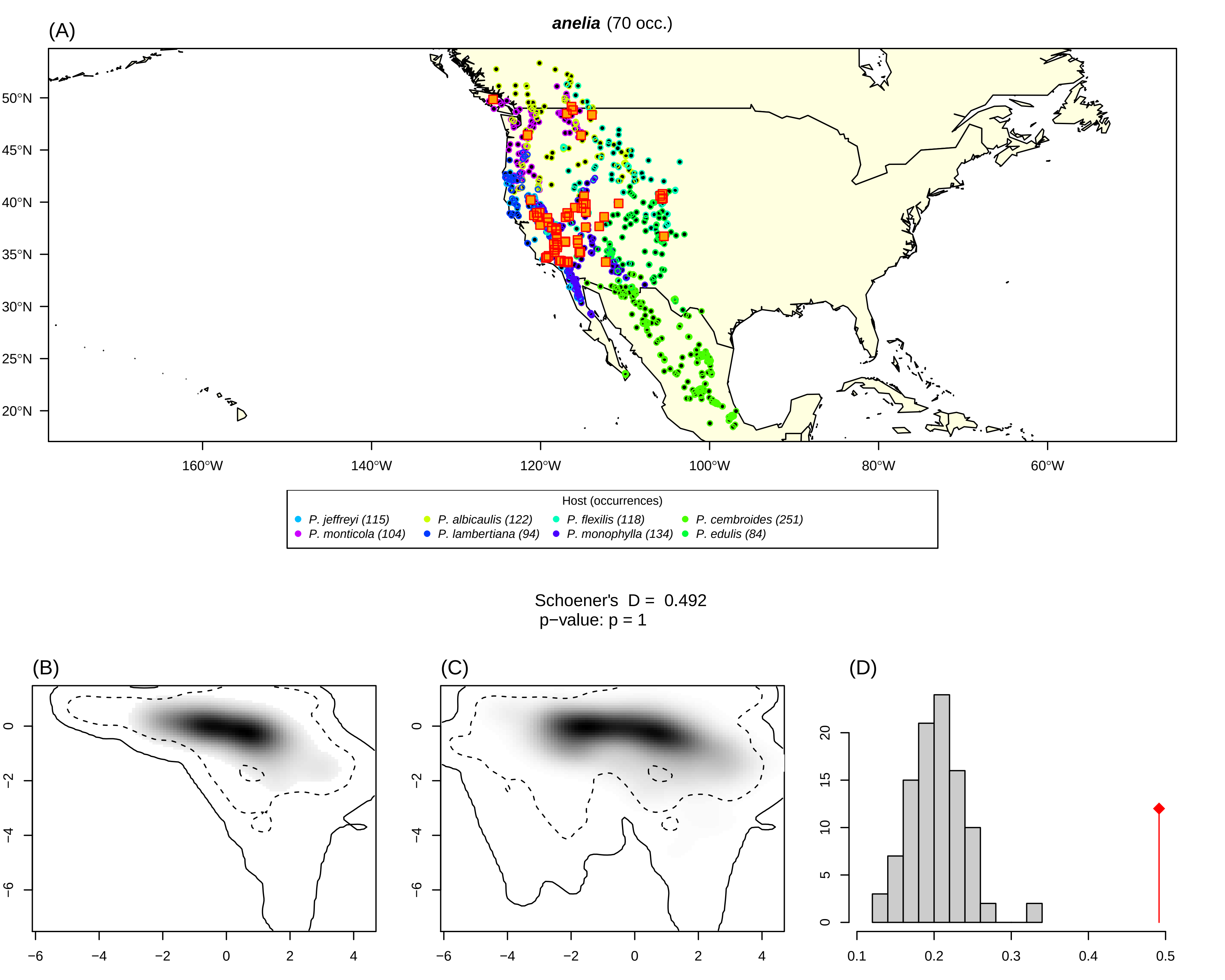
(A)
anelia (70 occ.)
50°N
45°N
40°N
35°N
30°N
25°N
20°N
160°W
140°W
120°W
100°W
80°W
60°W
Host (occurrences)
P. jeffreyi (115)
P. monticola (104)
P. albicaulis (122)
P. lambertiana (94)
P. flexilis (118)
P. monophylla (134)
P. cembroides (251)
P. edulis (84)
Schoener's D = 0.492
p−value: p = 1
(B)
(C)
(D)
0
−2
−4
−6
4
2
0.1
0.2
0.3
0.4
0.5
5
10
15
20

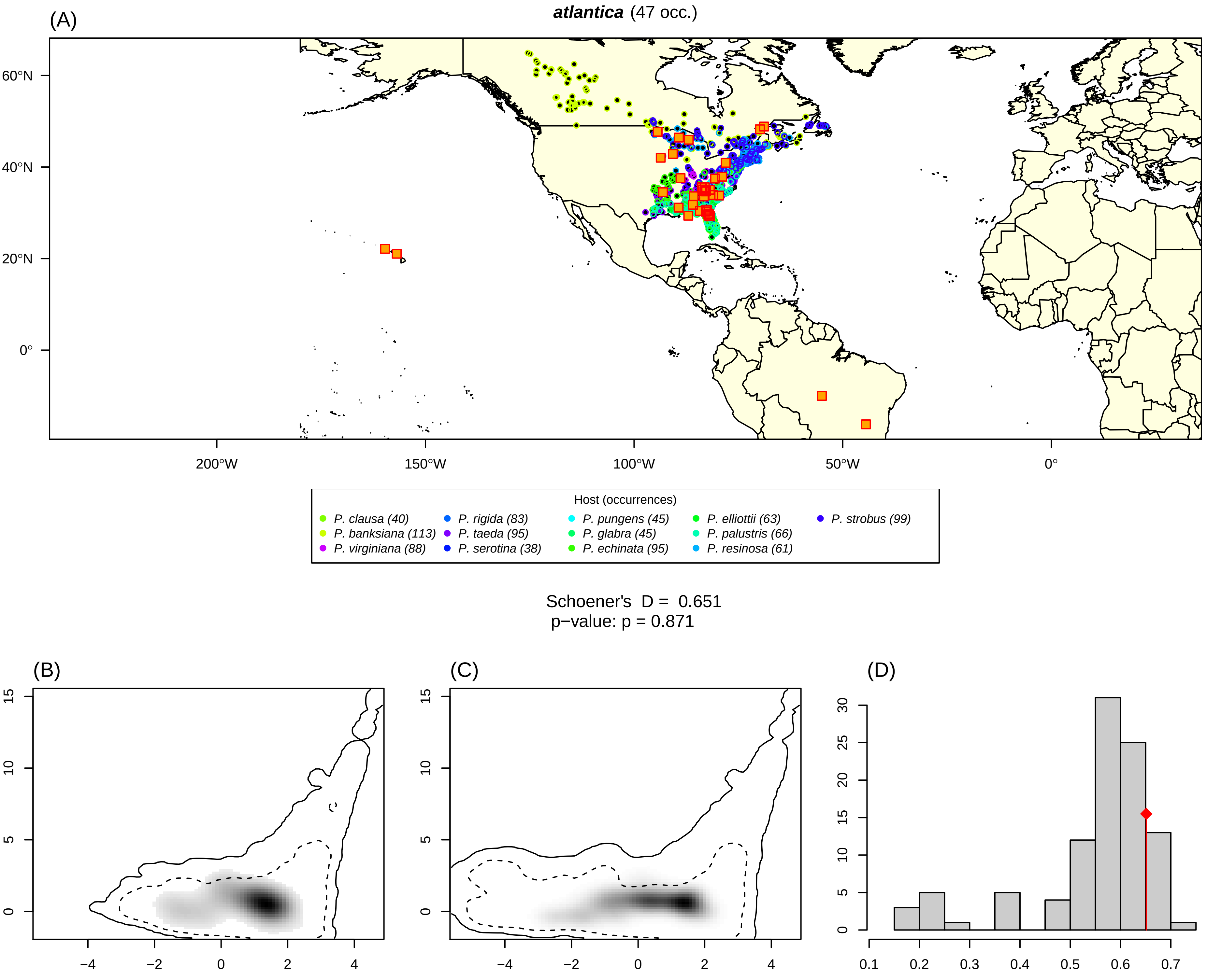

(A)
atlantica (47 occ.)
60°N
40°N
20°N
0°
200°W
150°W
100°W
50°W
0°
Host (occurrences)
P. clausa (40)
P. banksiana (113)
P. virginiana (88)
P. rigida (83)
P. taeda (95)
P. serotina (38)
P. pungens (45)
P. glabra (45)
P. echinata (95)
P. elliottii (63)
P. palustris (66)
P. resinosa (61)
P. strobus (99)
Schoener's D = 0.651
p−value: p = 0.871
(B)
(C)
(D)

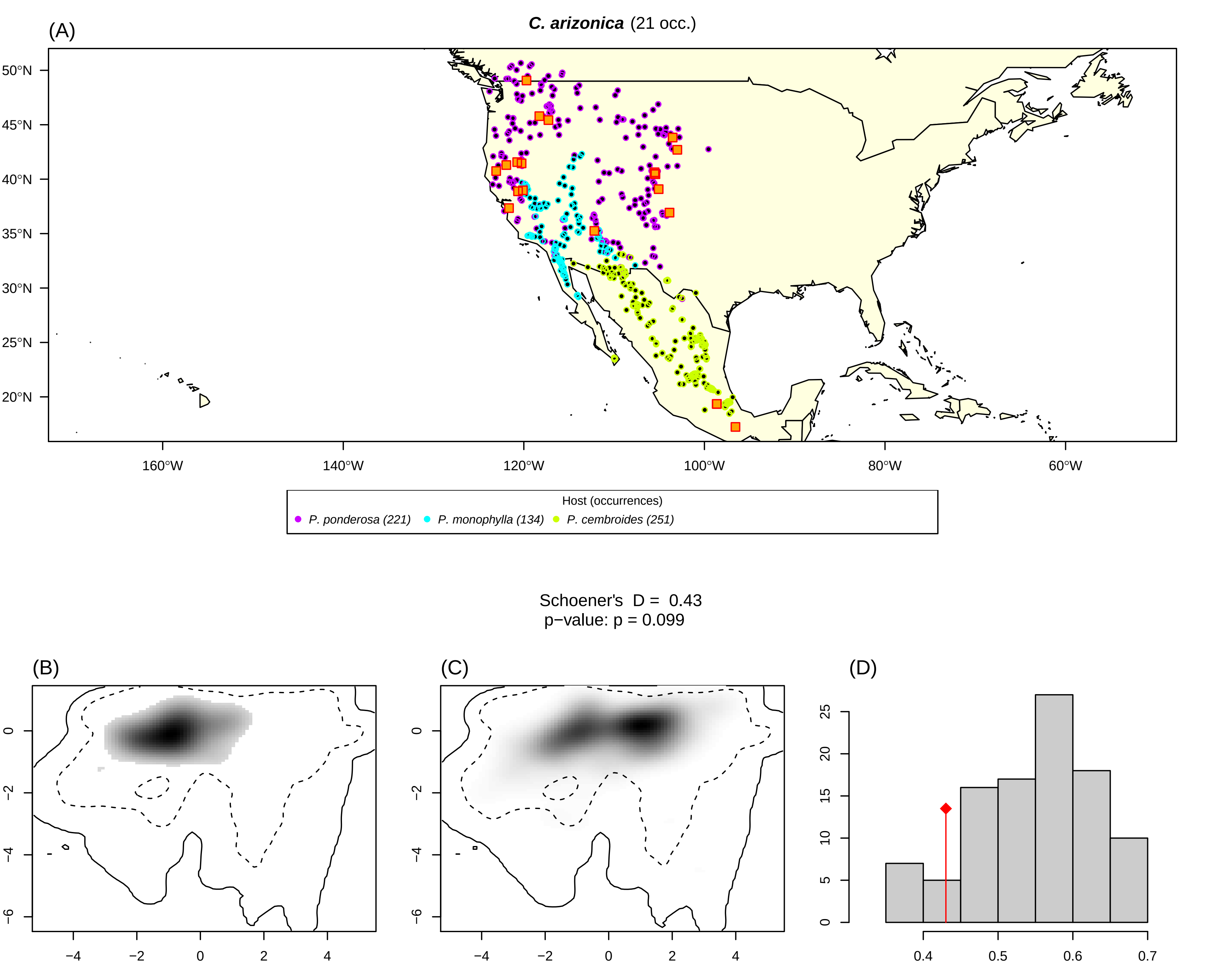
(A)
C. arizonica (21 occ.)
50°N
45°N
40°N
35°N
30°N
25°N
20°N
160°W
140°W
120°W
100°W
80°W
60°W
Host (occurrences)
P. ponderosa (221)
P. monophylla (134)
P. cembroides (251)
Schoener's D = 0.43
p−value: p = 0.099
(B)
(C)
(D)
0
−2
−4
−6
−4
−2
0
2
4
0
5
10
15
20
25
0.4
0.5
0.6
0.7

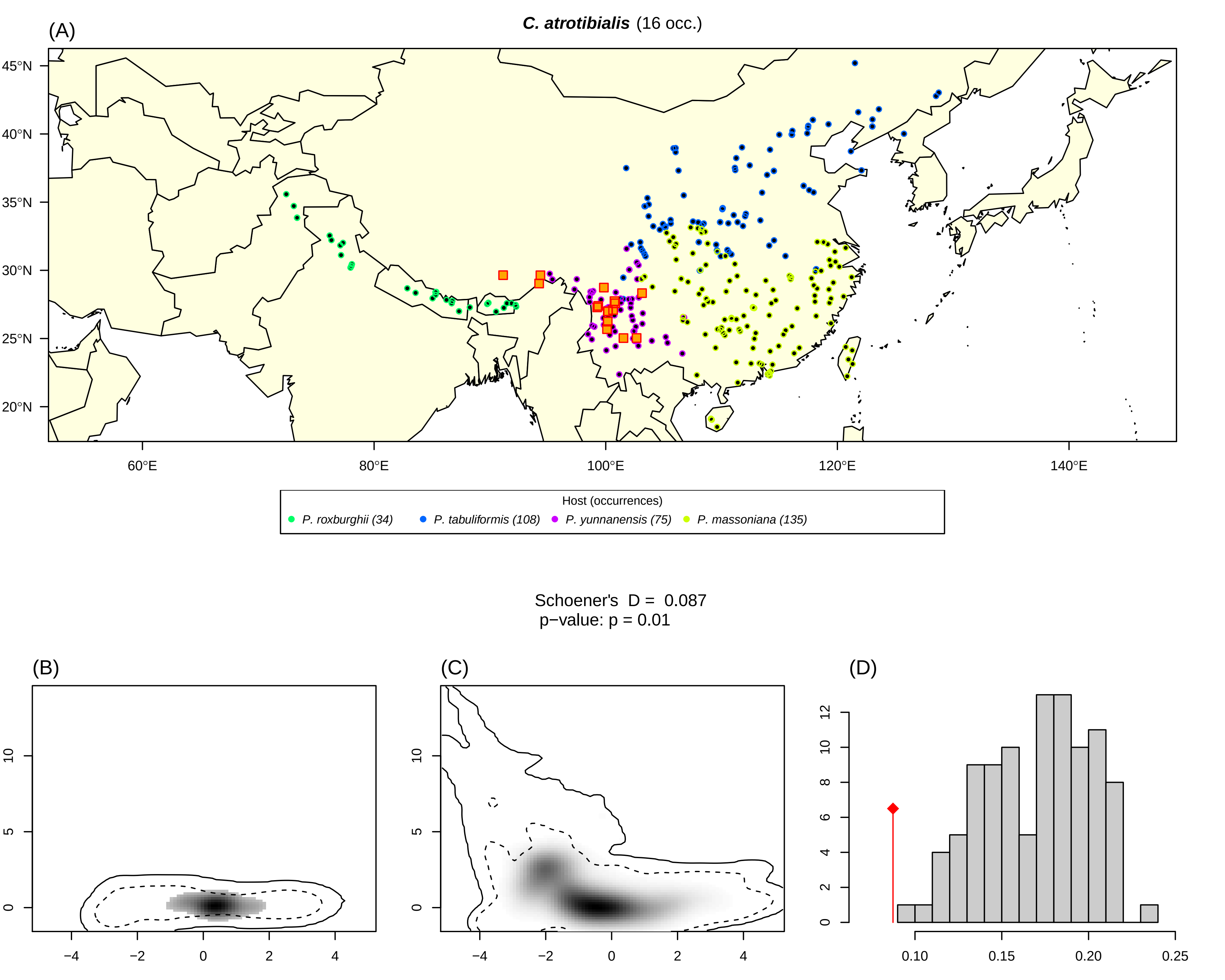

C. atrotibialis (16 occ.)
(A)
45°N
40°N
35°N
30°N
25°N
20°N
60°E
80°E
100°E
120°E
140°E
Host (occurrences)
P. roxburghii (34)
P. tabuliformis (108)
P. yunnanensis (75)
P. massoniana (135)
Schoener's D = 0.087
p−value: p = 0.01
(B)
(C)
(D)
10
5
0
−4
−2
0
2
4
12
10
8
6
4
2
0
0.10
0.15
0.20
0.25

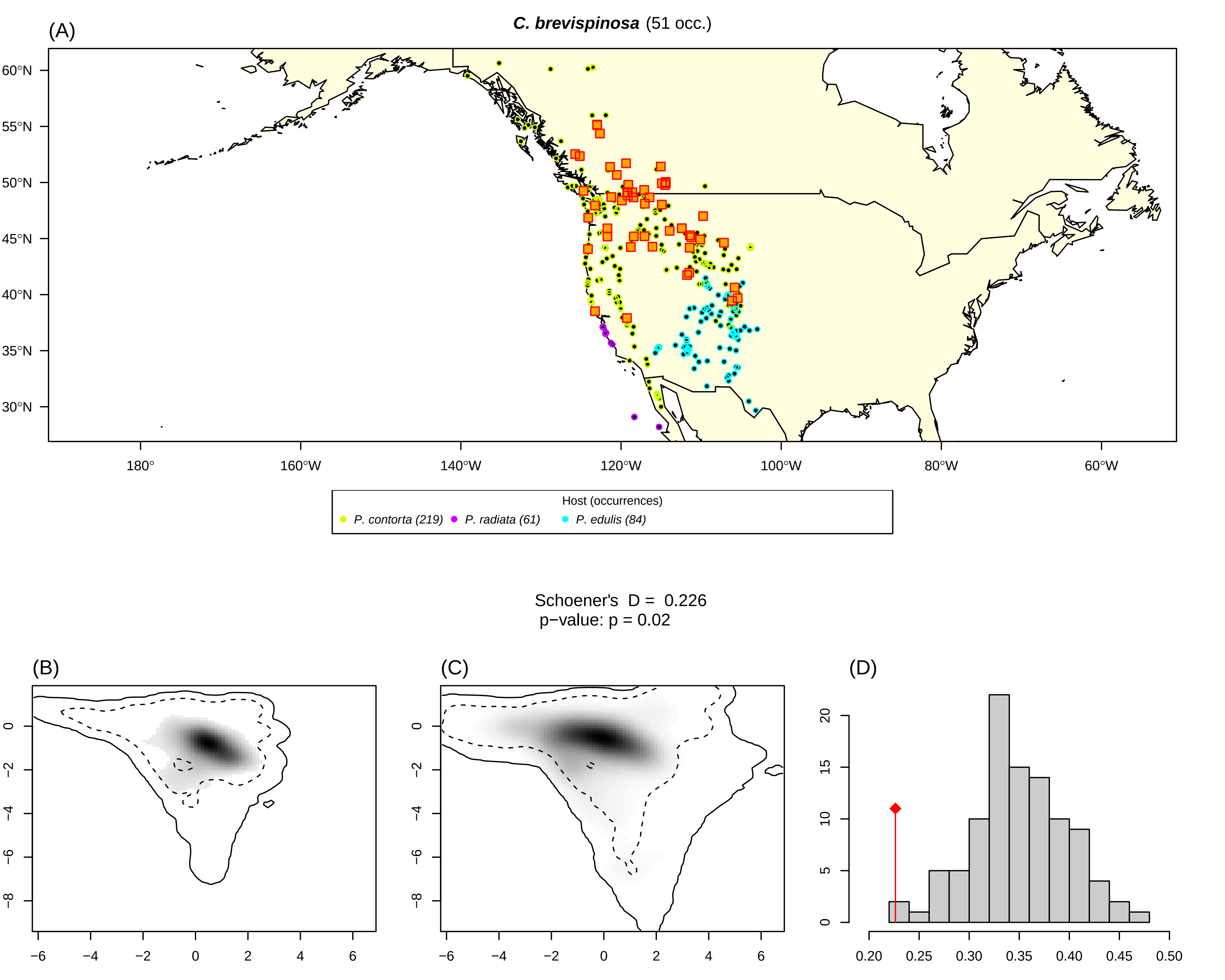

(A)
C. brevispinosa (51 occ.)
60°N
55°N
50°N
45°N
40°N
35°N
30°N
180°
160°W
140°W
120°W
100°W
80°W
60°W
Host (occurrences)
P. contorta (219)
P. radiata (61)
P. edulis (84)
Schoener's D = 0.226
p−value: p = 0.02
(B)
(C)
(D)

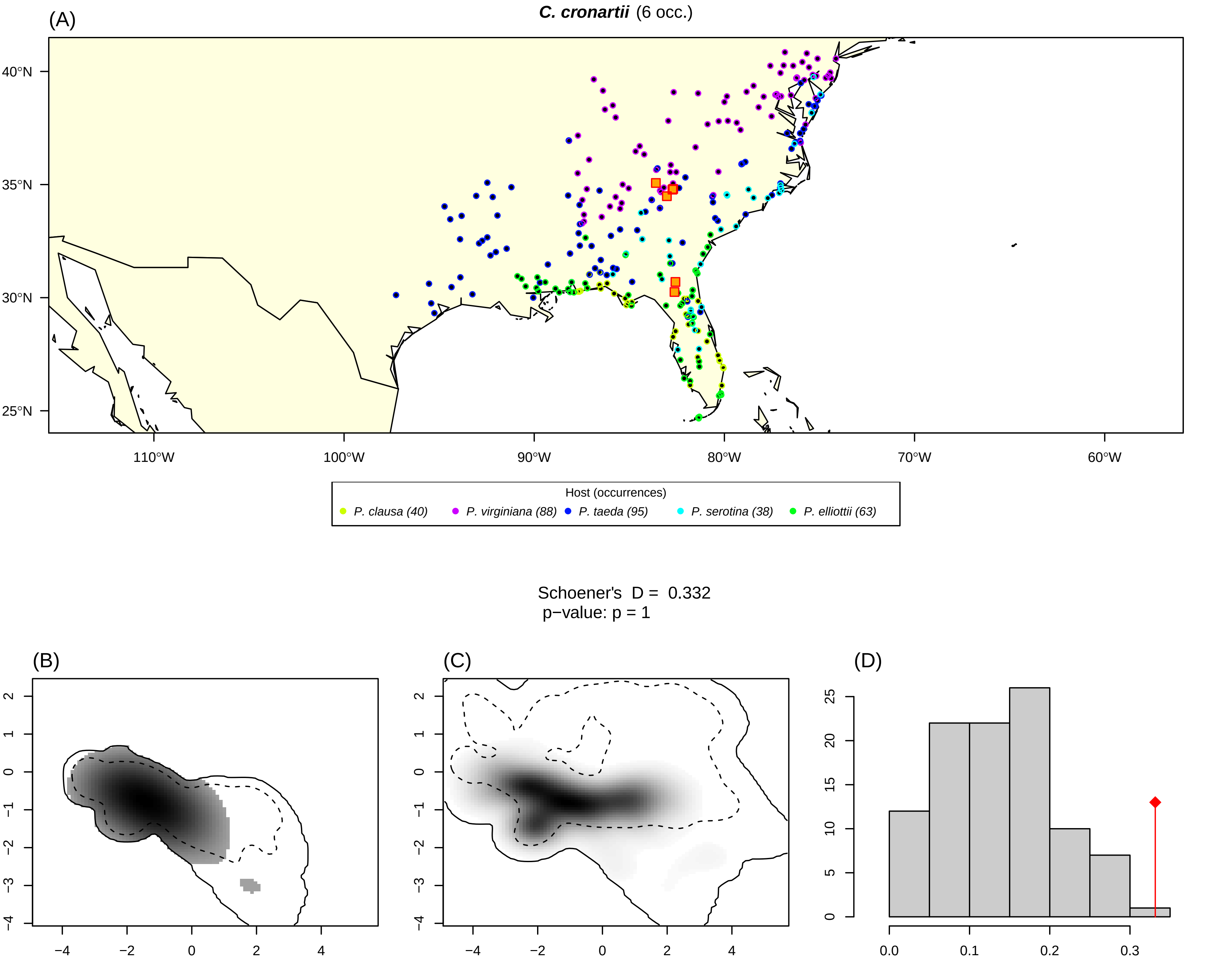

C. cronartii (6 occ.)
(A)
40°N
35°N
30°N
25°N
110°W
100°W
90°W
80°W
70°W
60°W
Host (occurrences)
P. clausa (40)
P. virginiana (88)
P. taeda (95)
P. serotina (38)
P. elliottii (63)
Schoener's D = 0.332
p−value: p = 1
(B)
(C)
(D)

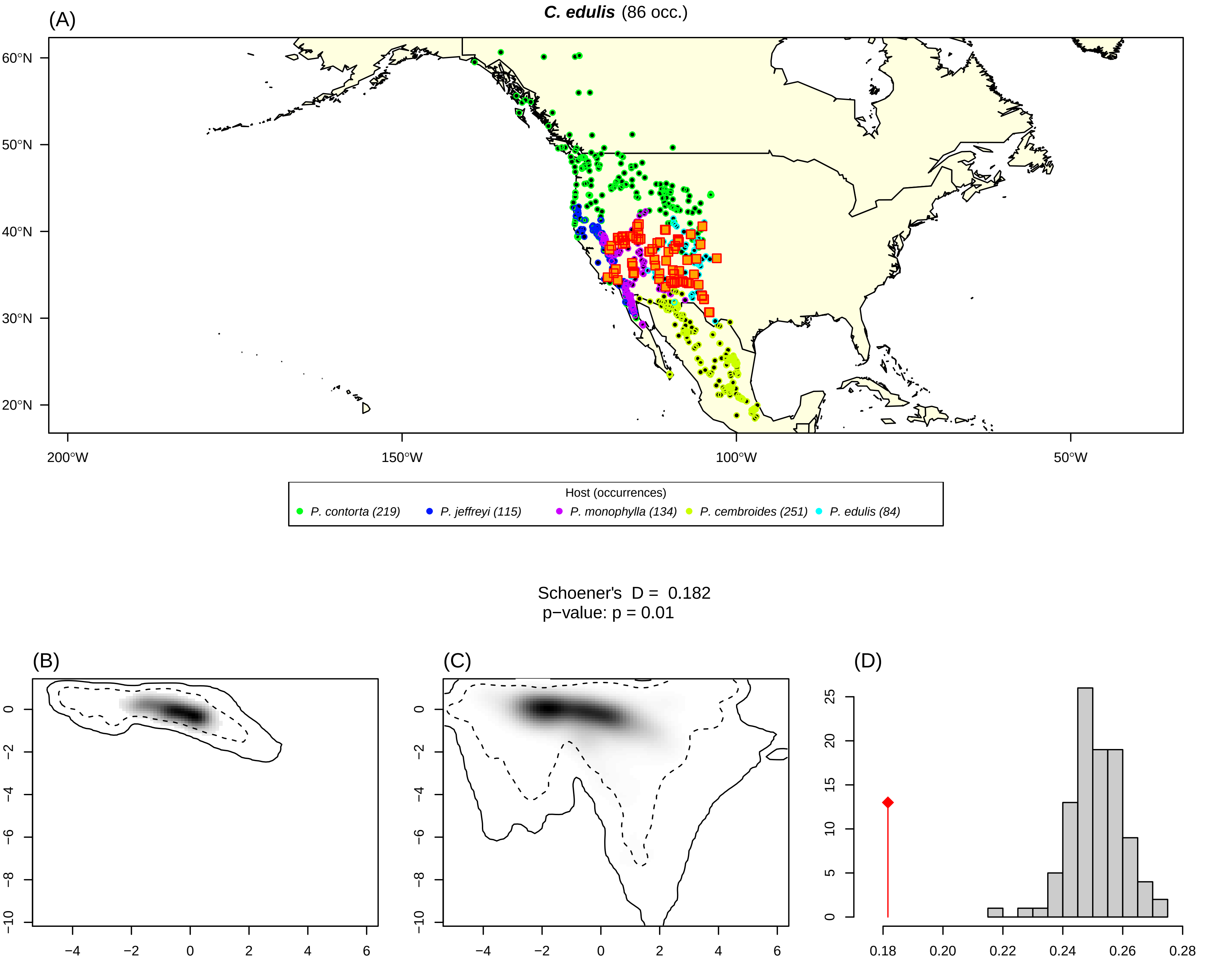

(A)
C. edulis (86 occ.)
60°N
50°N
40°N
30°N
20°N
200°W
150°W
100°W
50°W
Host (occurrences)
P. contorta (219)
P. jeffreyi (115)
P. monophylla (134)
P. cembroides (251)
P. edulis (84)
Schoener's D = 0.182
p−value: p = 0.01
(B)
(C)
(D)

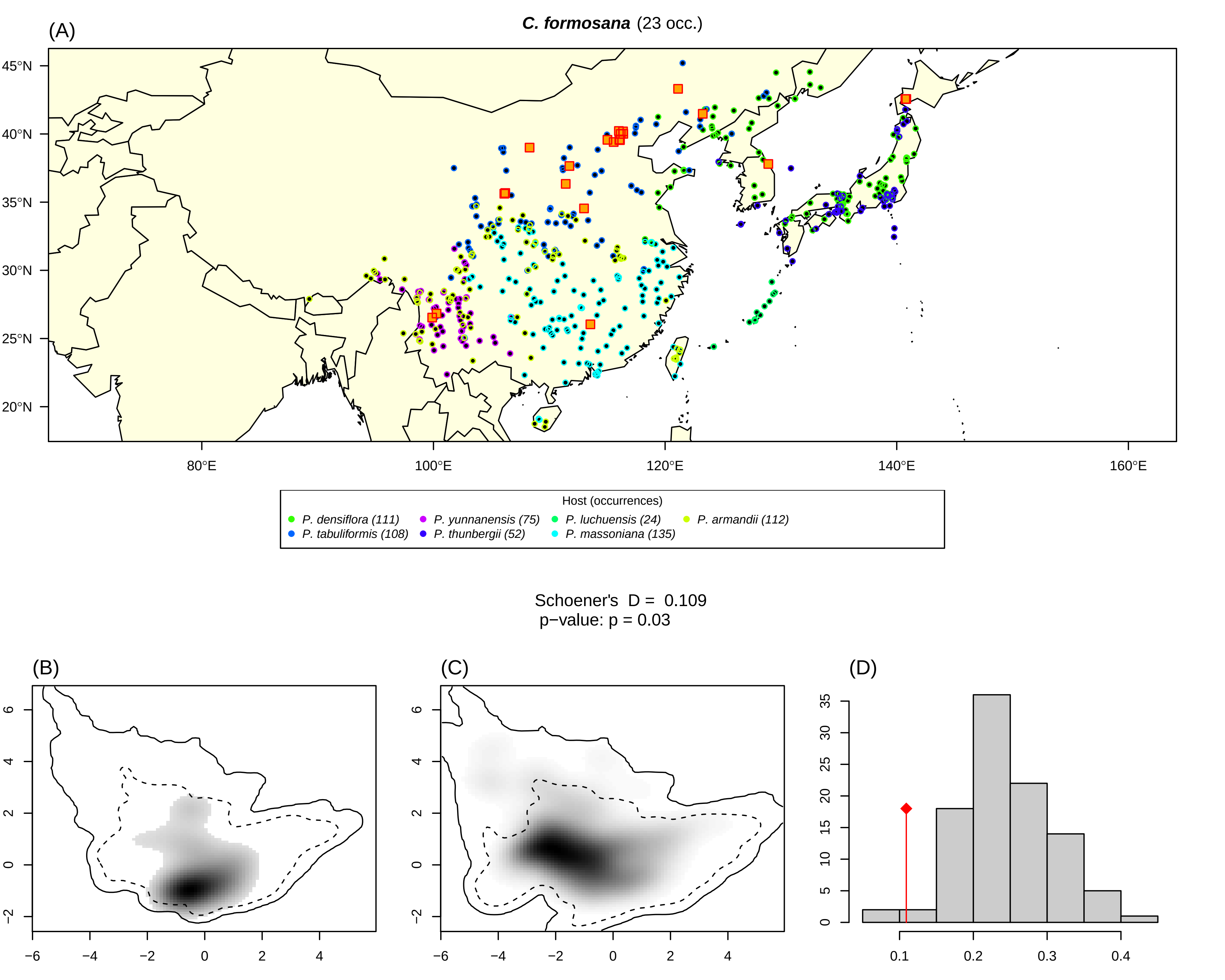

C. formosana (23 occ.)
(A)
45°N
40°N
35°N
30°N
25°N
20°N
80°E
100°E
120°E
140°E
160°E
Host (occurrences)
P. densiflora (111)
P. yunnanensis (75)
P. luchuensis (24)
P. armandii (112)
P. tabuliformis (108)
P. thunbergii (52)
P. massoniana (135)
Schoener's D = 0.109
p−value: p = 0.03
(B)
(C)
(D)

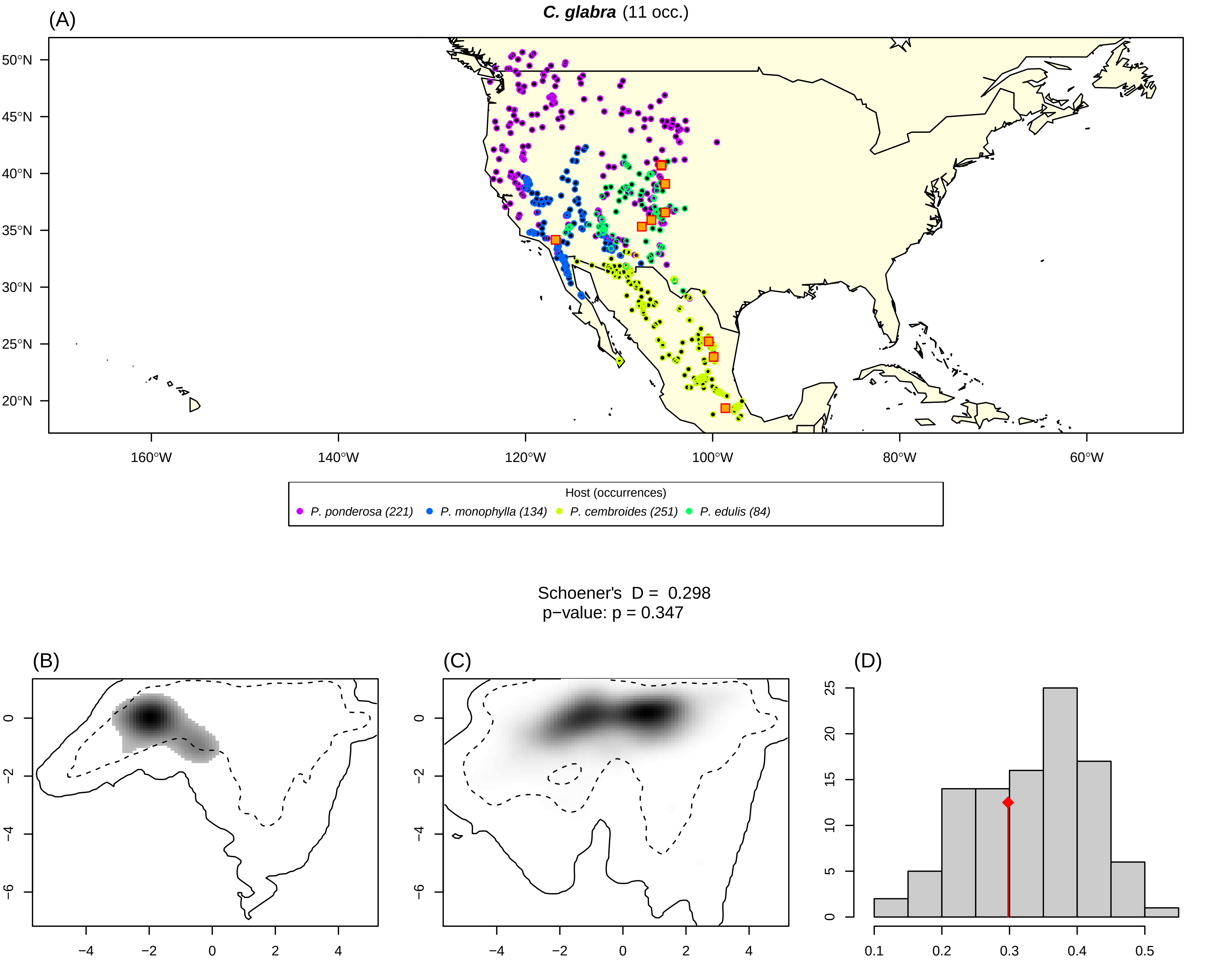

(A)
C. glabra (11 occ.)
50°N
45°N
40°N
35°N
30°N
25°N
20°N
160°W
140°W
120°W
100°W
80°W
60°W
Host (occurrences)
P. ponderosa (221)
P. monophylla (134)
P. cembroides (251)
P. edulis (84)
Schoener's D = 0.298
p−value: p = 0.347
(B)
(C)
(D)
0
−2
−4
−6
−4
−2
0
2
4
0
5
10
15
20
25
0.1
0.2
0.3
0.4
0.5

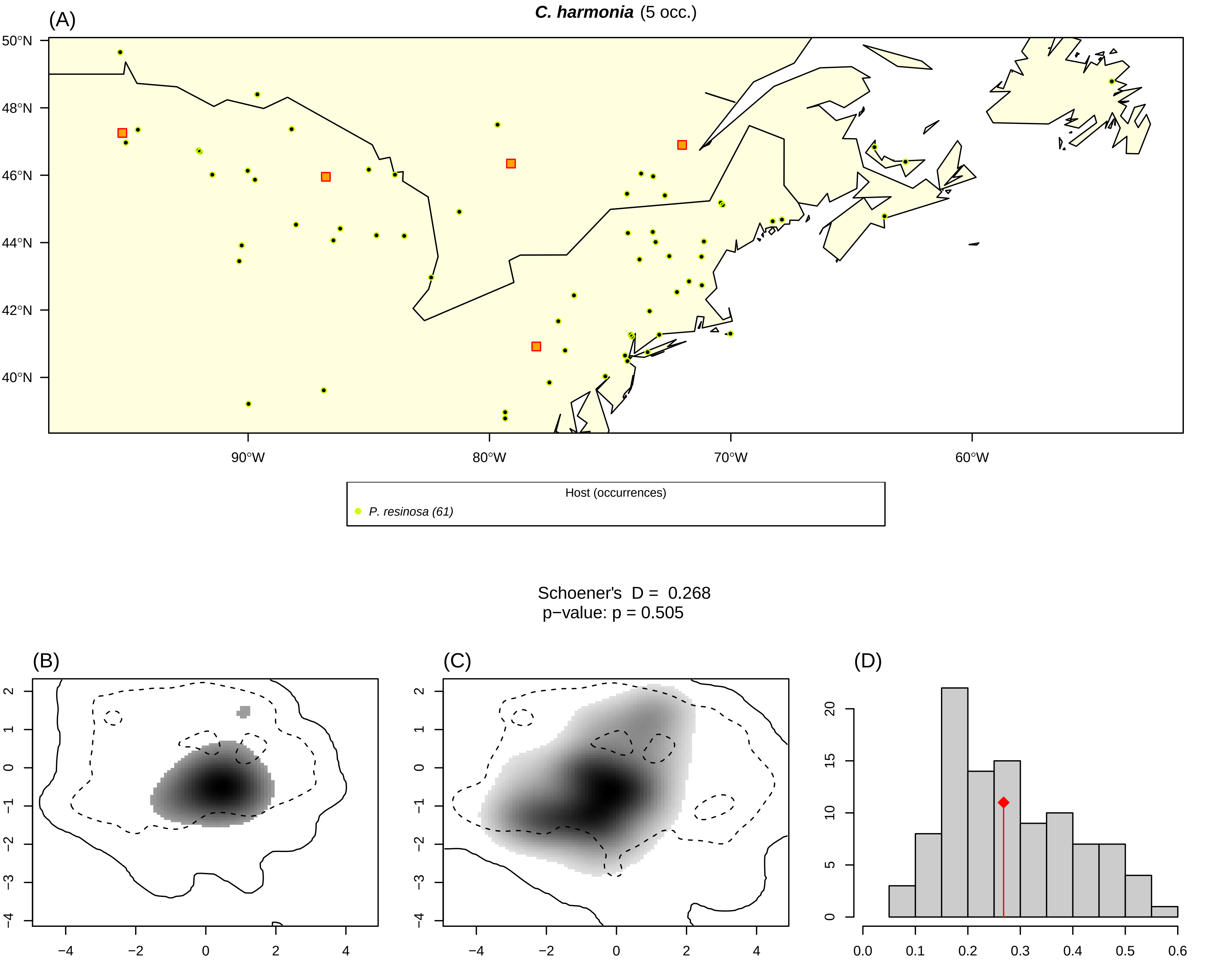
C. harmonia (5 occ.)
(A)
50°N
48°N
46°N
44°N
42°N
40°N
90°W
80°W
70°W
60°W
Host (occurrences)
P. resinosa (61)
Schoener's D = 0.268
p−value: p = 0.505
(B)
(C)
(D)
−4
−2
0
2
4
0.0
0.1
0.2
0.3
0.4
0.5
0.6
0
5
10
15
20

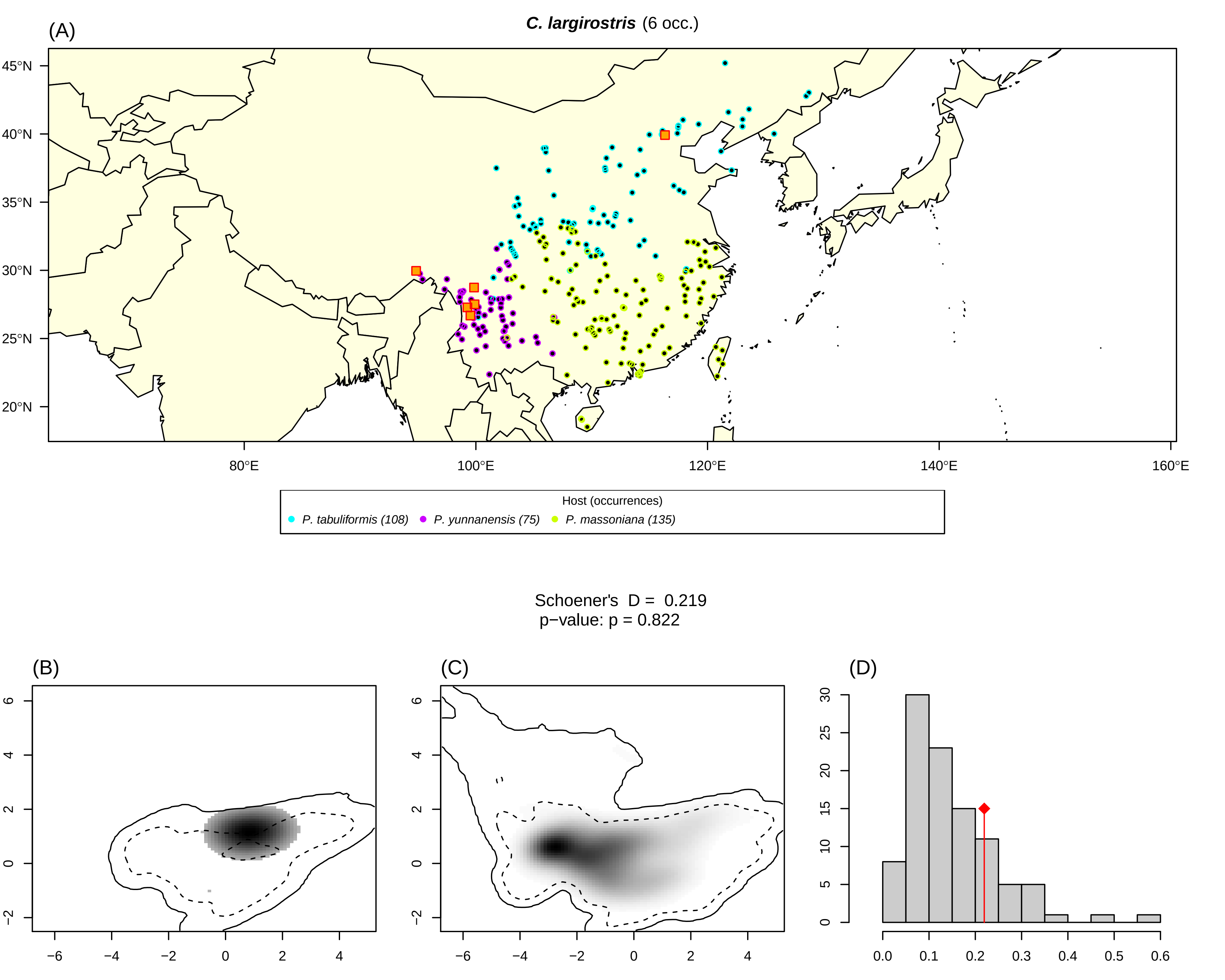

C. largirostris (6 occ.)
(A)
45°N
40°N
35°N
30°N
25°N
20°N
80°E
100°E
120°E
140°E
160°E
Host (occurrences)
P. tabuliformis (108)
P. yunnanensis (75)
P. massoniana (135)
Schoener's D = 0.219
p−value: p = 0.822
(B)
(C)
(D)

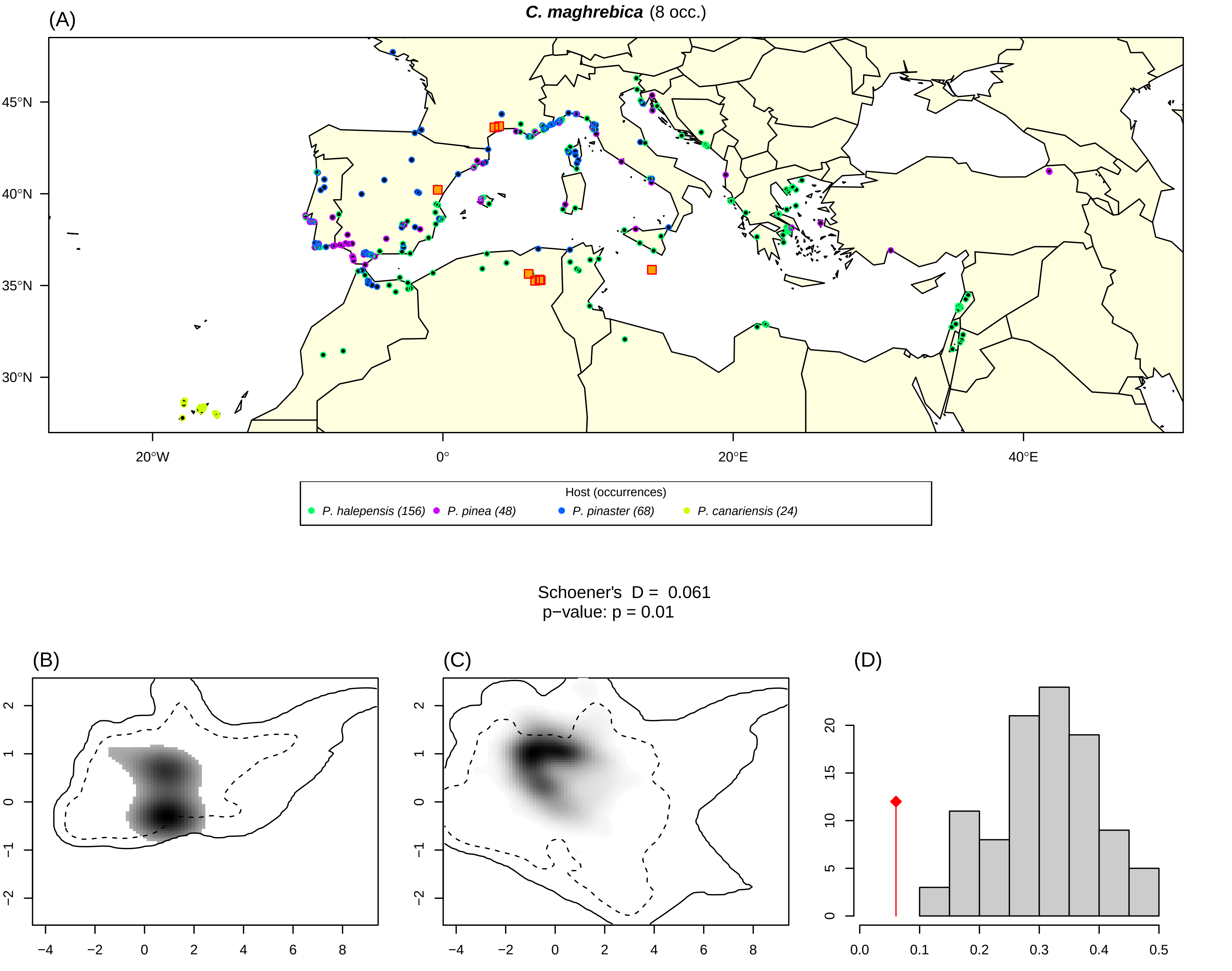
(A)
C. maghrebica (8 occ.)
45°N
40°N
35°N
30°N
20°W
0°
20°E
40°E
Host (occurrences)
P. halepensis (156)
P. pinea (48)
P. pinaster (68)
P. canariensis (24)
Schoener's D = 0.061
p−value: p = 0.01
(B)
(C)
(D)

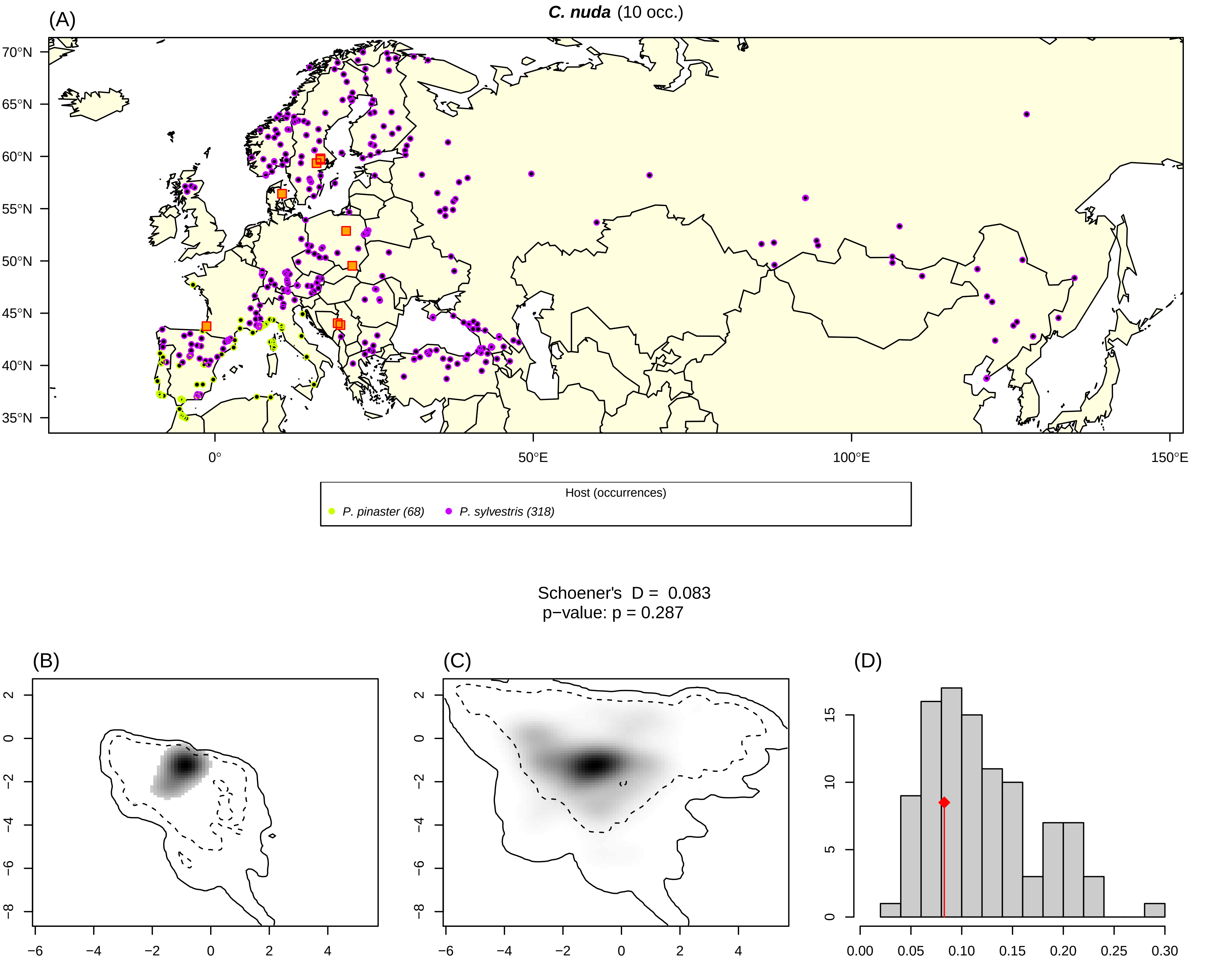

C. nuda (10 occ.)
(A)
70°N
65°N
60°N
55°N
50°N
45°N
40°N
35°N
0°
50°E
100°E
150°E
Host (occurrences)
P. pinaster (68)
P. sylvestris (318)
Schoener's D = 0.083
p−value: p = 0.287
(B)
(C)
(D)

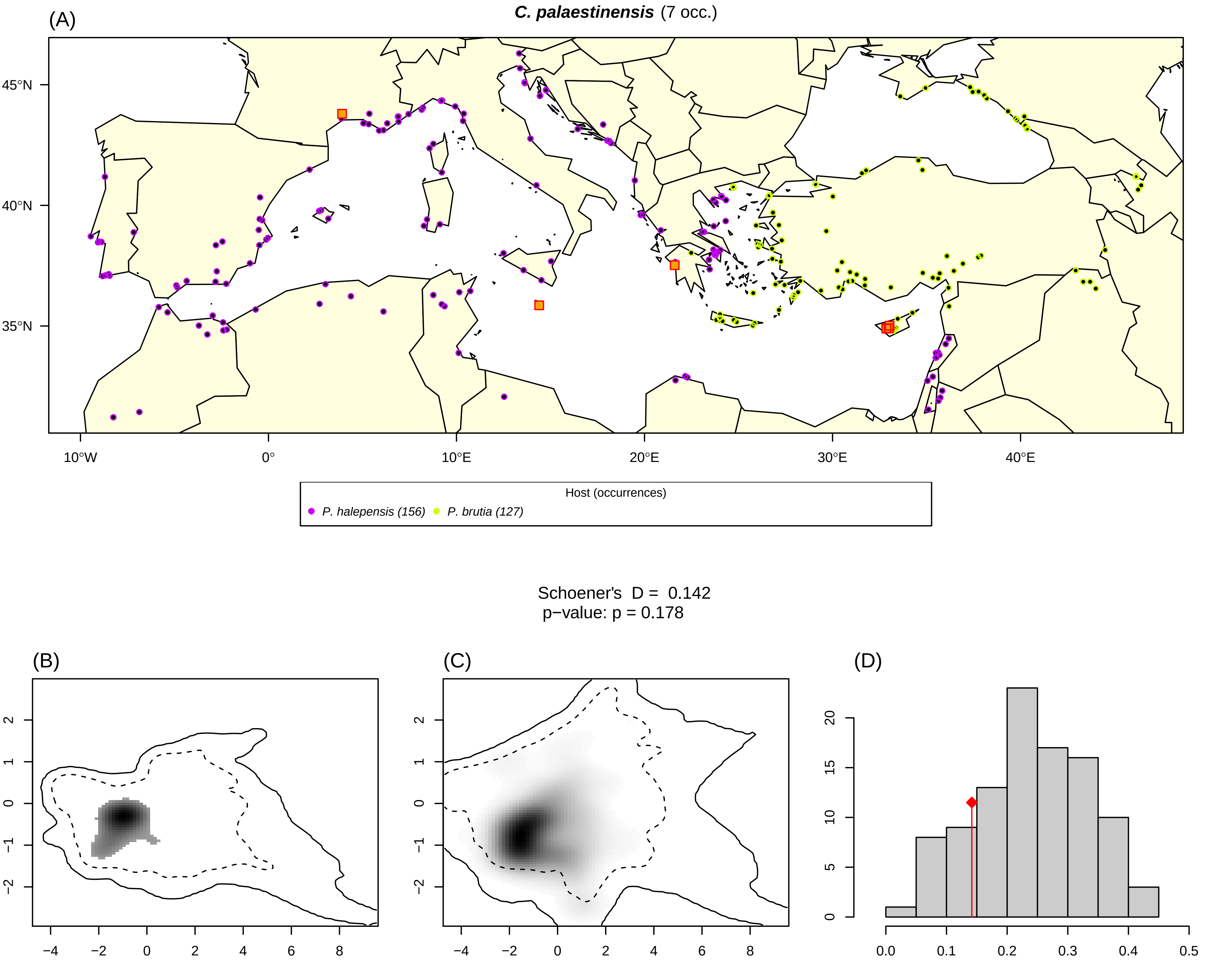

C. palaestinensis (7 occ.)
(A)
45°N
40°N
35°N
10°W
0°
10°E
20°E
30°E
40°E
Host (occurrences)
P. halepensis (156)
P. brutia (127)
Schoener's D = 0.142
p−value: p = 0.178
(B)
(C)
(D)

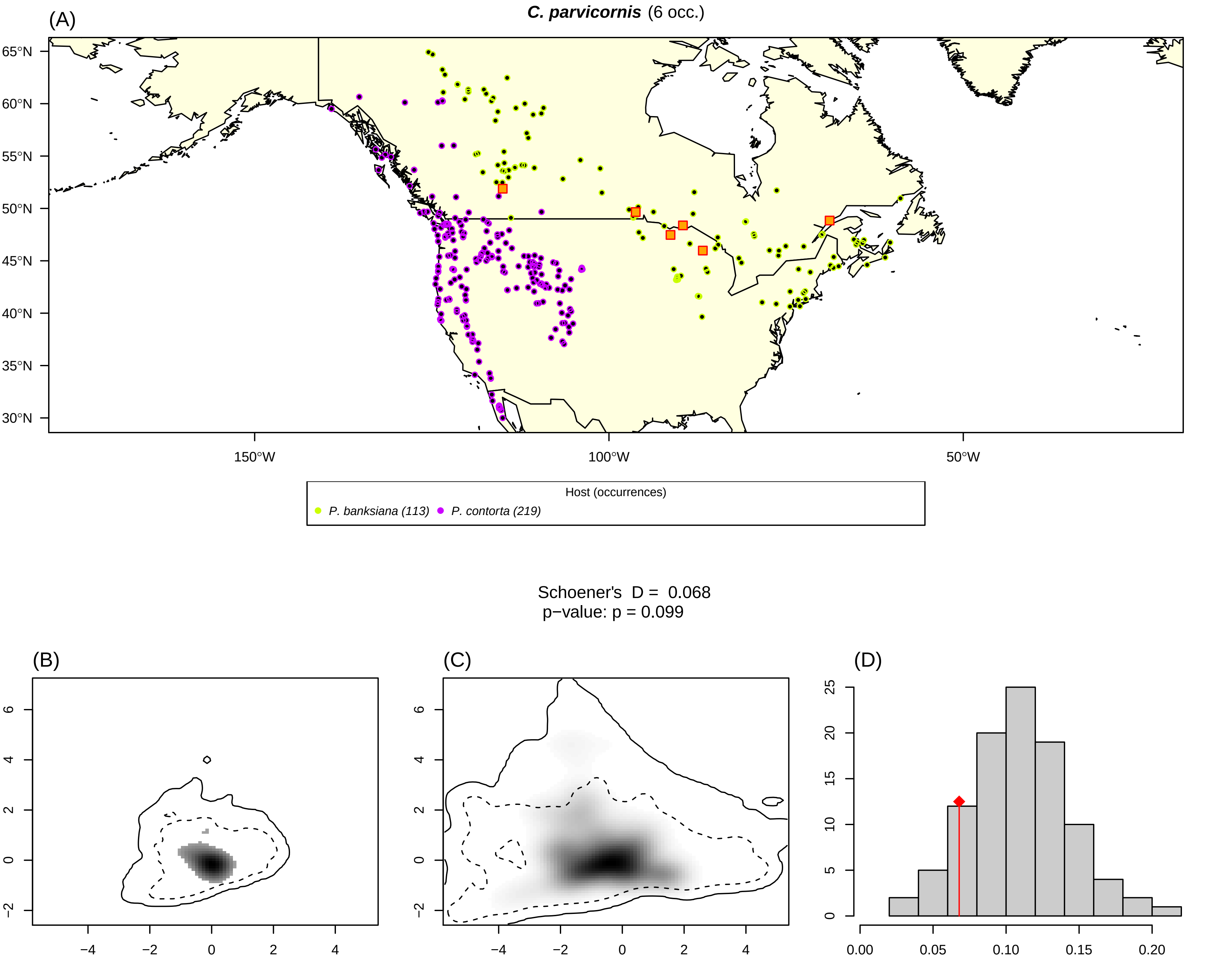

C. parvicornis (6 occ.)
(A)
65°N
60°N
55°N
50°N
45°N
40°N
35°N
30°N
150°W
100°W
50°W
Host (occurrences)
P. banksiana (113)
P. contorta (219)
Schoener's D = 0.068
p−value: p = 0.099
(B)
(C)
(D)

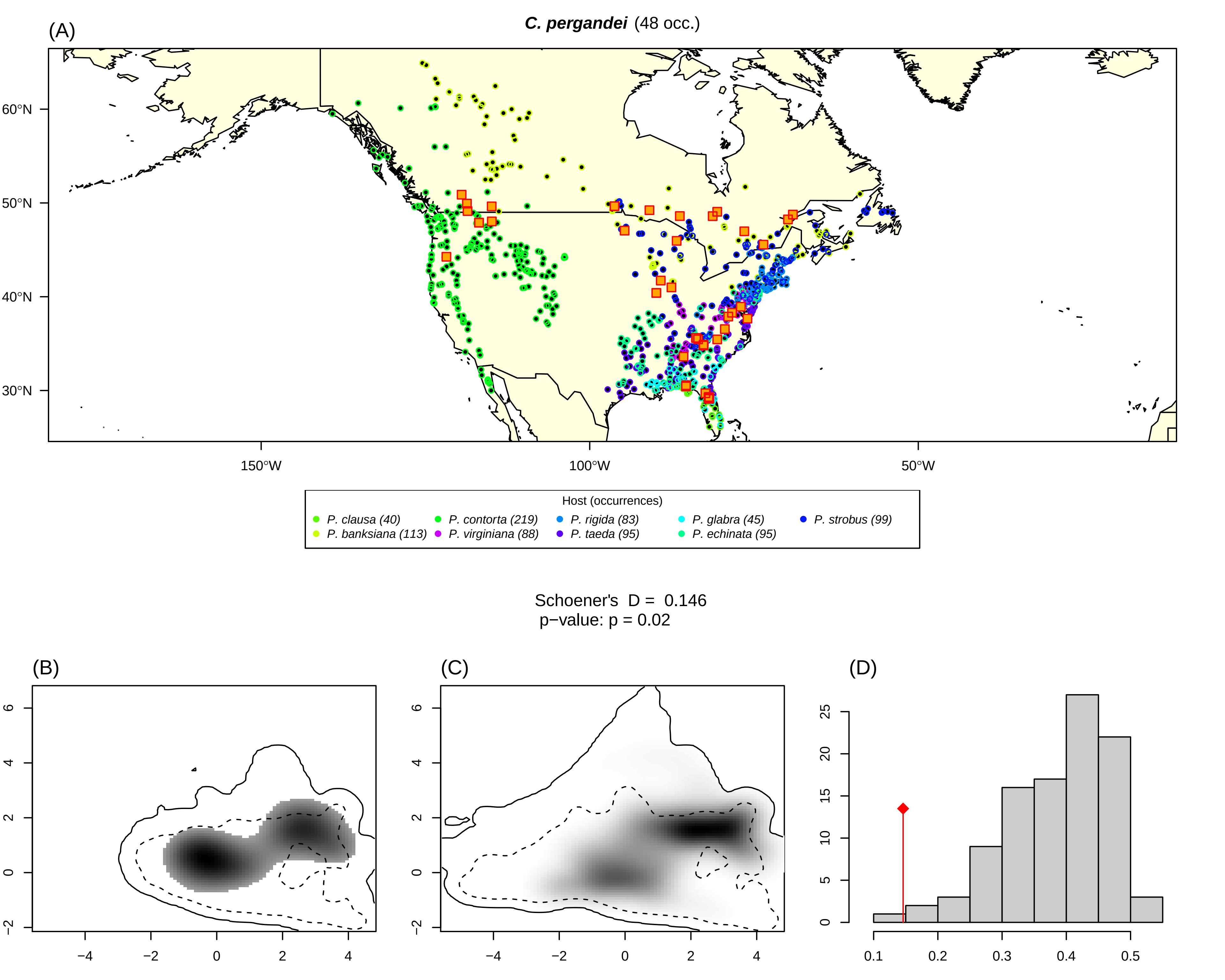

(A)
C. pergandei (48 occ.)
60°N
50°N
40°N
30°N
150°W
100°W
50°W
Host (occurrences)
P. clausa (40)
P. banksiana (113)
P. contorta (219)
P. virginiana (88)
P. rigida (83)
P. taeda (95)
P. glabra (45)
P. echinata (95)
P. strobus (99)
Schoener's D = 0.146
p−value: p = 0.02
(B)
(C)
(D)
−4 −2 0 2 4
−2 0 2 4 6
0 5 10 15 20 25
0.1 0.2 0.3 0.4 0.5

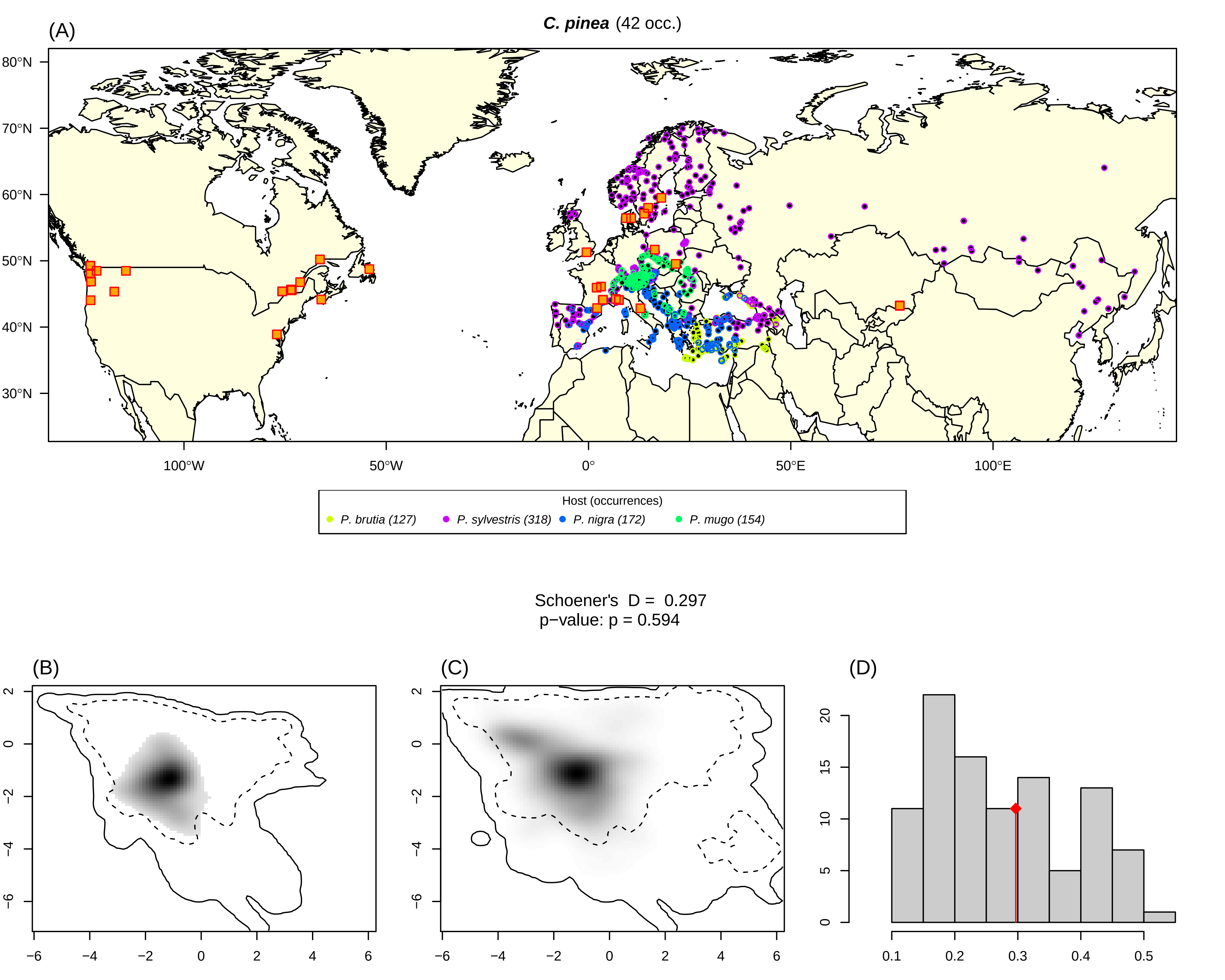

(A)
C. pinea (42 occ.)
80°N
70°N
60°N
50°N
40°N
30°N
100°W
50°W
0°
50°E
100°E
Host (occurrences)
P. brutia (127)
P. sylvestris (318)
P. nigra (172)
P. mugo (154)
Schoener's D = 0.297
p−value: p = 0.594
(B)
(C)
(D)

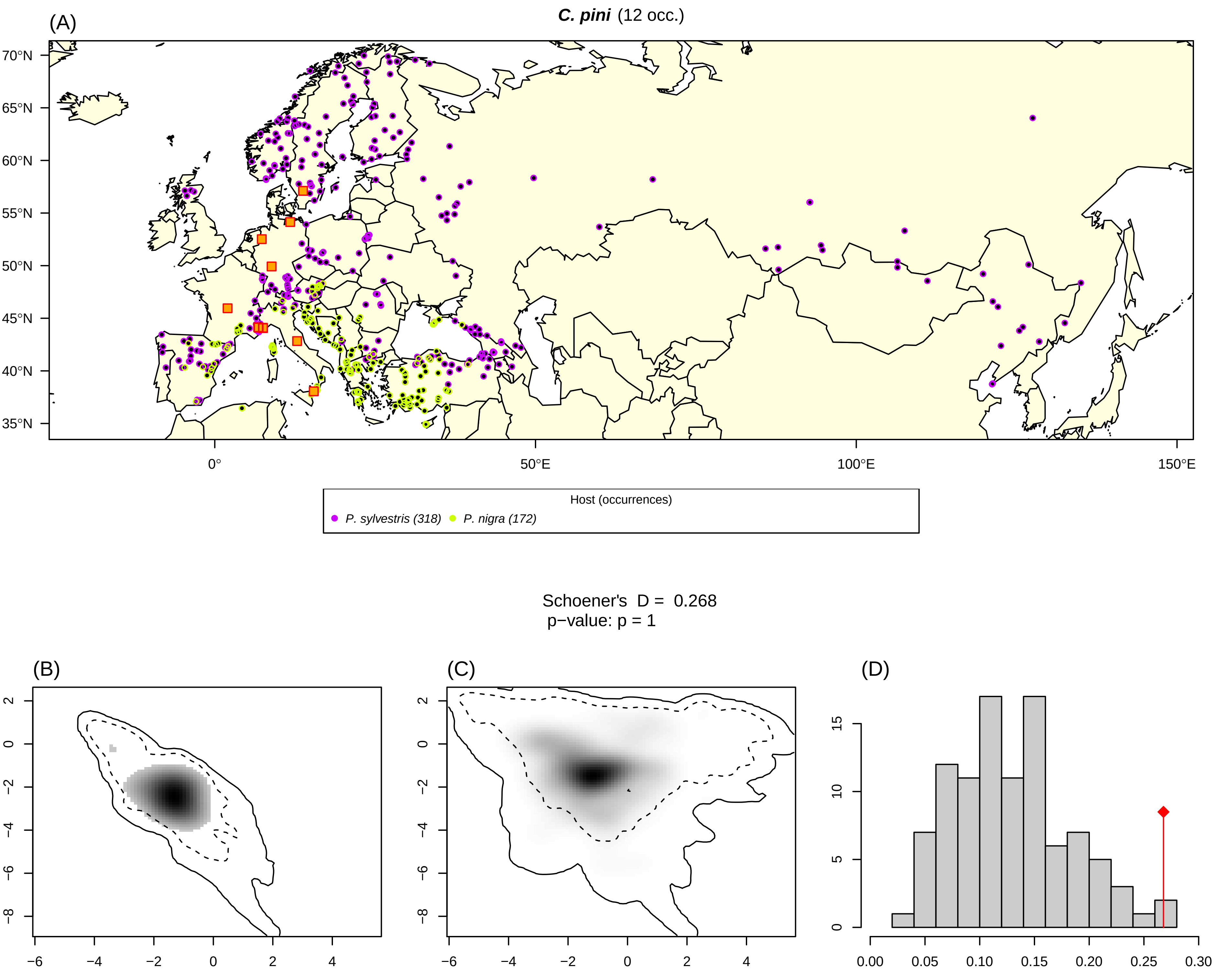
(A)
C. pini (12 occ.)
70°N
65°N
60°N
55°N
50°N
45°N
40°N
35°N
0°
50°E
100°E
150°E
Host (occurrences)
P. sylvestris (318)
P. nigra (172)
Schoener's D = 0.268
p−value: p = 1
(B)
(C)
(D)
2
0
−2
−4
−6
−8
−6
−4
−2
0
2
4
15
10
5
0
0.00
0.05
0.10
0.15
0.20
0.25
0.30

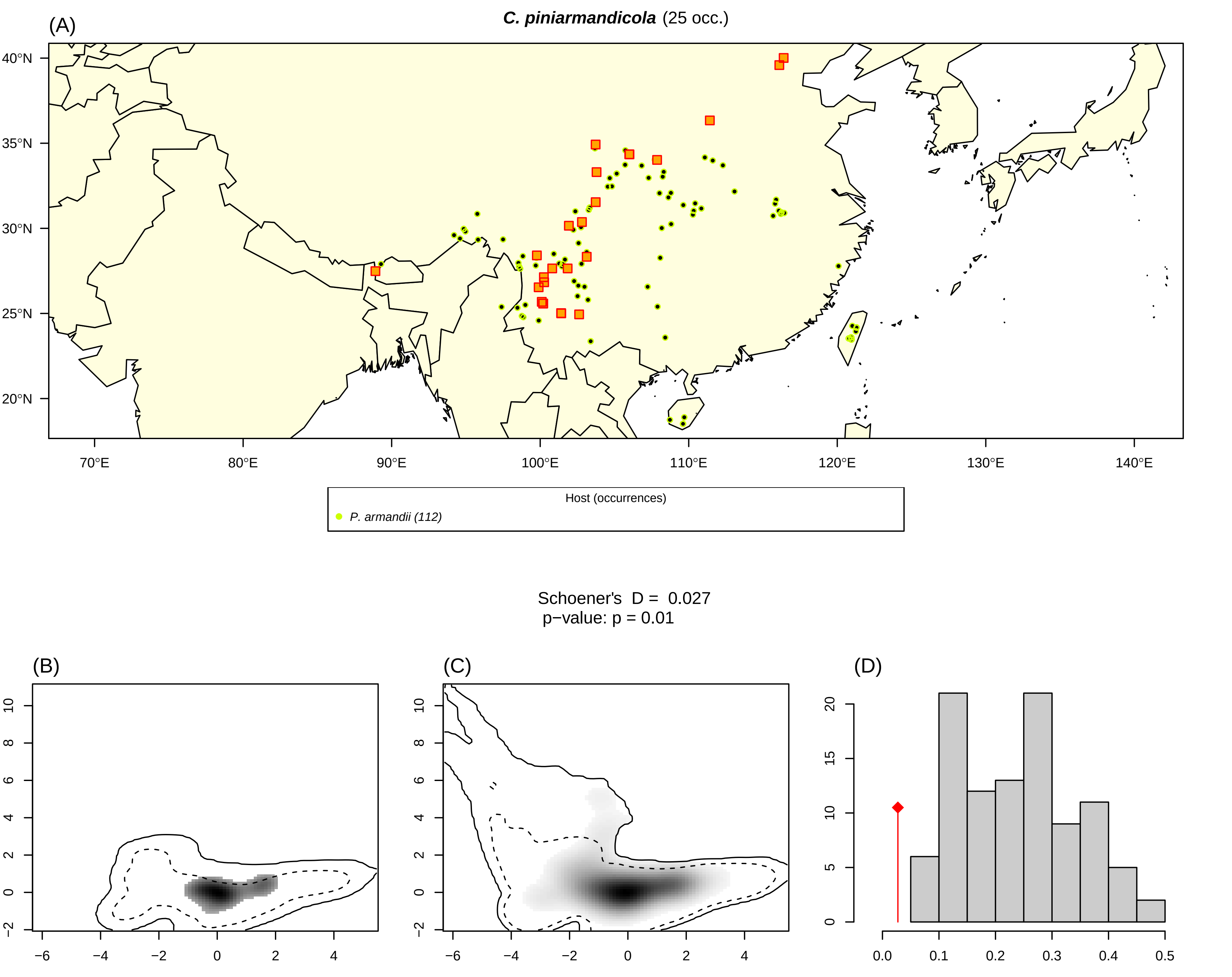

C. piniarmandicola (25 occ.)
(A)
40°N
35°N
30°N
25°N
20°N
70°E
80°E
90°E
100°E
110°E
120°E
130°E
140°E
Host (occurrences)
P. armandii (112)
Schoener's D = 0.027
p−value: p = 0.01
(B)
(C)
(D)

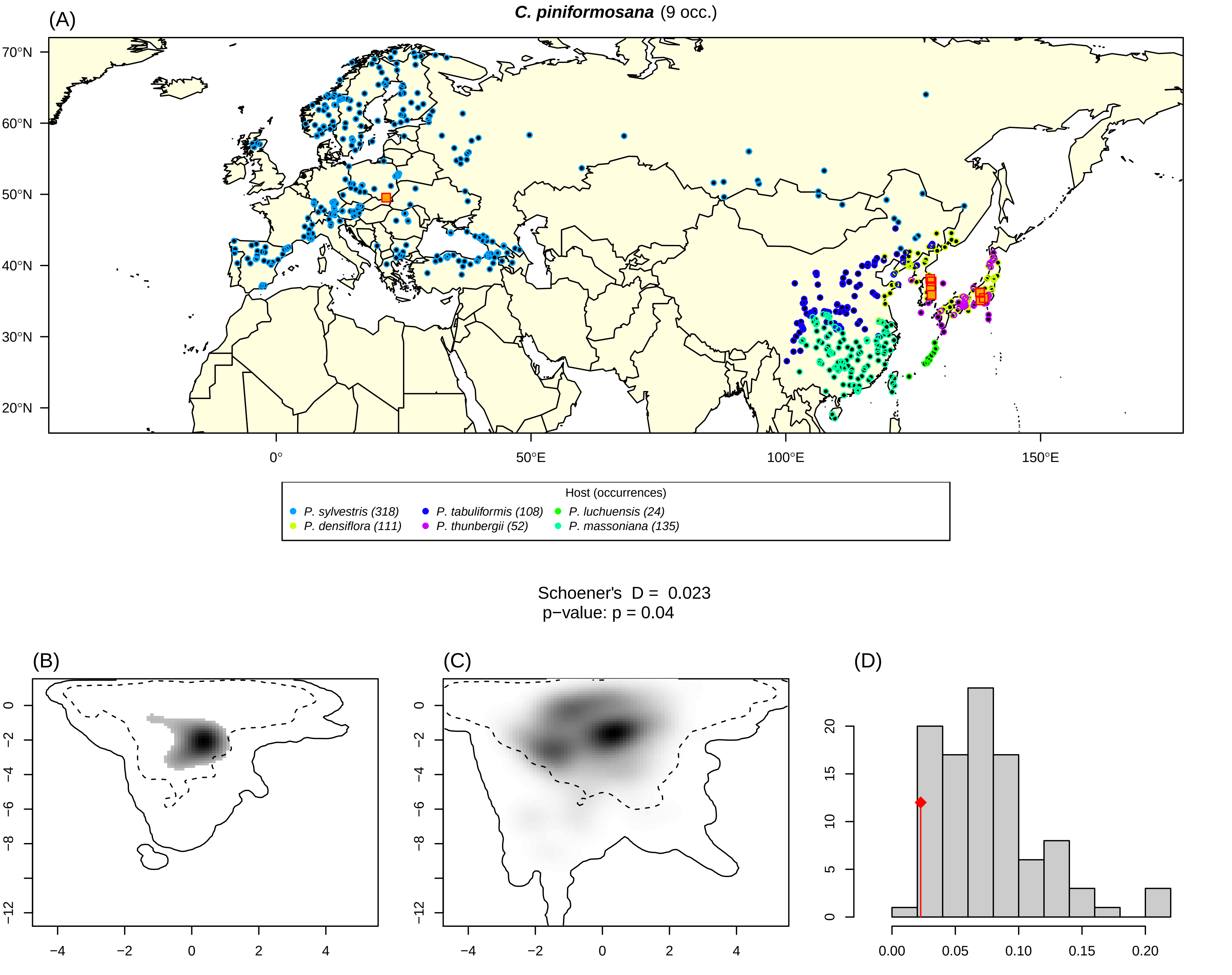

C. piniformosana (9 occ.)
(A)
70°N
60°N
50°N
40°N
30°N
20°N
0°
50°E
100°E
150°E
Host (occurrences)
P. sylvestris (318)
P. tabuliformis (108)
P. luchuensis (24)
P. densiflora (111)
P. thunbergii (52)
P. massoniana (135)
Schoener's D = 0.023
p−value: p = 0.04
(B)
(C)
(D)

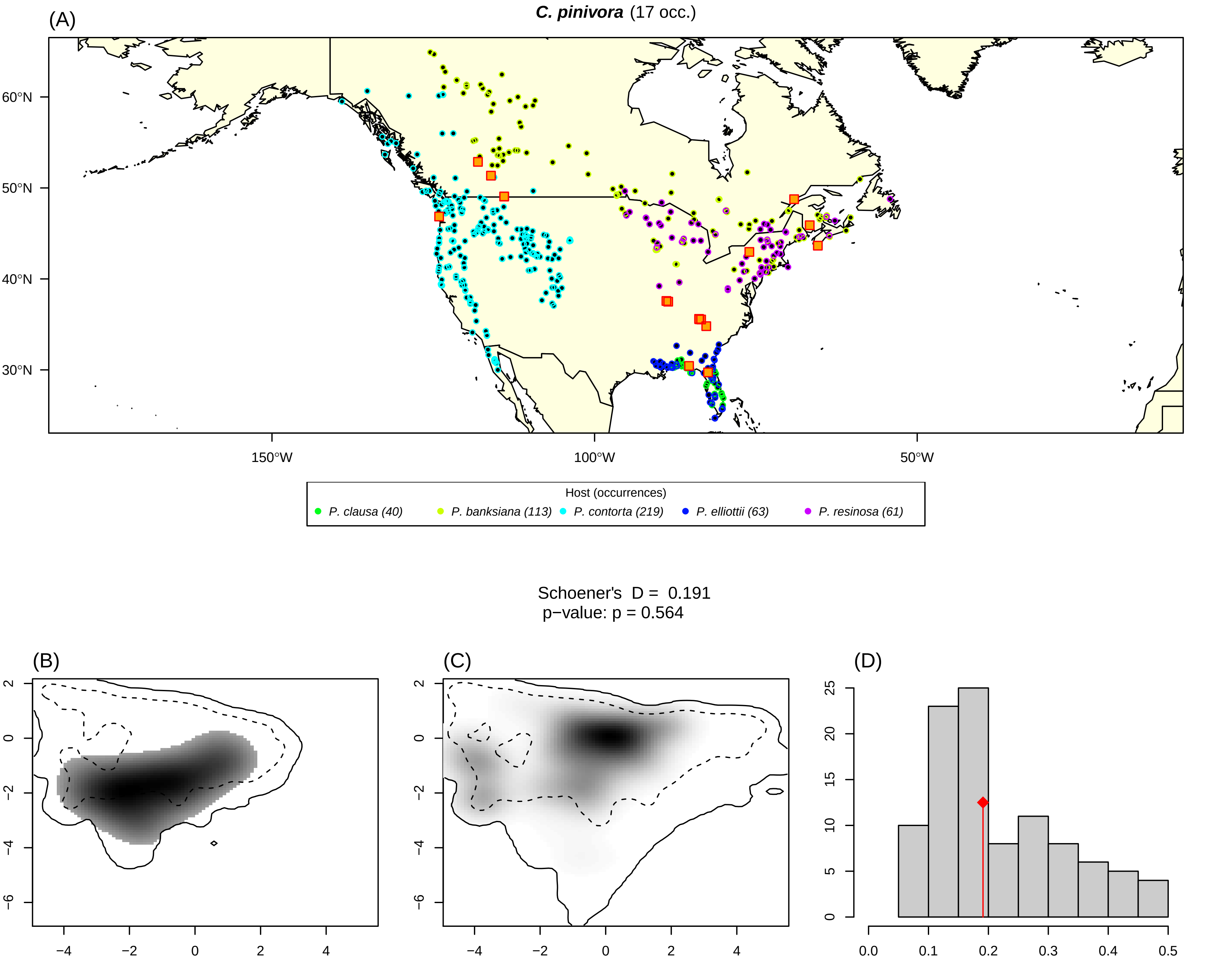

(A)
C. pinivora (17 occ.)
60°N
50°N
40°N
30°N
150°W
100°W
50°W
Host (occurrences)
P. clausa (40)
P. banksiana (113)
P. contorta (219)
P. elliottii (63)
P. resinosa (61)
Schoener's D = 0.191
p−value: p = 0.564
(B)
(C)
(D)

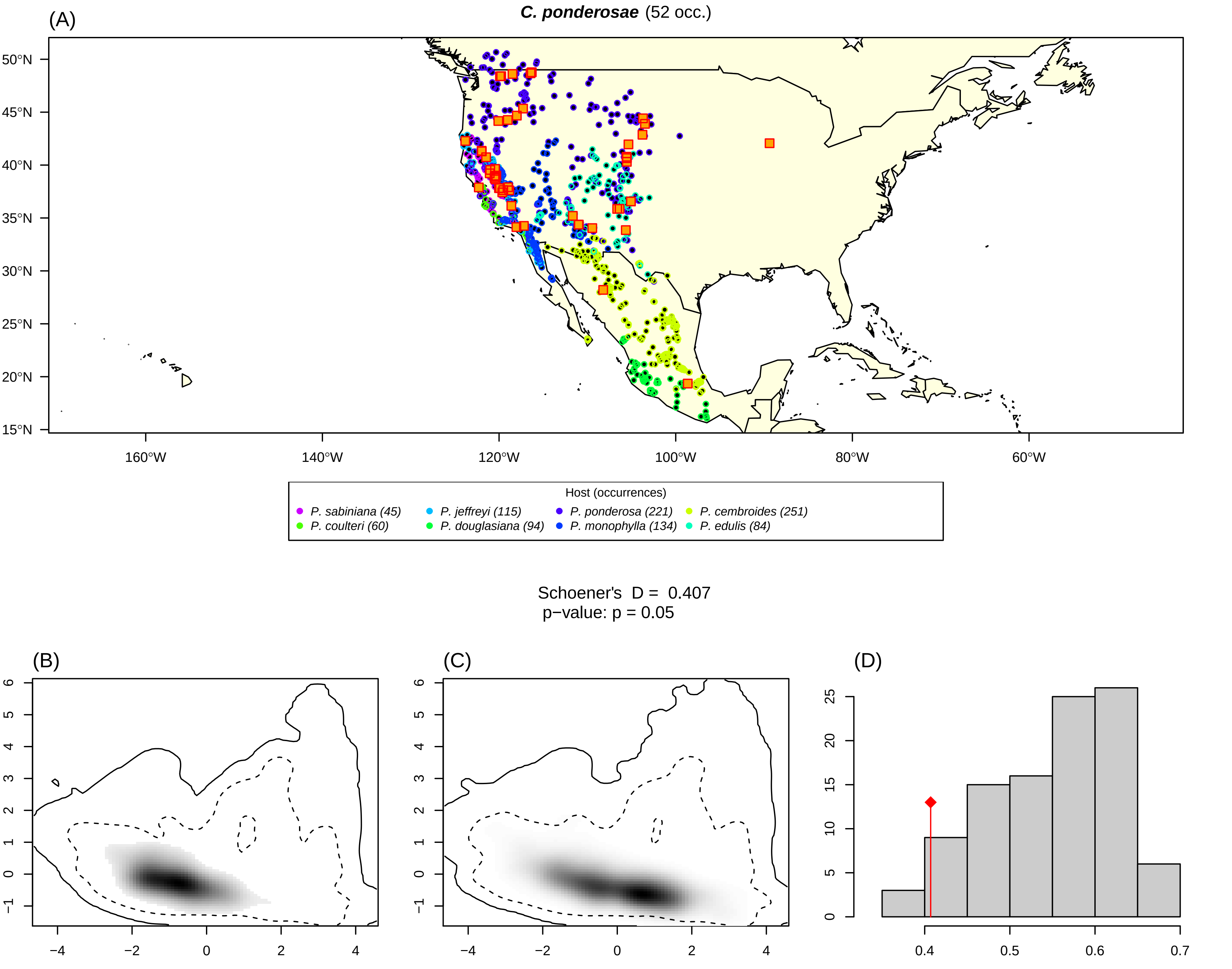
(A)
*C. ponderosae* (52 occ.)
50°N
45°N
40°N
35°N
30°N
25°N
20°N
15°N
160°W
140°W
120°W
100°W
80°W
60°W
Host (occurrences)
*P. sabiniana (45)*
*P. coulteri (60)*
*P. jeffreyi (115)*
*P. douglasiana (94)*
*P. ponderosa (221)*
*P. monophylla (134)*
*P. cembroides (251)*
*P. edulis (84)*
Schoener's D = 0.407
p−value: p = 0.05
(B)
(C)
(D)

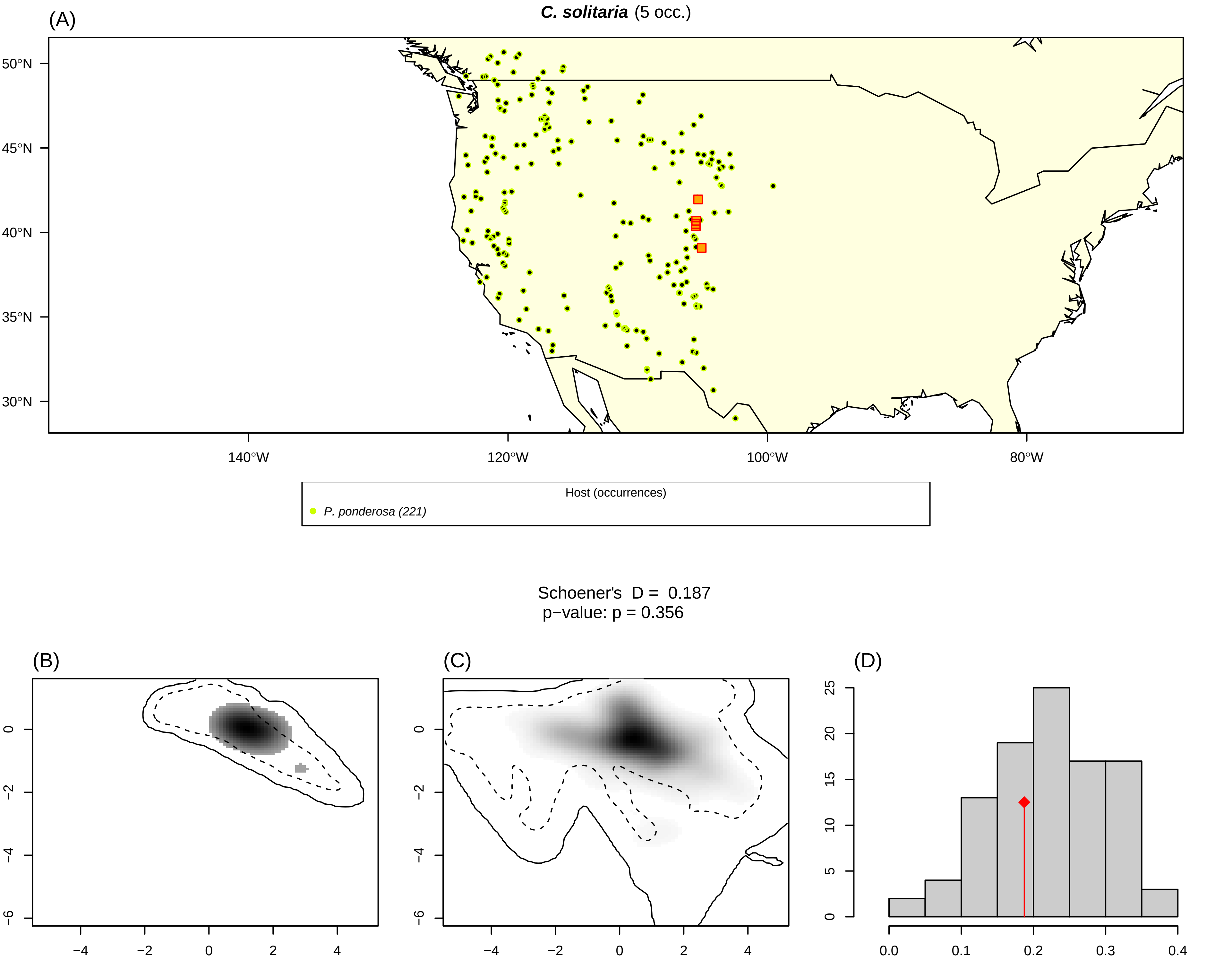

(A)
C. solitaria (5 occ.)
50°N
45°N
40°N
35°N
30°N
140°W
120°W
100°W
80°W
Host (occurrences)
P. ponderosa (221)
Schoener's D = 0.187
p−value: p = 0.356
(B)
(C)
(D)
−6
−4
−2
0
2
4
0
5
10
15
20
25
0.0
0.1
0.2
0.3
0.4

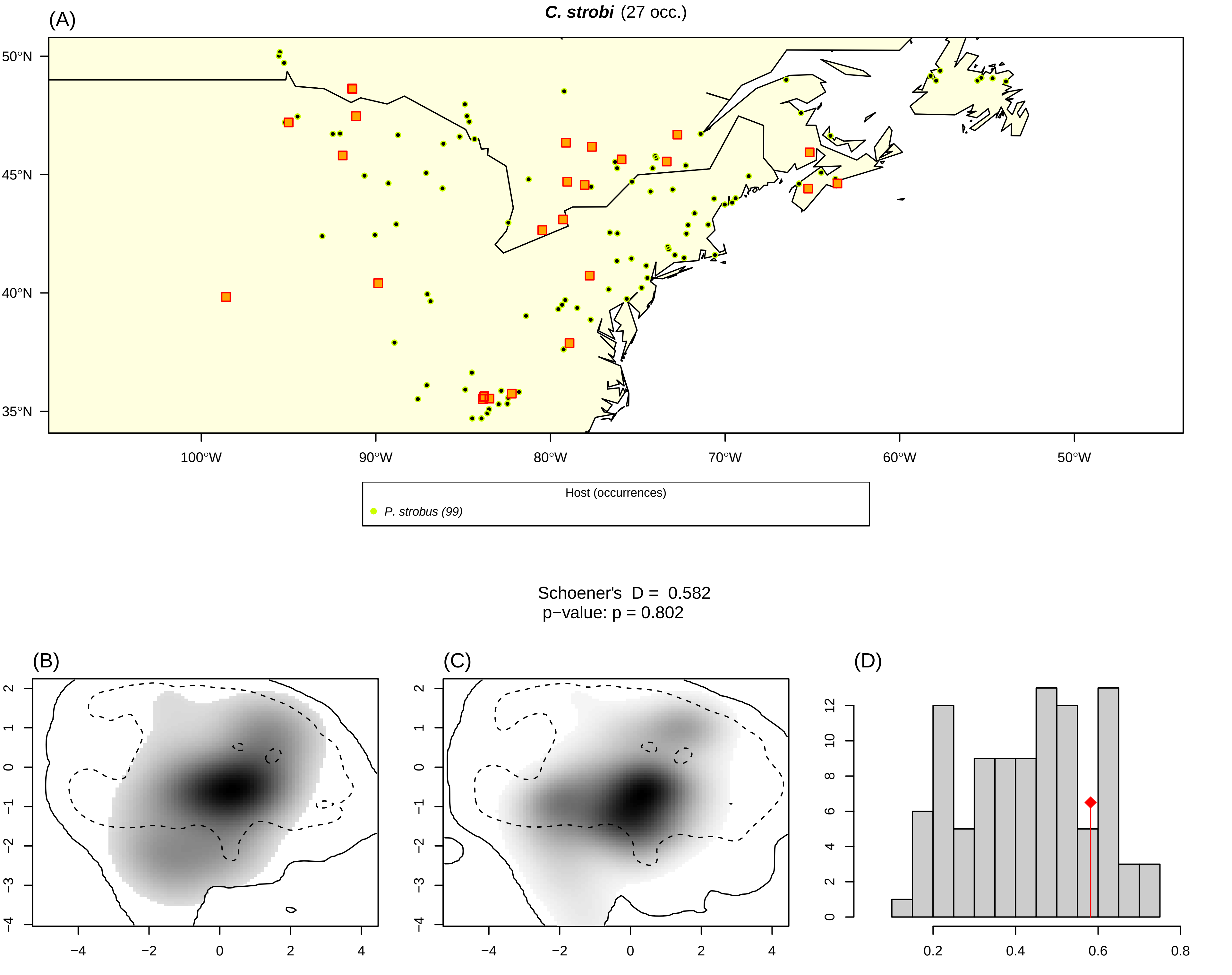
C. strobi (27 occ.)
(A)
50°N
45°N
40°N
35°N
100°W
90°W
80°W
70°W
60°W
50°W
Host (occurrences)
P. strobus (99)
Schoener's D = 0.582
p−value: p = 0.802
(B)
(C)
(D)

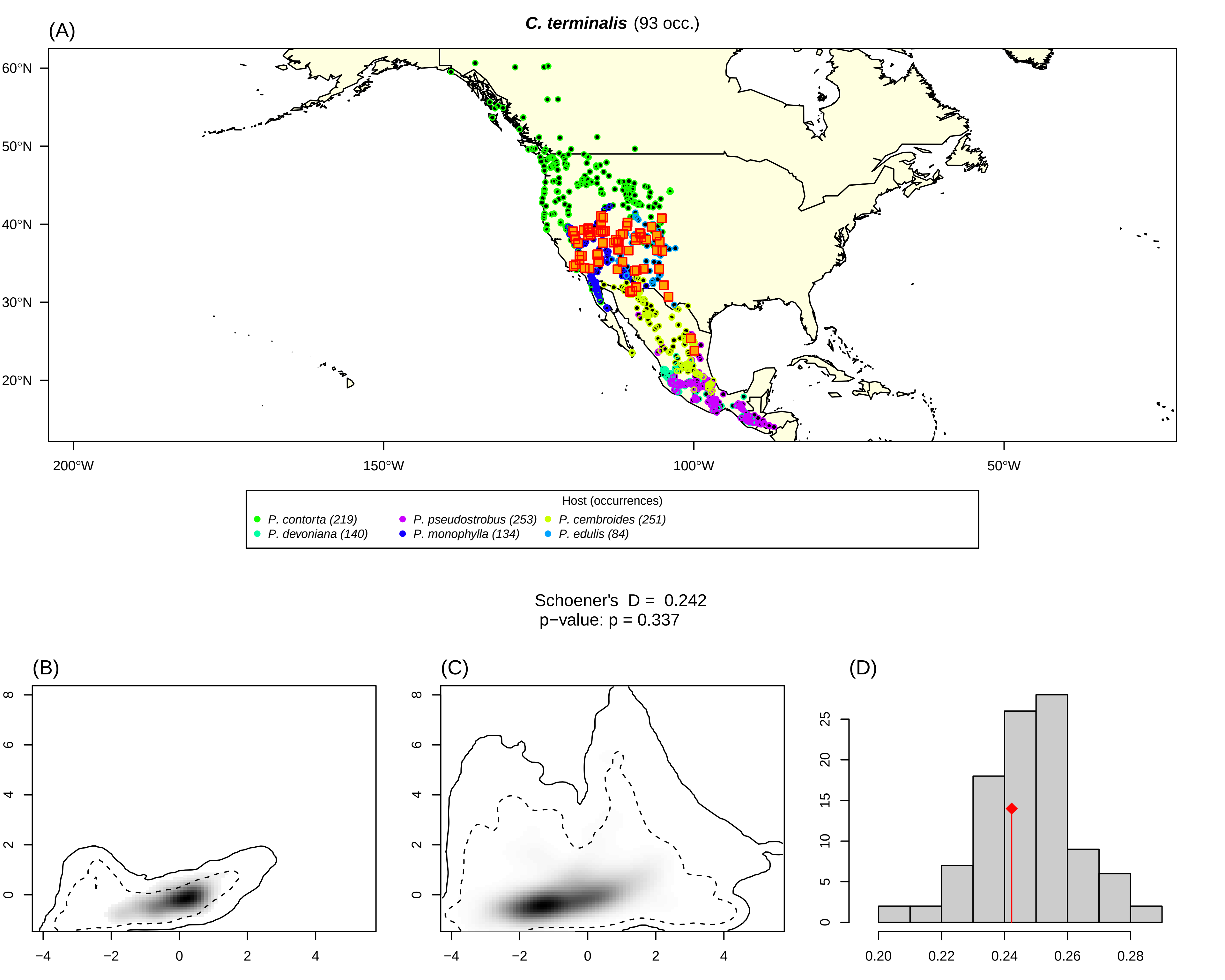

(A)
C. terminalis (93 occ.)
60°N
50°N
40°N
30°N
20°N
200°W
150°W
100°W
50°W
Host (occurrences)
P. contorta (219)
P. devoniana (140)
P. pseudostrobus (253)
P. monophylla (134)
P. cembroides (251)
P. edulis (84)
Schoener's D = 0.242
p−value: p = 0.337
(B)
(C)
(D)

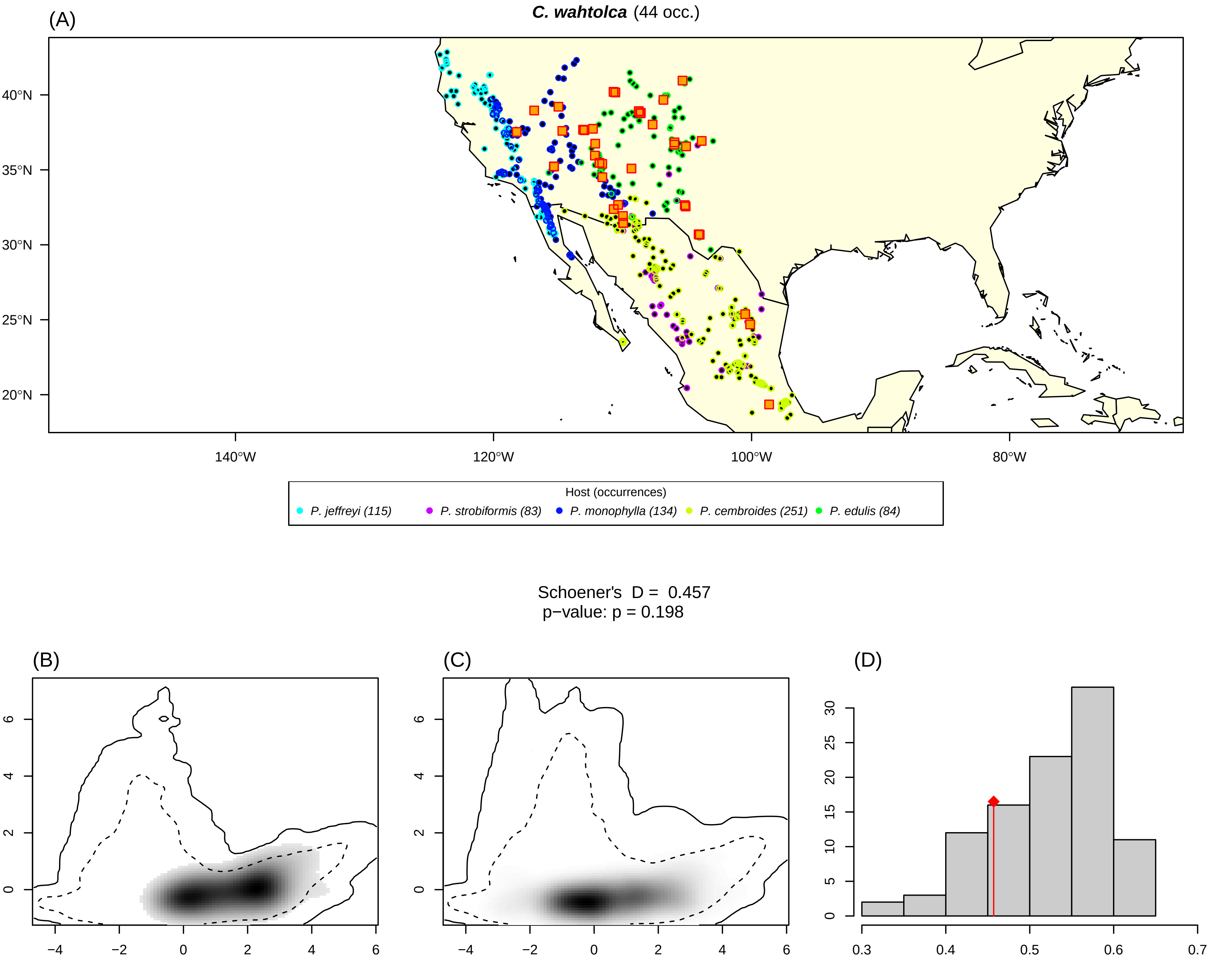

C. wahtolca (44 occ.)
(A)
40°N
35°N
30°N
25°N
20°N
140°W
120°W
100°W
80°W
Host (occurrences)
P. jeffreyi (115)
P. strobiformis (83)
P. monophylla (134)
P. cembroides (251)
P. edulis (84)
Schoener's D = 0.457
p−value: p = 0.198
(B)
(C)
(D)

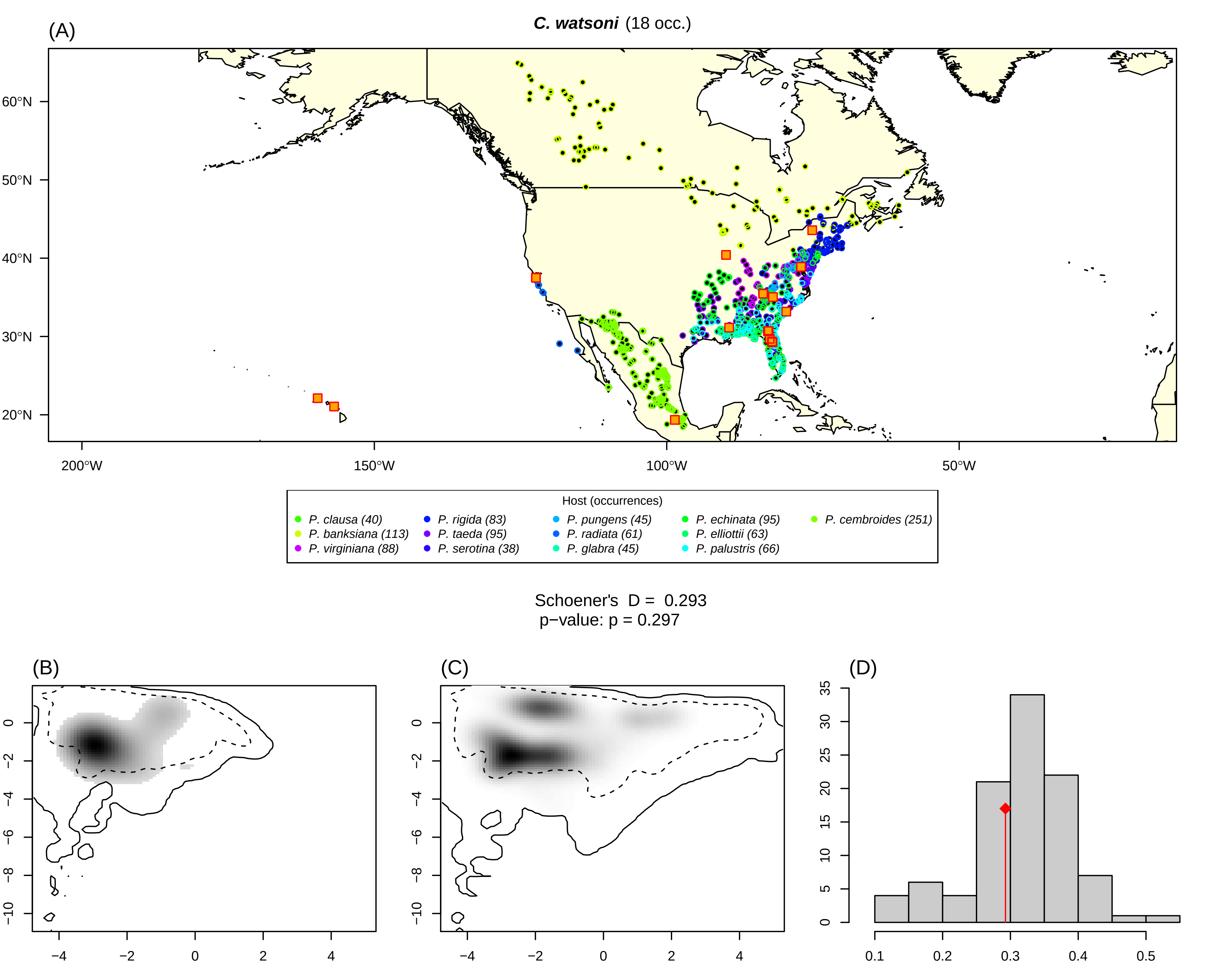

*C. watsoni* (18 occ.)
(A)
60°N
50°N
40°N
30°N
20°N
200°W
150°W
100°W
50°W
Host (occurrences)
*P. clausa (40)*
*P. banksiana (113)*
*P. virginiana (88)*
*P. rigida (83)*
*P. taeda (95)*
*P. serotina (38)*
*P. pungens (45)*
*P. radiata (61)*
*P. glabra (45)*
*P. echinata (95)*
*P. elliottii (63)*
*P. palustris (66)*
*P. cembroides (251)*
Schoener's D = 0.293
p−value: p = 0.297
(B)
(C)
(D)

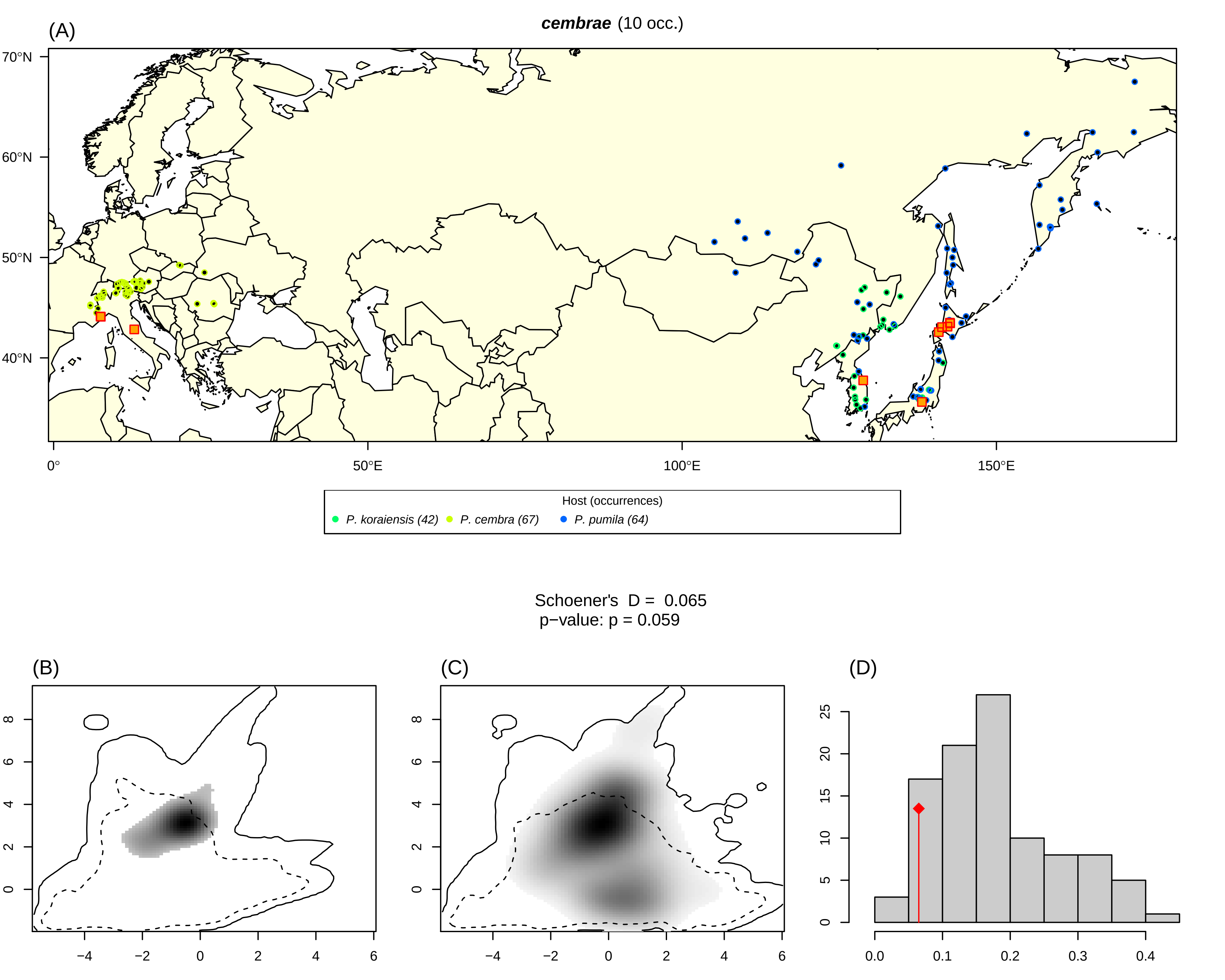
(A)
cembrae (10 occ.)
70°N
60°N
50°N
40°N
0°
50°E
100°E
150°E
Host (occurrences)
P. koraiensis (42)
P. cembra (67)
P. pumila (64)
Schoener's D = 0.065
p−value: p = 0.059
(B)
(C)
(D)

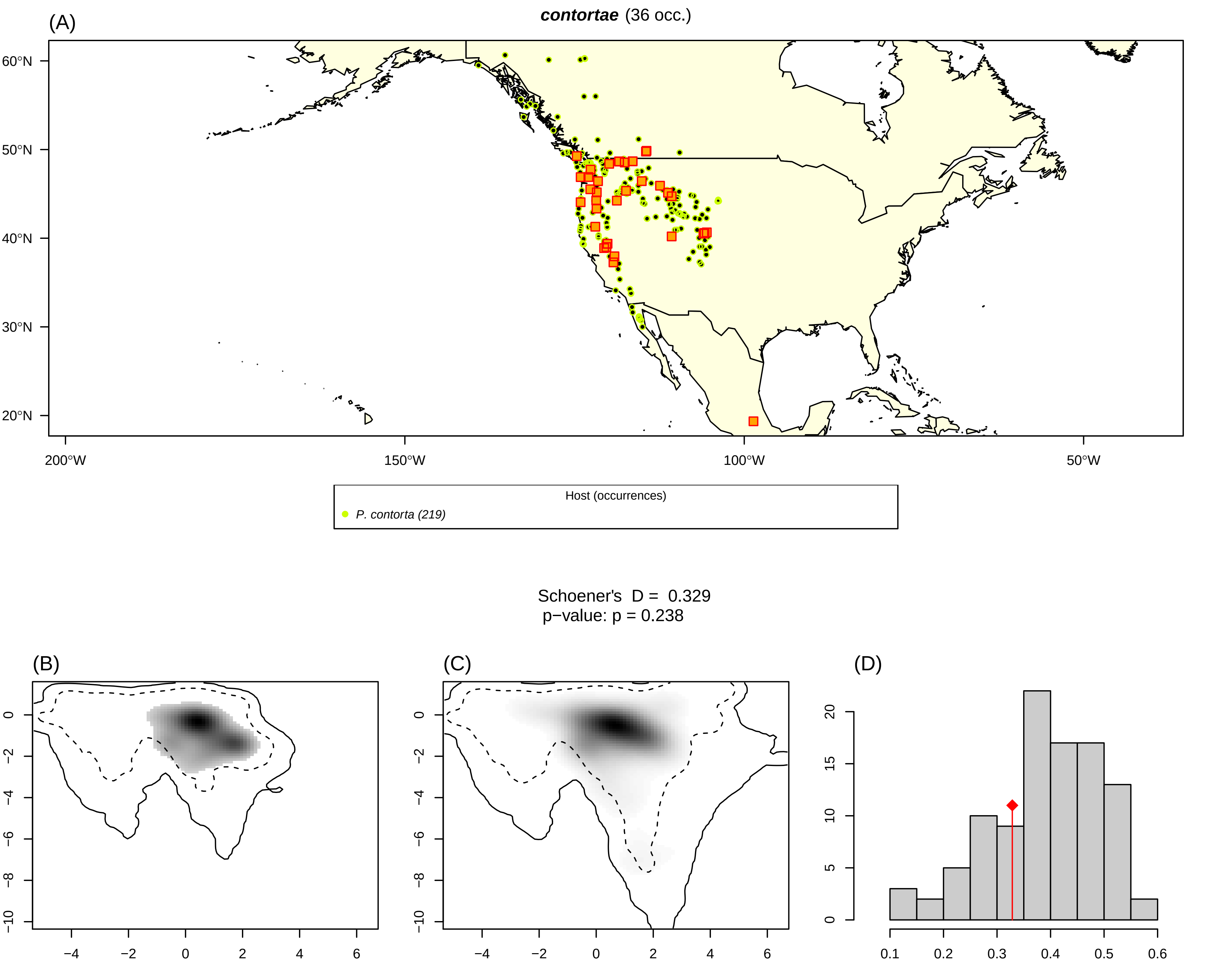

(A)
contortae (36 occ.)
60°N
50°N
40°N
30°N
20°N
200°W
150°W
100°W
50°W
Host (occurrences)
P. contorta (219)
Schoener's D = 0.329
p−value: p = 0.238
(B)
(C)
(D)
0
−2
−4
−6
−8
−10
−4
−2
0
2
4
6
0
5
10
15
20
0.1
0.2
0.3
0.4
0.5
0.6

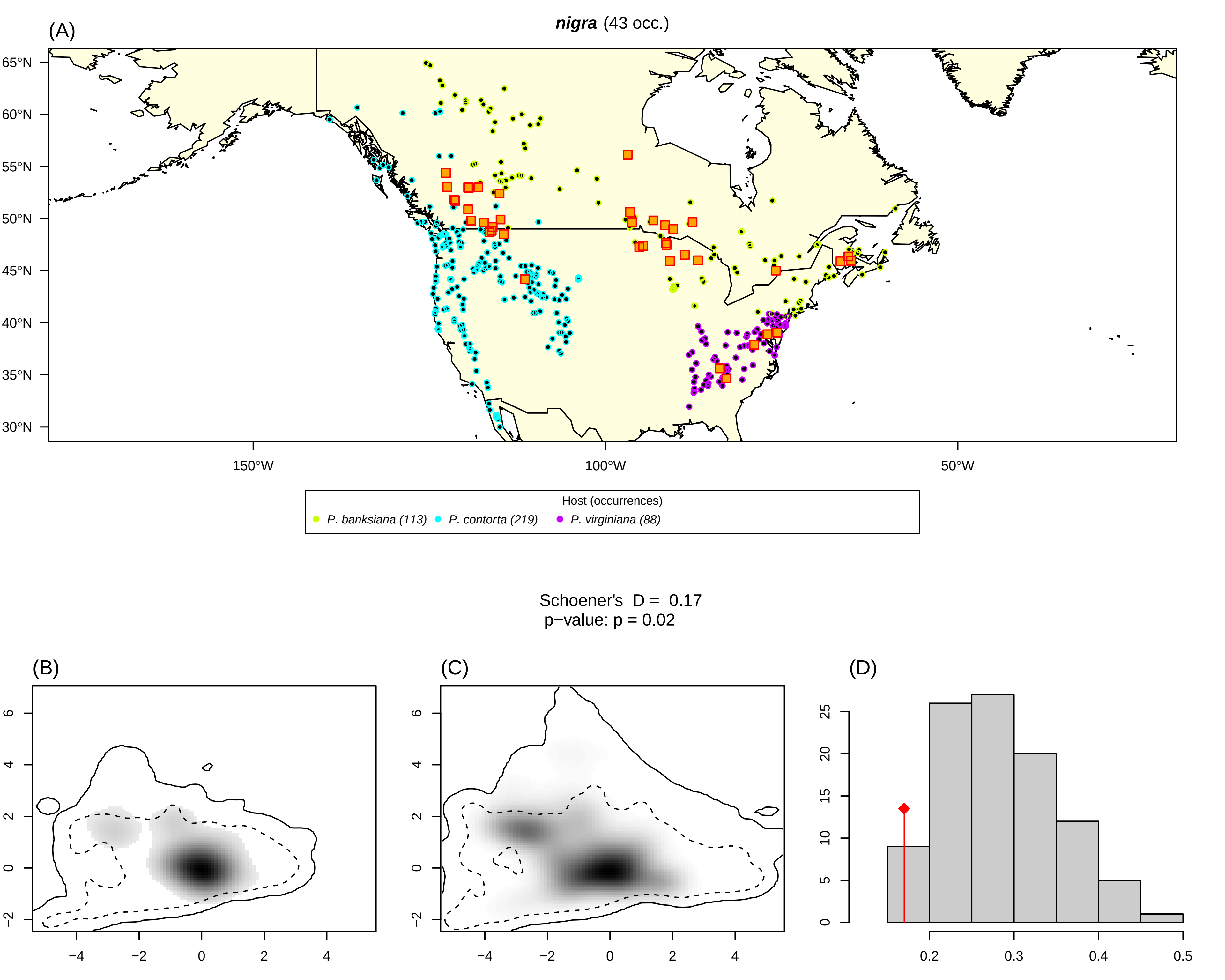
(A)
nigra (43 occ.)
65°N
60°N
55°N
50°N
45°N
40°N
35°N
30°N
150°W
100°W
50°W
Host (occurrences)
P. banksiana (113)
P. contorta (219)
P. virginiana (88)
Schoener's D = 0.17
p−value: p = 0.02
(B)
(C)
(D)
−4
−2
0
2
4
6
0.2
0.3
0.4
0.5
5
10
15
20
25

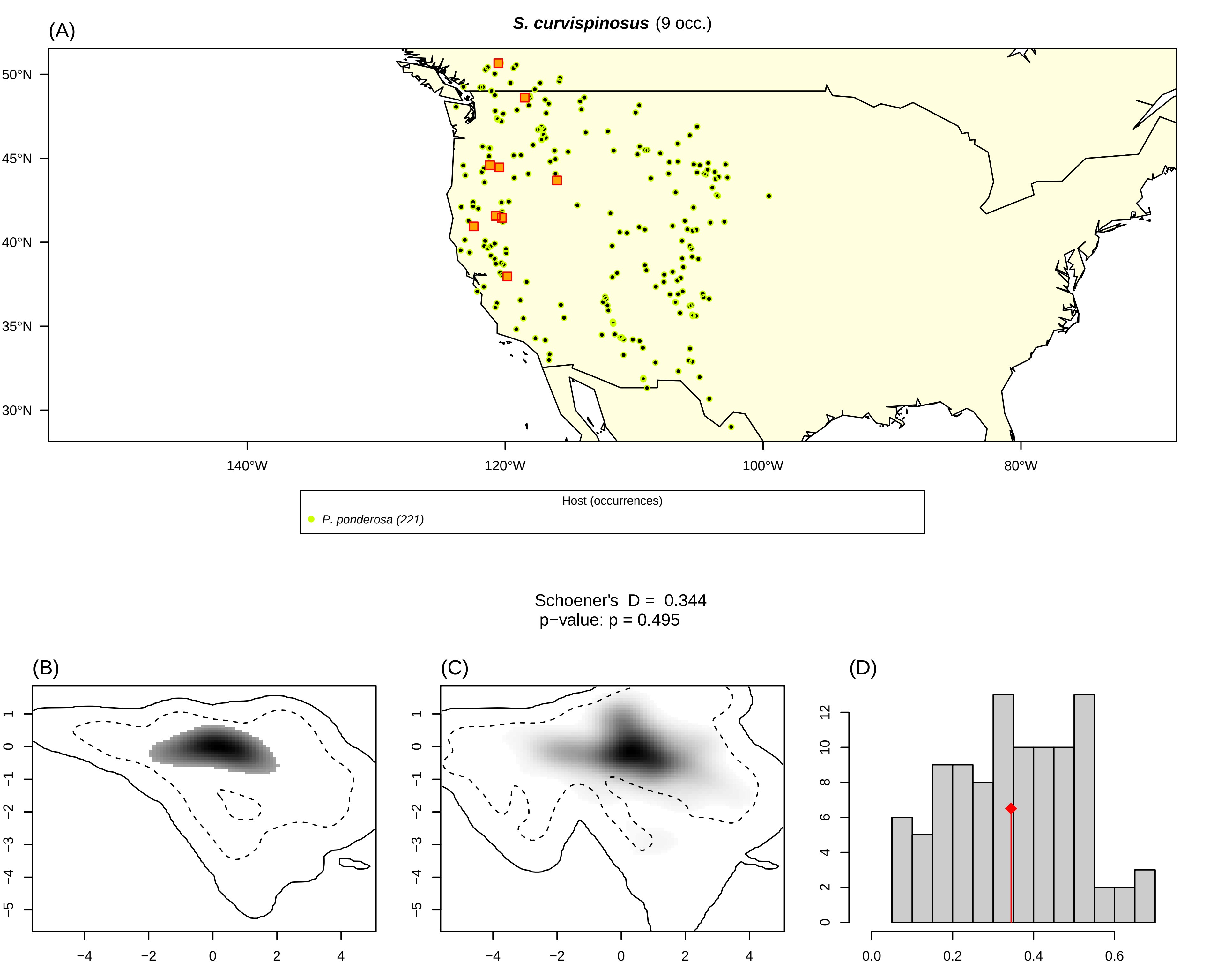
S. curvispinosus (9 occ.)
(A)
50°N
45°N
40°N
35°N
30°N
140°W
120°W
100°W
80°W
Host (occurrences)
P. ponderosa (221)
Schoener's D = 0.344
p−value: p = 0.495
(B)
(C)
(D)
1
0
−1
−2
−3
−4
−5
−4
−2
0
2
4
0
2
4
6
8
10
12
0.0
0.2
0.4
0.6

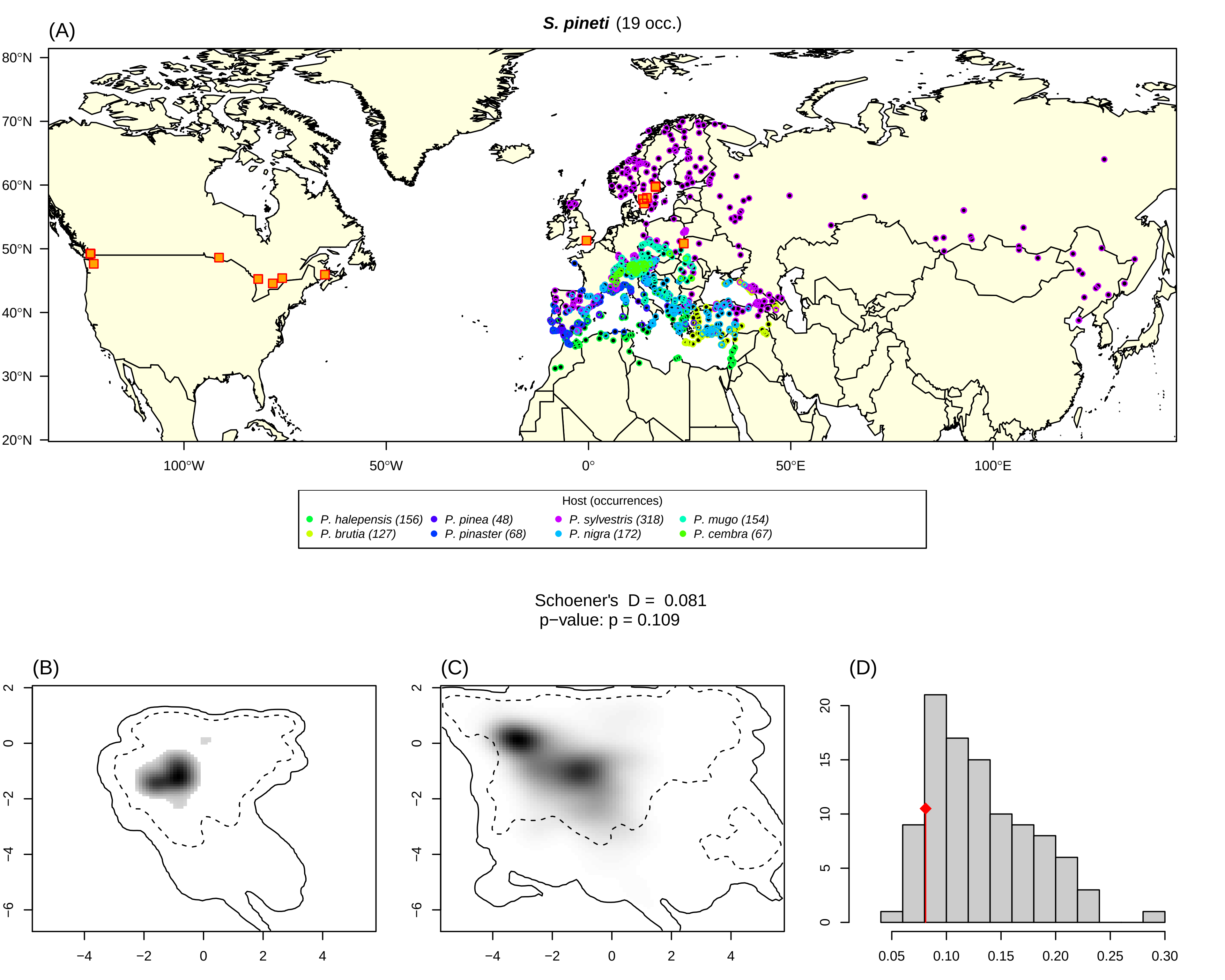
(A)
S. pineti (19 occ.)
80°N
70°N
60°N
50°N
40°N
30°N
20°N
100°W
50°W
0°
50°E
100°E
Host (occurrences)
P. halepensis (156)
P. pinea (48)
P. sylvestris (318)
P. mugo (154)
P. brutia (127)
P. pinaster (68)
P. nigra (172)
P. cembra (67)
Schoener's D = 0.081
p−value: p = 0.109
(B)
(C)
(D)
2
0
−2
−4
−6
−4
−2
0
2
4
20
15
10
5
0
0.05
0.10
0.15
0.20
0.25
0.30

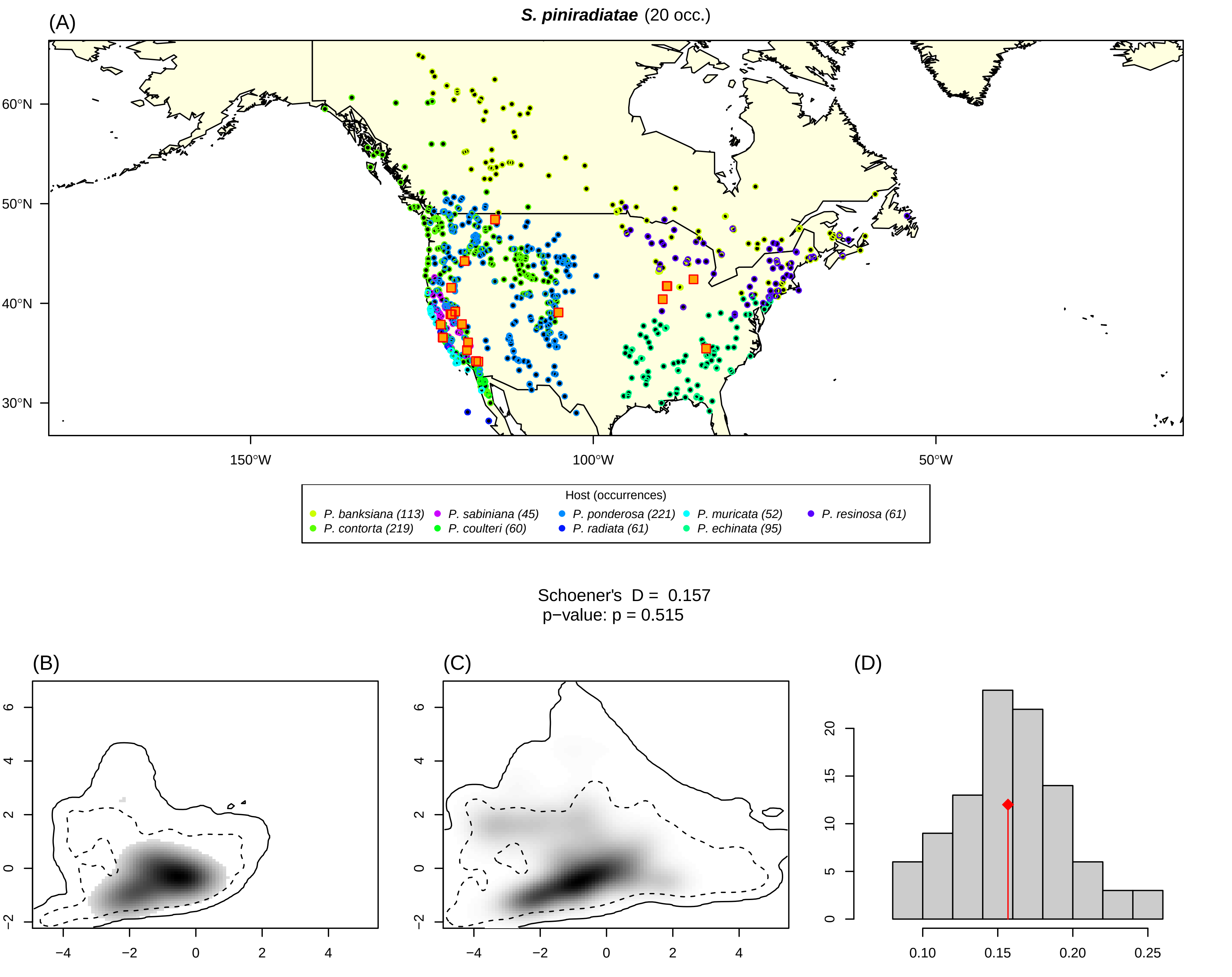

S. piniradiatae (20 occ.)
(A)
60°N
50°N
40°N
30°N
150°W
100°W
50°W
Host (occurrences)
P. banksiana (113)
P. contorta (219)
P. sabiniana (45)
P. coulteri (60)
P. ponderosa (221)
P. radiata (61)
P. muricata (52)
P. echinata (95)
P. resinosa (61)
Schoener's D = 0.157
p−value: p = 0.515
(B)
(C)
(D)

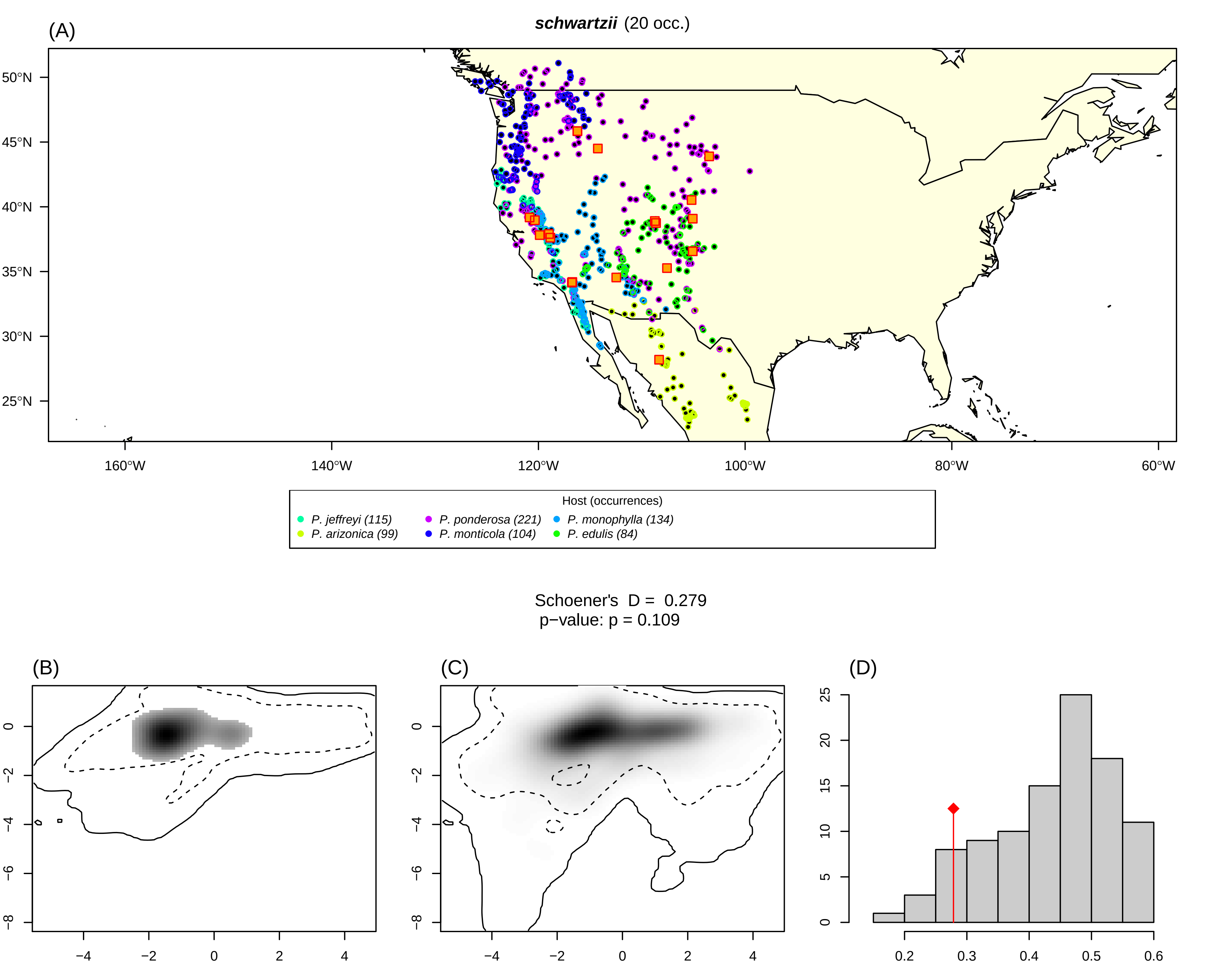
(A)
schwartzii (20 occ.)
50°N
45°N
40°N
35°N
30°N
25°N
160°W
140°W
120°W
100°W
80°W
60°W
Host (occurrences)
P. jeffreyi (115)
P. ponderosa (221)
P. monophylla (134)
P. arizonica (99)
P. monticola (104)
P. edulis (84)
Schoener's D = 0.279
p−value: p = 0.109
(B)
(C)
(D)
0
−2
−4
−6
−8
−4
−2
0
2
4
0
5
10
15
20
25
0.2
0.3
0.4
0.5
0.6
