# Supplementary figures and images for: The evolution of climate tolerance in conifer‐feeding aphids in relation to their host's climatic niche
Source: Ecol Evol. 2019 Oct 2;9(20):11657–71. doi: 10.1002/ece3.5652 (PMC6822038; doi:10.1002/ece3.5652)

# DTT - BIO 5

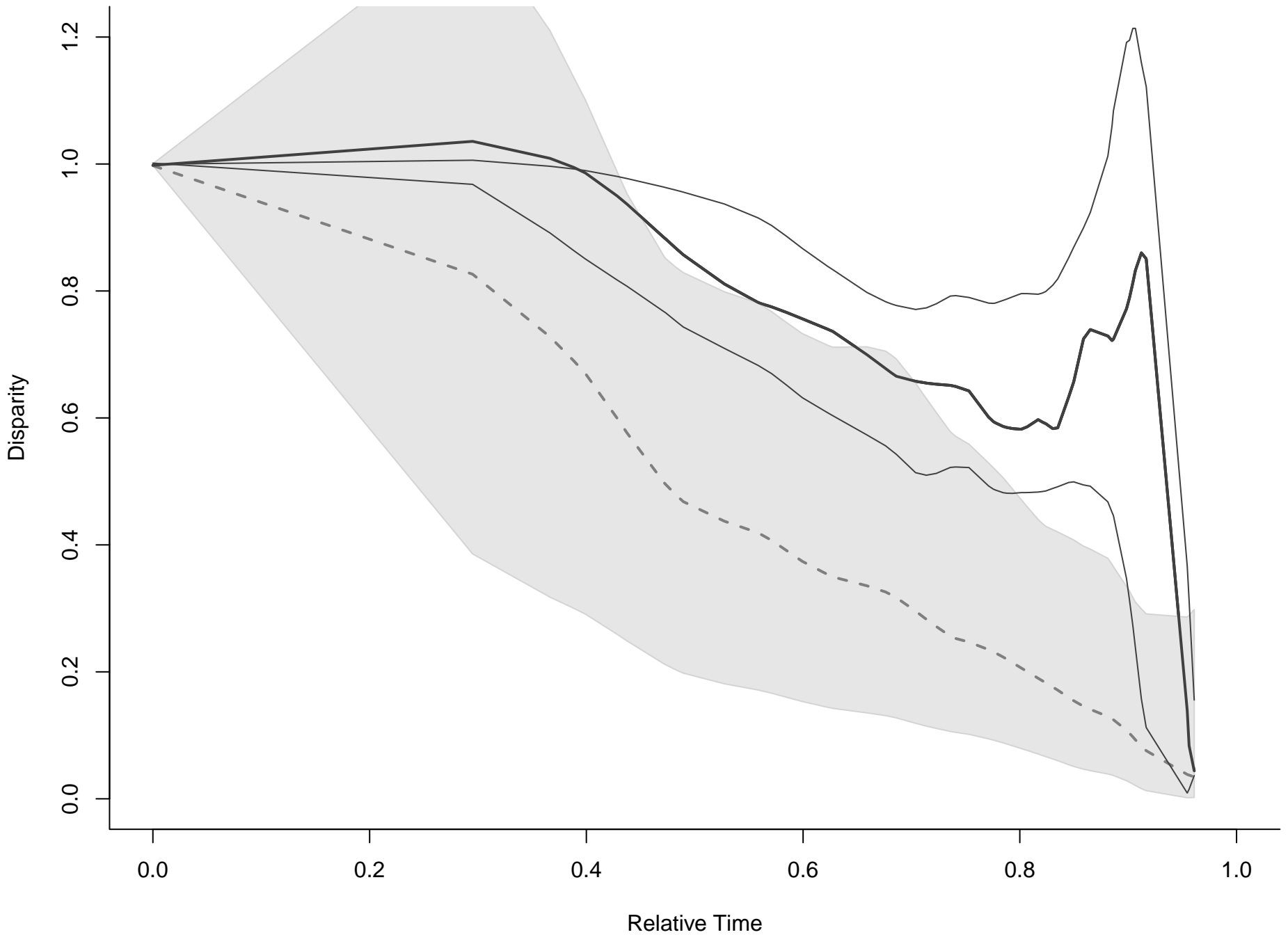

# DTT - BIO 6

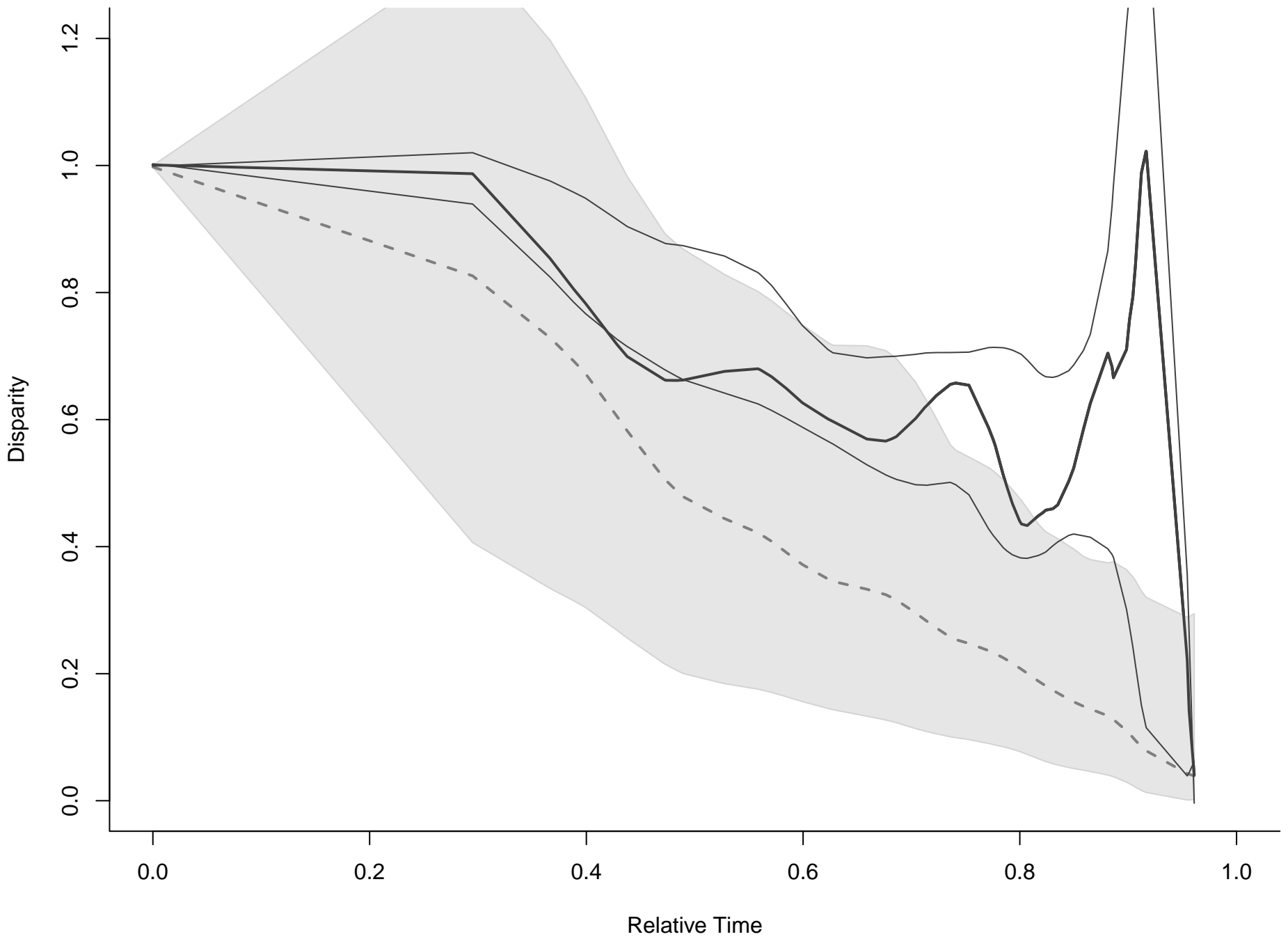

# DTT - BIO 10

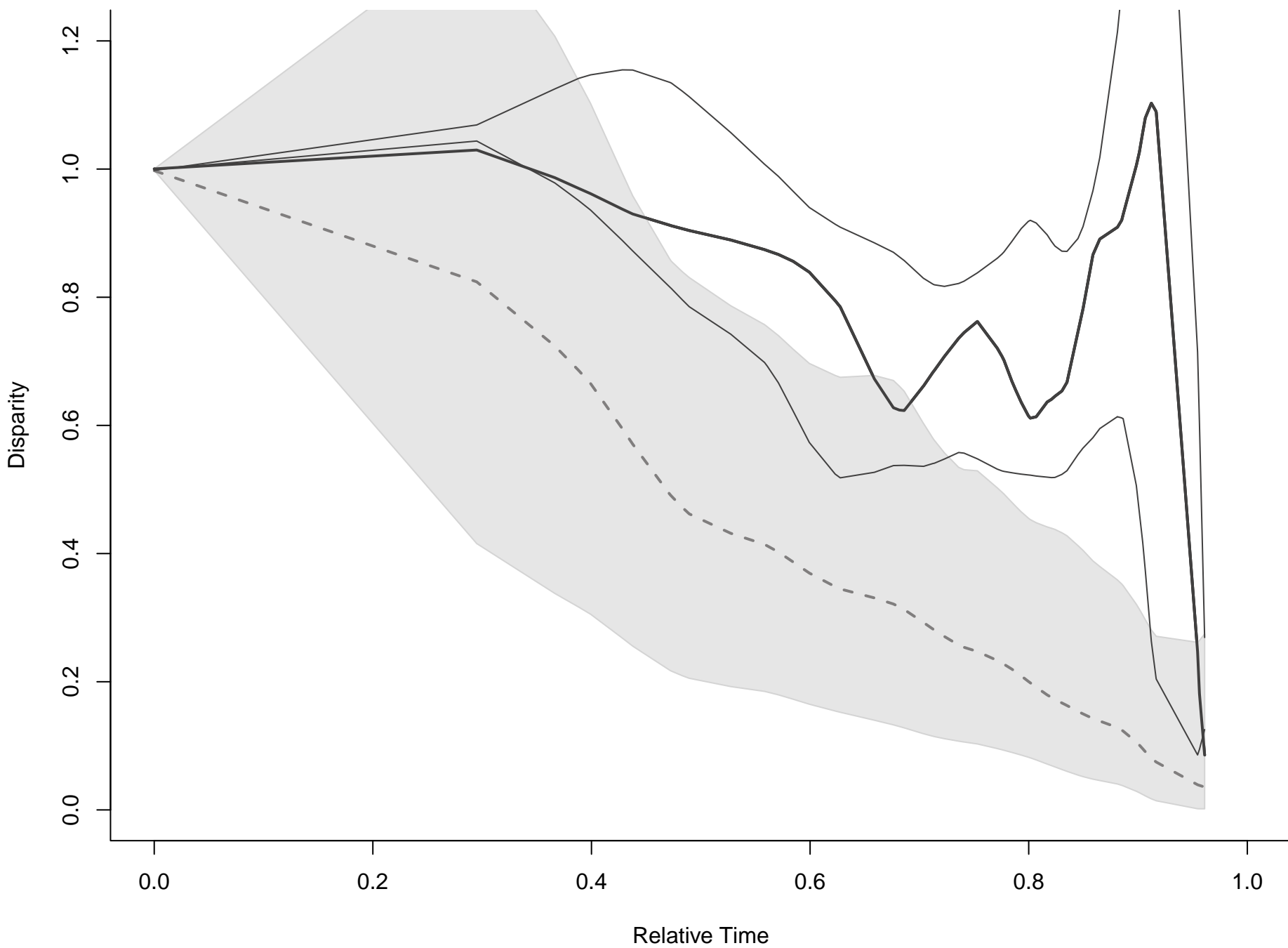

# DTT - BIO 11

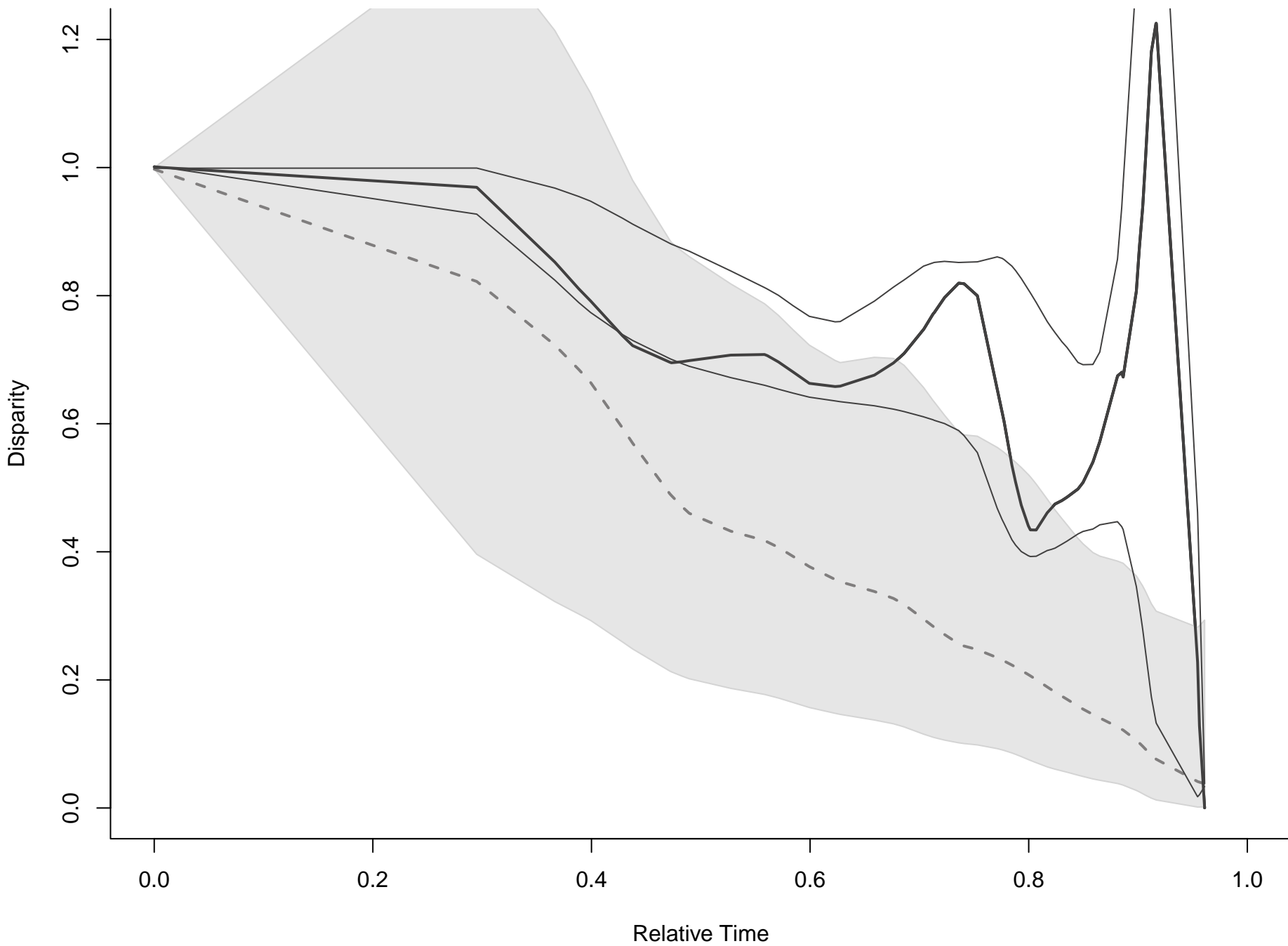

# DTT - BIO 17

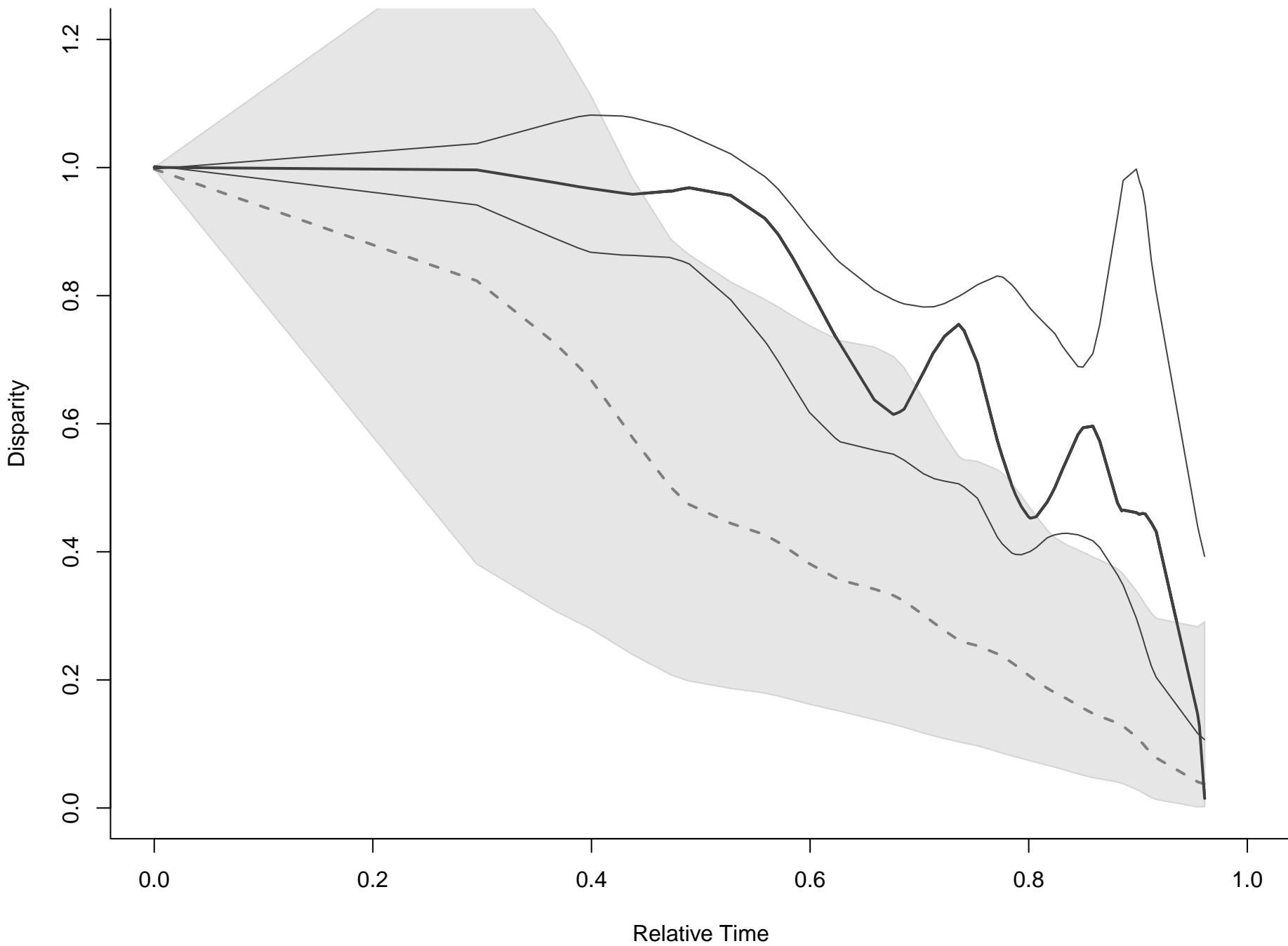

Supplement: Supplementary file 7 [file ECE3-9-11657-s007.pdf]

All Cinara occurrences

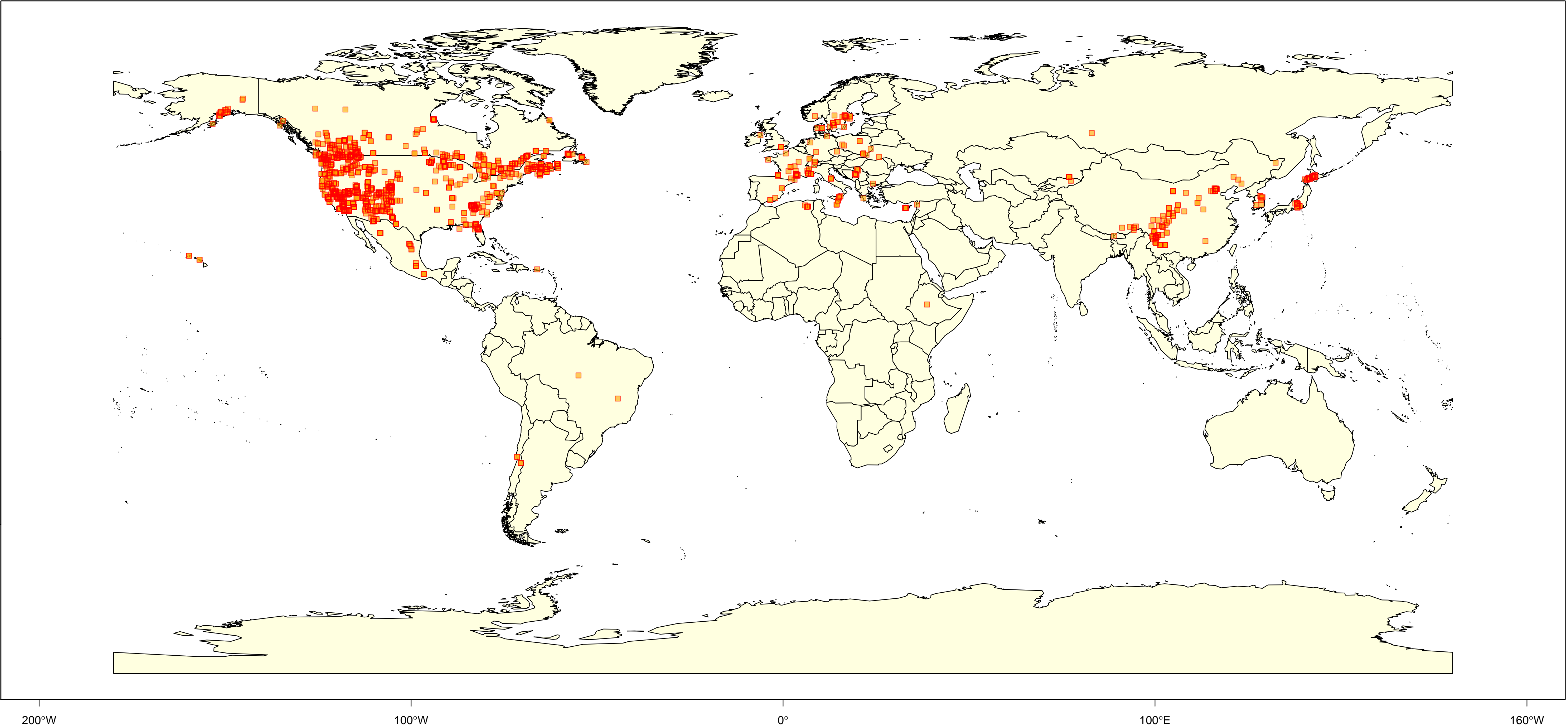

Supplement: Supplementary file 8 [file ECE3-9-11657-s008.pdf]
