# Supplementary material for: The evolution of climate tolerance in conifer‐feeding aphids in relation to their host's climatic niche
Source: Ecol Evol. 2019 Oct 2;9(20):11657–71. doi: 10.1002/ece3.5652 (PMC6822038; doi:10.1002/ece3.5652)
Supplement: Supplementary file 3 [file ECE3-9-11657-s003.pdf]

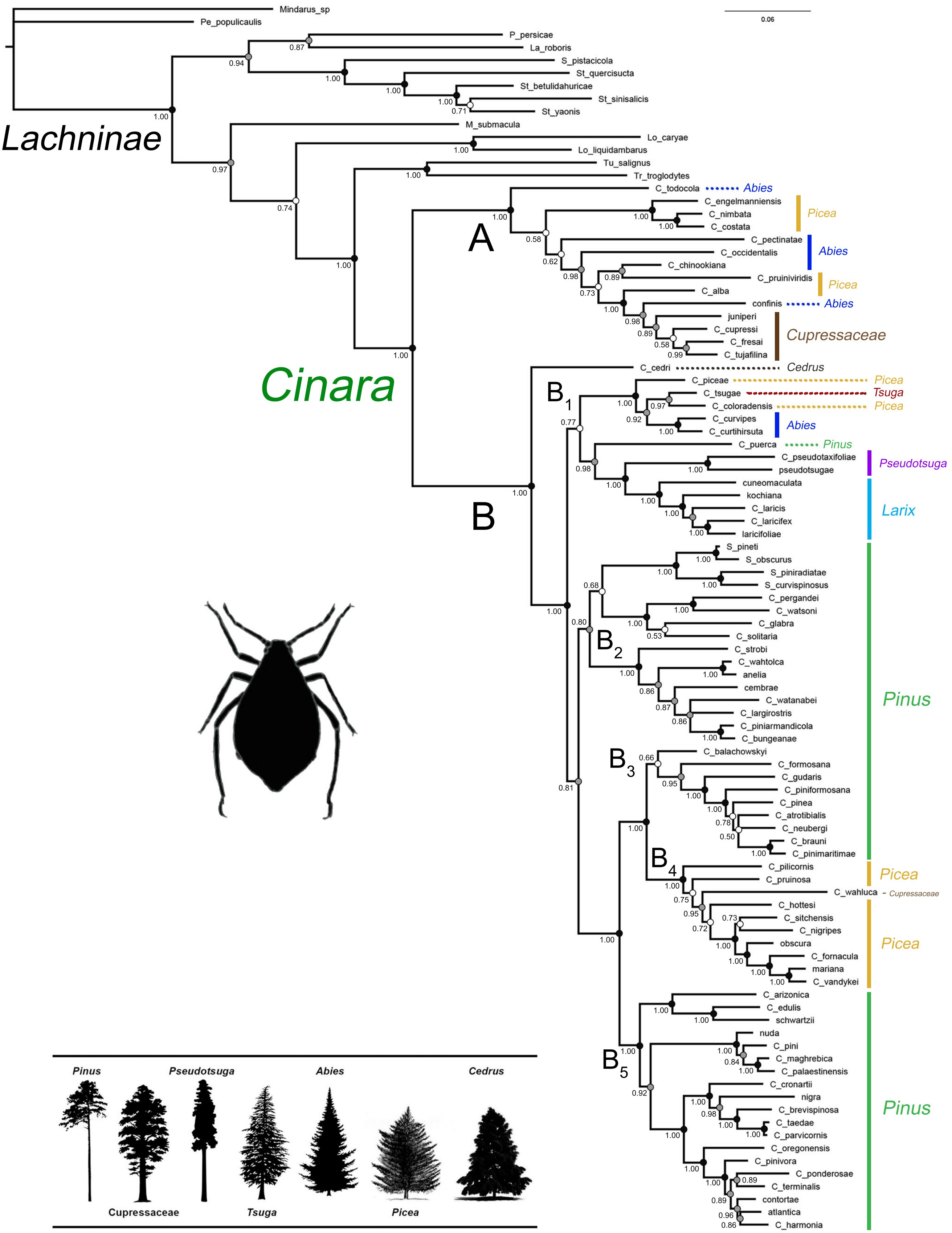

Appendix 3: Phylogeny of Cinara infered with MrBayes 3.2.3. Posterior probabilities (PP) are indicated for each node. White points indicate PP below 0.8, grey indicate PP superior to 0.8 and inferior to 1 while black points indicate PP equal to 1. The insect silhouette represents a Cinara species. The range of host species of Cinara is indicated on the right hand side.
